# NIR‐Activatable Domino Cascade Catalysis Nanozyme Reactor for Multi‐Mechanism Synergistic Immunotherapy in Bladder Cancer

**DOI:** 10.1002/advs.202513913

**Published:** 2025-12-12

**Authors:** Yongnan Jiang, Qingling Zhang, Yuhan Zhang, Xinlu Yu, Bo Jia, Yulong Dong, Yalong Wu, Kelong Fan, Xinquan Gu, Lei Ji, Wei Jiang, Bin Liu

**Affiliations:** ^1^ Department of Urology China‐Japan Union Hospital of Jilin University Changchun Jilin 130033 China; ^2^ Department of Cardiology China‐Japan Union Hospital of Jilin University Changchun Jilin 130033 China; ^3^ Department of Dermatology China‐Japan Union Hospital of Jilin University Changchun Jilin 130033 China; ^4^ Department of Otolaryngology Second Hospital of Jilin University Changchun Jilin 130041 China; ^5^ State Key Laboratory of Metabolic Dysregulation & Prevention and Treatment of Esophageal Cancer Tianjian Laboratory of Advanced Biomedical Sciences Academy of Medical Sciences Zhengzhou University Zhengzhou 450052 China; ^6^ CAS Engineering Laboratory for Nanozyme Key Laboratory of Biomacromolecules (CAS) CAS Center for Excellence in Biomacromolecules Institute of Biophysics Chinese Academy of Sciences Beijing 100101 China

**Keywords:** ferroptosis, immune infiltration, intravesical instillation, nanozyme, Thermo‐responsive hydrogel

## Abstract

Current intravesical therapies for bladder cancer after resection are limited by poor tissue penetration, off‐target effects, and insufficient efficacy. To address these challenges, this study designs a thermo‐responsive hydrogel (PNH) that encapsulates chitosan (CS)‐coated Fe/Mn bimetallic nanozymes (FMCC) together with cholesterol oxidase (ChOx). FMCC displays multiple enzyme‐mimicking activities, including peroxidase (POD), catalase (CAT), and glutathione oxidase (GSHox). ChOx amplifies this catalytic cascade, enhancing reactive oxygen species (ROS) production and inducing ferroptosis‐mediated tumor cell death. The CS coating improves mucosal adhesion and tissue permeability, thereby facilitating intravesical delivery. Upon near‐infrared (NIR) irradiation, FMCC generates heat that liquefies the hydrogel, enabling spatiotemporally controlled drug release and providing mild photothermal therapy (MPTT). This photothermal effect acts synergistically with ferroptosis induction and immune modulation, concurrently minimizing damage to normal tissues. In parallel, ChOx disrupts cholesterol‐rich membrane rafts and promotes pro‐inflammatory M1 macrophage polarization. Released Mn^2+^ ions further potentiate immune activation by stimulating the cGAS–STING pathway, driving IFN‐β and IL‐6 secretion, dendritic cell maturation, and T cell infiltration. Together, this nanozyme–hydrogel system integrates tissue penetration, metabolic disruption, and immune stimulation, representing a promising strategy for localized bladder cancer therapy.

## Graphical abstract

This study presents an intravesical delivery strategy of ferroptosis‐ and immunity‐inducing nanozyme‐loaded thermoresponsive hydrogels (FMCC@PNH) for enhanced bladder cancer therapy.

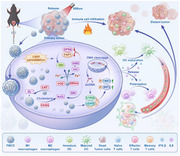

## 1 Introduction

Bladder cancer ranks among the ten most common malignancies in men worldwide and presents significant clinical challenges due to its high recurrence rate and poor prognosis.^[^ ^1^, ^2^ ^]^ Current clinical management typically involves complete transurethral resection of bladder tumor, followed by intravesical instillation of chemotherapeutic agents such as mitomycin C, epirubicin, pirarubicin, or gemcitabine.^[^ ^3^ ^]^ Nevertheless, the dense glycosaminoglycan layer and tight intercellular junctions of the bladder urothelium constitute formidable barriers that limit the efficacy of intravesical instillation therapy.^[^ ^4^ ^]^ Consequently, drug penetration is severely restricted, necessitating repeated high‐dose administration, which often leads to local irritation (e.g., urgency, dysuria, and hematuria), poor patient compliance, and the emergence of drug resistance. Furthermore, conventional instillation approaches rarely induce robust anti‐tumor immunity, ultimately resulting in frequent tumor recurrence and disease progression.^[^ ^5^ ^]^

Near‐infrared (NIR) lasers (808–1064 nm) can be readily integrated into cystoscopy via existing fiber‐optic channels, enabling localized and repeatable intravesical irradiation.^[^ ^6^ ^]^ This wavelength window penetrates tissue efficiently while minimizing off‐target damage, and several systems are already FDA/CE approved for other indications. These features underscore the clinical feasibility of NIR‐assisted cystoscopy as a minimally invasive approach for both diagnosis and therapy.^[^ ^7^, ^8^ ^]^

Building upon this clinical feasibility, the integration of NIR‐triggered photothermal therapy (PTT) with catalytic nanomedicine has emerged as a promising strategy to enhance intravesical treatment efficacy. Nanozymes, owing to their intrinsic enzyme‐mimicking catalytic activities, structural stability, and tunable surface chemistry, have demonstrated remarkable potential in tumor therapy.^[^ ^9^ ^]^ They can efficiently generate reactive oxygen species (ROS) and remodel the tumor microenvironment (TME). The biochemical features of the TME, characterized by elevated hydrogen peroxide (H_2_O_2_), high glutathione (GSH), and hypoxia, provide a conducive environment for catalytic nanomedicine. For example, cholesterol oxidase (ChOx) catalyzes the oxidation of cholesterol into cholestenone and H_2_O_2_, triggering oxidative stress and disrupting cell membranes. Inorganic nanozymes mimicking peroxidase (POD), catalase (CAT), and glutathione oxidase (GSHox) activities can further amplify ROS generation, deplete GSH, and alleviate hypoxia, thereby enhancing therapeutic outcomes across various cancer types and promoting antitumor immune responses.^[^ ^9^, ^10^ ^]^ Despite these advantages, the clinical translation of nanozymes in bladder cancer remains hindered by limited catalytic specificity, potential off‐target toxicity, and poor penetration through the dense urothelial barrier.^[^ ^11^ ^]^ To overcome these limitations, small‐sized nanozymes have been developed to enhance mucosal penetration and retention (EPR), thus improving localized drug delivery efficiency.^[^ ^12^, ^13^, ^14^, ^15^ ^]^ Moreover, combining nanozymes with stimuli‐responsive hydrogels enables spatiotemporally controlled drug release.^[^ ^16^ ^]^ Thermosensitive hydrogels composed of *N*‐isopropylacrylamide (NIPAM) and *N*‐hydroxymethylacrylamide (NHMAM) can encapsulate nanozymes and undergo rapid sol–gel transitions under NIR irradiation, allowing controlled, localized release and prolonged bladder residence. Simultaneously, NIR‐induced photothermal effects not only trigger hydrogel contraction but also enhance tissue permeability and accelerate catalytic reactions, achieving tumor‐selective activation of nanozymes.^[^ ^17^, ^18^, ^19^ ^]^ This integrated approach improves therapeutic efficacy while minimizing systemic toxicity in bladder cancer treatment.

Beyond ROS‐mediated cytotoxicity, ferroptosis has recently emerged as another key mechanism contributing to nanozyme‐based chemodynamic therapy. Ferroptosis is a regulated form of cell death driven by iron‐dependent lipid peroxidation initiated by hydroxyl radicals (•OH) generated through the Fenton reaction between Fe^2+^ and H_2_O_2_.^[^ ^20^ ^]^ However, tumor cells often resist ferroptosis by upregulating antioxidant defenses such as GSH, glutathione peroxidase 4 (GPX4), and ferroptosis suppressor protein 1 (FSP1).^[^ ^21^, ^22^ ^]^ Moreover, cholesterol further contributes to ferroptosis resistance and immune evasion by stabilizing lipid rafts, reducing membrane fluidity, and inhibiting lipid peroxidation. In the TME, cholesterol accumulation promotes immunosuppression by driving CD8^+^ T cell exhaustion and skewing macrophages toward an M2 phenotype.^[^ ^23^, ^24^, ^25^ ^]^ Therefore, cholesterol depletion not only reprograms tumor‐associated macrophages (TAMs) into a pro‐inflammatory M1 state and restores antitumor immunity, but also benefits nanozyme‐mediated chemodynamic therapy in the TME by enhancing catalytic efficiency and immune infiltration.^[^ ^25^, ^26^, ^27^ ^]^ Importantly, ferroptosis‐induced cellular damage can further augment immune activation. The release of cytosolic double‐stranded DNA (dsDNA) from ruptured nuclei serves as a danger signal to activate the cGAS‐STING pathway, which in turn induces type I interferons and proinflammatory cytokines through TBK1–IRF3 signaling.^[^ ^28^, ^29^, ^30^ ^]^ Moreover, manganese ions (Mn^2^⁺) can potentiate this process by promoting cGAS–dsDNA binding and STING oligomerization, thereby amplifying innate and adaptive immune responses. By integrating iron‐mediated ferroptosis, cholesterol depletion, and STING activation, catalytic nanoplatforms can achieve synergistic chemodynamic and immunomodulatory effects, offering a multifaceted therapeutic strategy for bladder cancer.^[^ ^30^, ^31^ ^]^

**Scheme 1:**
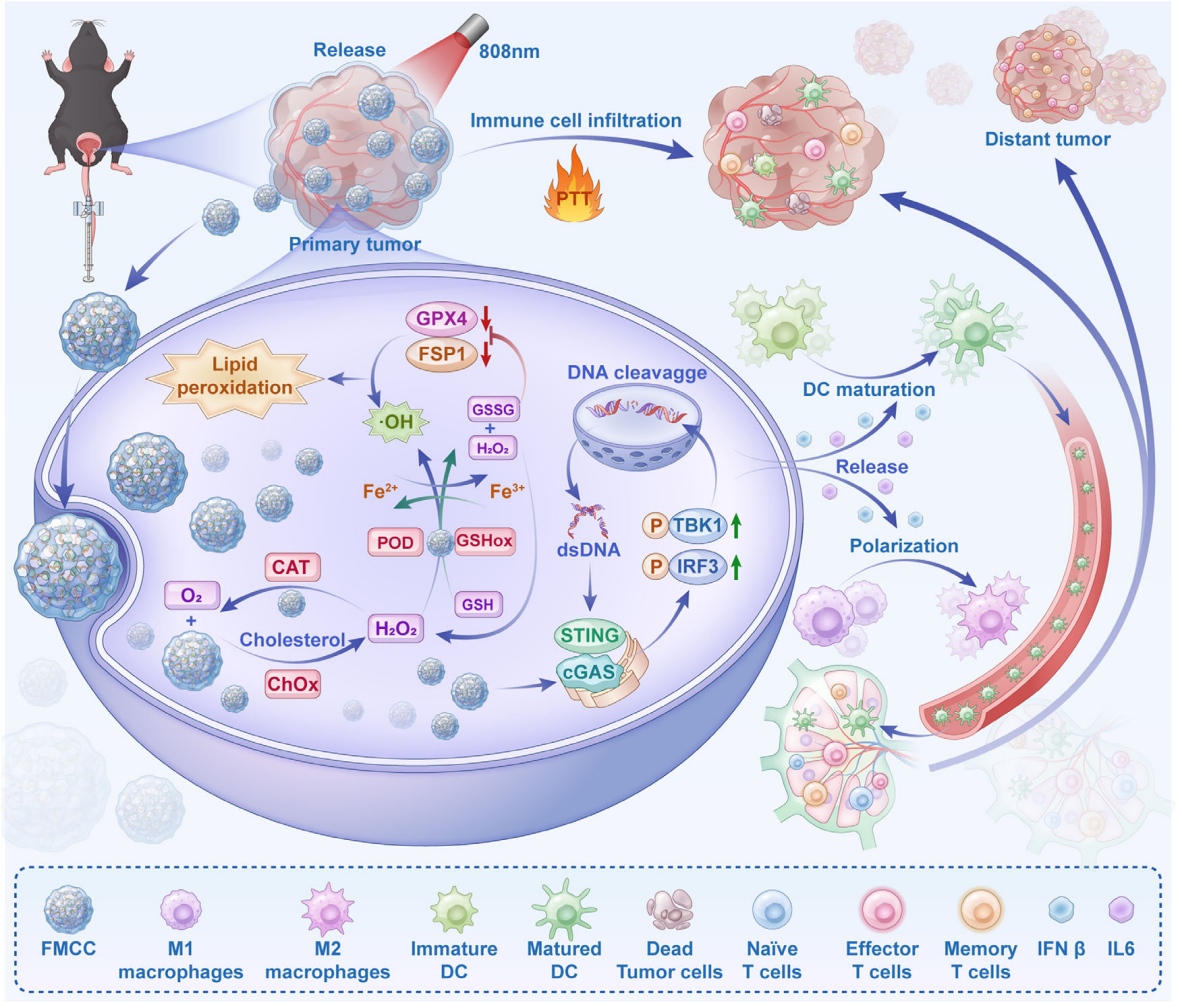
Upon intravesical administration, NIR (808 nm) irradiation triggers mild photothermal heating (PTT) of FMCC@PNH, inducing phase transition of the thermosensitive hydrogel and controlled release of Fe/Mn@CN‐ChOx‐CS (FMCC) nanozymes. Released FMCC efficiently penetrates bladder tumors and exerts cascade catalytic effects. Cholesterol oxidase (ChOx) catalyzes cholesterol oxidation to generate H_2_O_2_, which is further decomposed by POD‐ and CAT‐like activities into •OH and O_2_, inducing oxidative damage while alleviating hypoxia. GSHox‐like activity depletes GSH, inhibiting GPX4/FSP1 and amplifying ferroptosis. The resulting DNA damage and Mn^2+^ release synergistically activate the cGAS–STING pathway, leading to phosphorylation of TBK1/IRF3 and secretion of IFN‐β and IL‐6. These cytokines promote dendritic cell (DC) maturation, M1 macrophage polarization, and infiltration of CD8^+^ T, NK, and central memory T (TCM) cells, thereby establishing systemic antitumor immunity and suppressing tumor recurrence and metastasis.

In this study, we developed a multifunctional nanozyme termed Fe/Mn@CN‐ChOx‐CS (FMCC), synthesized via stepwise assembly of Fe/Mn co‐doped carbon nanospheres, followed by loading of natural ChOx and surface stabilization with chitosan (CS).^[^ ^32^ ^]^ By optimizing the relative content of each constituent, FMCC exhibited enhanced mucosal penetration, robust catalytic activity, and favorable photothermal properties. To further achieve thermally triggered and localized delivery, we synthesized a co‐polymer hydrogel PNIPAM‐co‐NHMAM (PNH) with a lower critical solution temperature (LCST) that matched FMCC's photothermal heating profile. Under 808 nm NIR irradiation, FMCC@PNH rapidly warms to ≈45 °C, thereby inducing a reversible hydrogel phase transition and triggering the rapid release of FMCC. This mild hyperthermic range not only suppresses glycolysis and nucleic acid synthesis in thermosensitive tumor cells but is further intensified by poor heat dissipation resulting from the abnormal tumor vasculature.^[^ ^33^ ^]^ Once released, FMCC nanoparticles penetrate deeply into the tumor tissue and exert a cascade of multimodal therapeutic effects. Specifically, ChOx catalyzes cholesterol oxidation, thereby disrupting lipid rafts and producing H_2_O_2_; subsequently, the POD‐like activity converts H_2_O_2_ to •OH, amplifying oxidative damage; Meanwhile, the CAT activity decomposes H_2_O_2_ into O_2_, alleviating hypoxia and sustaining ChOx‐mediated catalysis. In parallel, the GSHox‐like activity depletes GSH, inhibiting the GPX4/FSP1 pathway and sensitizing cells to ferroptosis. Moreover, the resultant DNA damage elicits cGAS‐STING pathway activation, which is further potentiated by Mn^2+^ ions released from FMCC. This leads to elevated IFN‐β and IL‐6 secretion, thereby promoting dendritic cells (DCs) maturation and enhancing CD8⁺ T‐cell recruitment. Collectively, the therapy reprograms the TME by inducing M1 macrophage polarization, increasing infiltration of natural killer (NK) cells and DCs, and expanding central memory T (TCM) cells, ultimately establishing durable systemic immunity and suppressing metastatic progression. A schematic illustration of the overall therapeutic mechanism is provided in Scheme 1.

Altogether, the FMCC@PNH platform integrates PTT, chemodynamic therapy, ferroptosis induction, and immune activation, offering a synergistic and spatiotemporally controllable strategy for effective intravesical bladder cancer treatment and systemic anti‐tumor immune enhancement.

## 2 Results and Discussion

### 2.1 Synthesis and Characterization of FMCC Nanozyme

**Figure 1:**
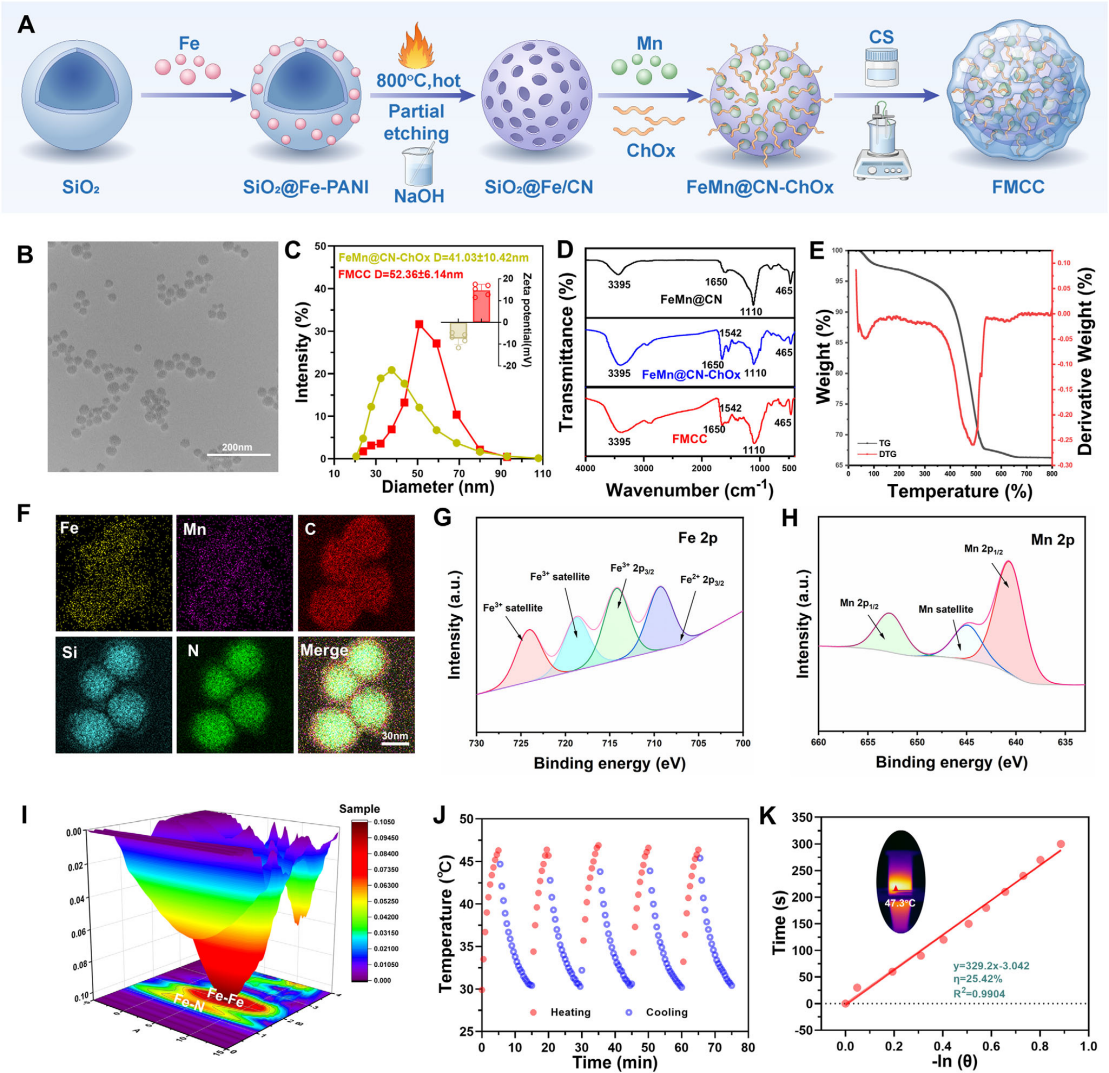
Structural design and physicochemical characterization of FMCC nanozyme. A) Schematic diagram of the synthesis route of FMCC. B) TEM observation of FMCC. C) Hydrodynamic diameter and zeta potential of FeMn@CN‐ChOx and FMCC D) FTIR spectra of FeMn@CN, FeMn@CN‐ChOx, and FMCC, confirming successful loading of ChOx and CS. E) Thermogravimetric analysis (TGA) and corresponding derivative thermogravimetric (DTG) curves of FMCC. F) Elemental mapping of FMCC nanoparticles. G,H) High‐resolution XPS spectra of (G) Fe 2p and (H) Mn 2p regions of FMCC. I) 3D Fourier‐transformed EXAFS spectrum of FMCC at the Fe K‐edge, indicating distinct Fe‐N and Fe‐Fe coordination environments. J) The temperature change of FMCC during multiple heating and cooling cycles under 808 nm near‐infrared light irradiation. K) The ‐ln(*θ*) versus time curve, the inset image shows the thermal imaging of the material under near‐infrared irradiation.

As shown in **Figure** **1**A, FMCC was synthesized through SiO_2_ templating, aniline polymerization, FeCl_2_ doping/pyrolysis, and subsequent Mn^2+^ impregnation, followed by co‐loading of ChOx and CS. This process yielded hollow Fe–Mn/N‐doped carbon nanostructures with enhanced catalytic activity, stability, and mucosal adhesion. POD and CAT activity assays confirmed that the formulation containing 0.1 mm Mn^2+^ and 0.1% CS exhibited superior catalytic performance (Figures S1 and S2, Supporting Information). In this configuration, the 0.1% CS coating improved stability and mucoadhesion, and, being electrostatically adsorbed, it was relatively labile and thus imposed minimal interference with catalytic sites, preserving overall nanozyme activity for intravesical application.

Transmission electron microscopy (TEM) revealed uniform spherical particles (Figure 1B). Dynamic light scattering analysis showed an average hydrodynamic diameter of 52.36 nm and a zeta potential shift from negative to +14.83 mV after CS coating (Figure 1C), confirming the successful introduction of the chitosan layer and its enhanced interaction with the negatively charged bladder mucosa. Fourier‐transform infrared (FTIR) spectra (Figure 1D) showed characteristic amide bands (1650–1540 cm^−1)^ from ChOx and broad ─OH/─NH stretching (3434–3395 cm^−1)^), confirming the incorporation of protein and polysaccharide components. Elemental mapping demonstrated uniform Fe and Mn distribution (Figure 1F). Residual Si signals were detected after template removal, indicating partial core retention, which enhanced structural stability and loading capacity without impairing catalytic or photothermal performance. Thermogravimetric analysis (TGA, Figure 1E) revealed a three‐step mass loss: water evaporation (<150 °C), decomposition of organic components (400–500 °C), and stable inorganic residue (>500 °C), further confirming the successful integration of functional components.

X‐ray absorption fine structure (XAFS) confirmed a mixed‐valence Fe^2+^/Fe^3+^ state, with Fe K‐edge positions between Fe foil, Fe‐phthalocyanine, and Fe_2_O_3_ (Figure S3, Supporting Information). 3D Fourier‐transformed EXAFS (FT‐EXAFS, Figure 1I) showed coordination peaks at ≈1.5 Å (Fe─N) and ≈2.2 Å (Fe─Fe), indicating both atomically dispersed Fe sites and small Fe clusters. This dual‐site configuration, reinforced by Fe─Mn electronic interactions, is expected to enhance chemodynamic and ferroptosis‐related activity. X‐ray photoelectron spectroscopy (XPS) further verified the Fe^2+^/Fe^3+^ coexistence, with Fe 2p_3/2_ peaks at 709.5 and 711.5 eV (Figure 1G). The Mn 2p spectrum (Figure 1H) displayed a main peak at 641.3 eV with a clear satellite structure, consistent with Mn^2+^ (3d^5^). FMCC dispersions (200 µg mL^−1^) exhibited rapid photothermal heating under 808 nm irradiation (1 W cm^−2^), reaching 46.9 °C within 5 min and maintaining excellent reproducibility over five on/off cycles (Figure 1J). The photothermal conversion efficiency (η) was calculated from the recorded temperature profiles. The calculation followed the standard method:^[^ ^34^ ^]^

(1) $$\eta =\frac{hS(T_{\max,NPs}-T_{\max,solvent\;})}{I(1-10^{-A_{808}})}$$

(2) t=−τslnθ

(3) $$\theta =\frac{\left(T-T_{\mathrm{surr}}\right)}{\left(T_{\max,\mathrm{NPs}}-T_{\mathrm{surr}}\right)}$$

(4) $$hS=\frac{mdCd}{\tau s}$$

T_max_ and T_surr_ were 46.9 and 25 °C, respectively, for FMCC dispersions (200 µg mL^−1^). The absorbance at 808 nm was A808 = 1.65. The system time constant (τs) was 326.3 s. Parameters included the convective heat transfer coefficient (h), container surface area (S), incident laser power density (I = 1 W cm^−2^), and water mass (md = 0.5 g) with specific heat capacity (Cd = 4.2 J g^−1^ K^−1^). Based on these values, the photothermal conversion efficiency was calculated as η = 25.42% (Figure 1K), confirming the excellent photothermal performance of FMCC.

**Figure 2:**
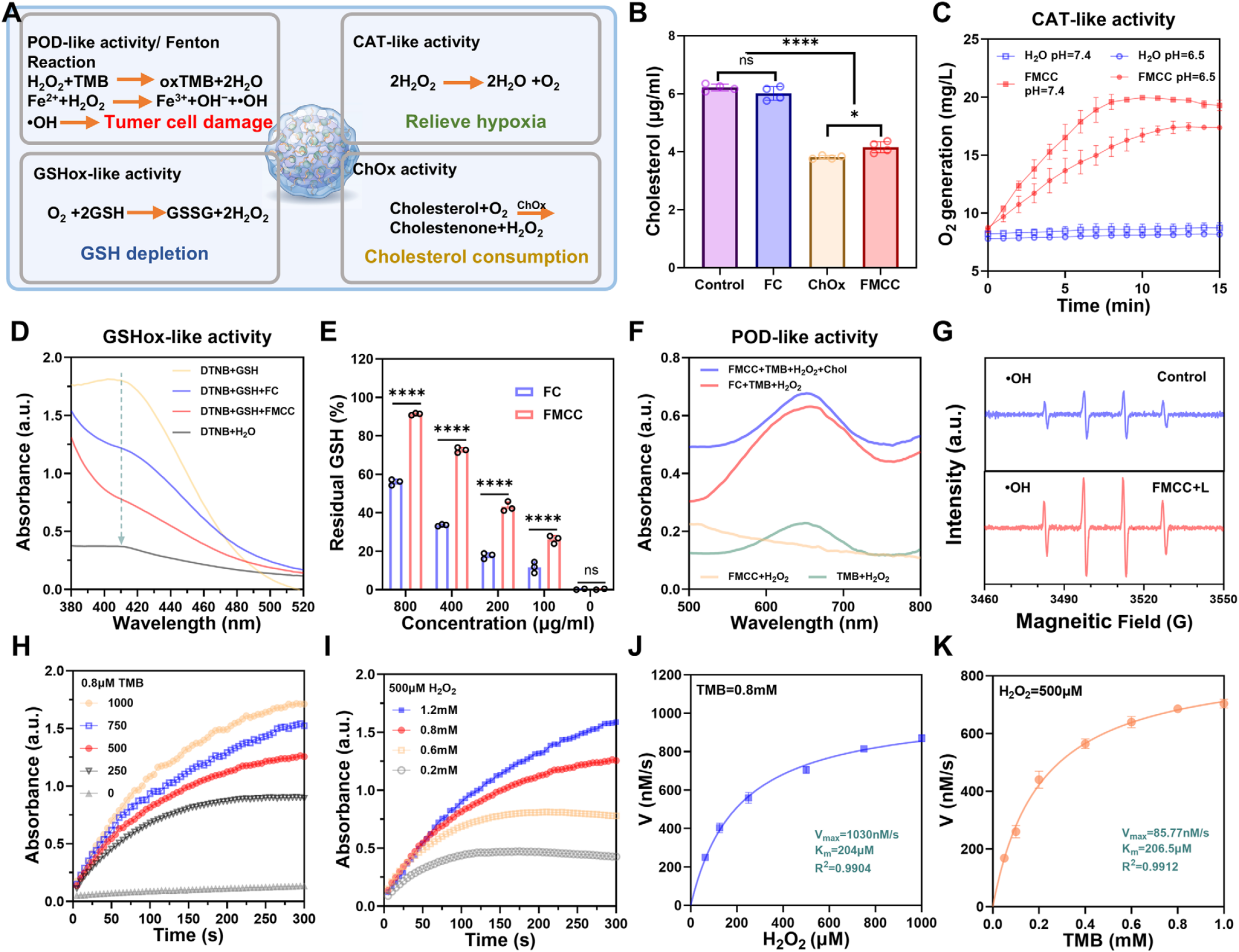
Multienzyme‐mimicking catalytic activities of FMCC nanozyme. A) Schematic illustration of FMCC's enzyme‐like functions (POD, CAT, GSHox, and ChOx). B) Quantification of residual cholesterol after treatment with different groups, confirming ChOx activity. C) CAT‐like activity evaluated by oxygen generation under pH 7.4 and 6.5. D) UV–vis spectra monitoring GSH depletion via DTNB assay, validating GSHox‐like activity. E) Quantification of residual GSH at different FMCC concentrations, showing enhanced GSH oxidation versus FC. F) UV–vis spectra showing POD‐like activity of FMCC. G) ESR spectra of •OH trapped by DMPO. H,I) Absorbance at 652 nm in the presence of varying concentrations of (H) TMB and (I) H_2_O_2_, confirming POD‐like catalytic performance. J,K) Michaelis–Menten kinetic curves of FMCC using (J) TMB and (K) H_2_O_2_ as the substrate. ^*^ *p* <0.05. ^**^ *p* <0.01. ^***^ *p* <0.001. ^****^ *p* <0.0001. “ns” denotes no significant difference.

FMCC exhibited strong multi‐enzyme‐mimicking activities, including POD, CAT, and GSHox‐like functions (**Figure** **2**A). Co‐loading of ChOx further enhanced the catalytic cascade, amplifying ROS generation, depleting antioxidants, and reshaping the redox TME to favor ferroptosis and immune activation. Cholesterol depletion was evaluated by incubating cholesterol with PBS, FC (SiO_2_@Fe/CN–CS), FMCC, or free ChOx, followed by quantification with a commercial assay. Both FMCC and free ChOx significantly reduced cholesterol levels compared to PBS and FC (Figure 2B), confirming the enzymatic activity of FMCC. CAT‐like function was demonstrated by O_2_ evolution during H_2_O_2_ decomposition (Figure 2C), with higher activity at mildly acidic pH (6.5), consistent with the TME, and providing oxygen to support ChOx‐mediated cholesterol oxidation. GSH depletion was assessed by 5,5′‐Dithiobis (2‐nitrobenzoic acid) (DTNB) assay, showing that FMCC (800 µg mL^−1^) consumed 91.3% of GSH, far exceeding the ChOx‐free FC group (Figure 2D,E). This potent antioxidant depletion highlights FMCC's role in oxidative stress amplification and ferroptosis induction.

In POD activity assays, FMCC (100 µg mL^−1^) exhibited rapid catalytic efficiency in the presence of 3,3′,5,5′‐Tetramethylbenzidine (TMB), H_2_O_2_, and cholesterol, elevating the absorbance at 652 nm to 0.677 within 1 min, markedly higher than FC and other controls (Figure 2F; Figure S4, Supporting Information), confirming superior POD‐like activity. To further evaluate its catalytic kinetics and stability, absorbance at 652 nm was monitored using varying concentrations of TMB and H_2_O_2_ (Figure 2H,I), followed by Michaelis–Menten kinetic analysis (Figure 2J,K). With TMB fixed at 0.8 mm and H_2_O_2_ as the variable substrate, the Michaelis–Menten constant (*K*_M_) and maximum reaction velocity (V_max_) were determined to be 204 µm and 1030 nm·s^−1^, respectively. Conversely, with H_2_O_2_ fixed at 500 µm and TMB varied, the *K*_M_ and V_max_ values were 206.5 µm and 85.77 nm·s^−1^, respectively. Indicating favorable catalytic efficiency and substrate affinity.

Moreover, •OH generation under mildly acidic conditions was evaluated using 5,5‐dimethyl‐1‐pyrroline‐N‐oxide (DMPO) as a spin‐trapping agent. A strong Electron Spin Resonance (ESR) signal was observed at pH 6.5 upon incubation with 100 µg FMCC (Figure 2G), confirming effective ROS production in a tumor‐relevant acidic microenvironment.

### 2.2 Preparation and Characterization of Thermo‐Responsive Hydrogel PNH and Nanozyme‐Hybrid Hydrogel FMCC@PNH

**Figure 3:**
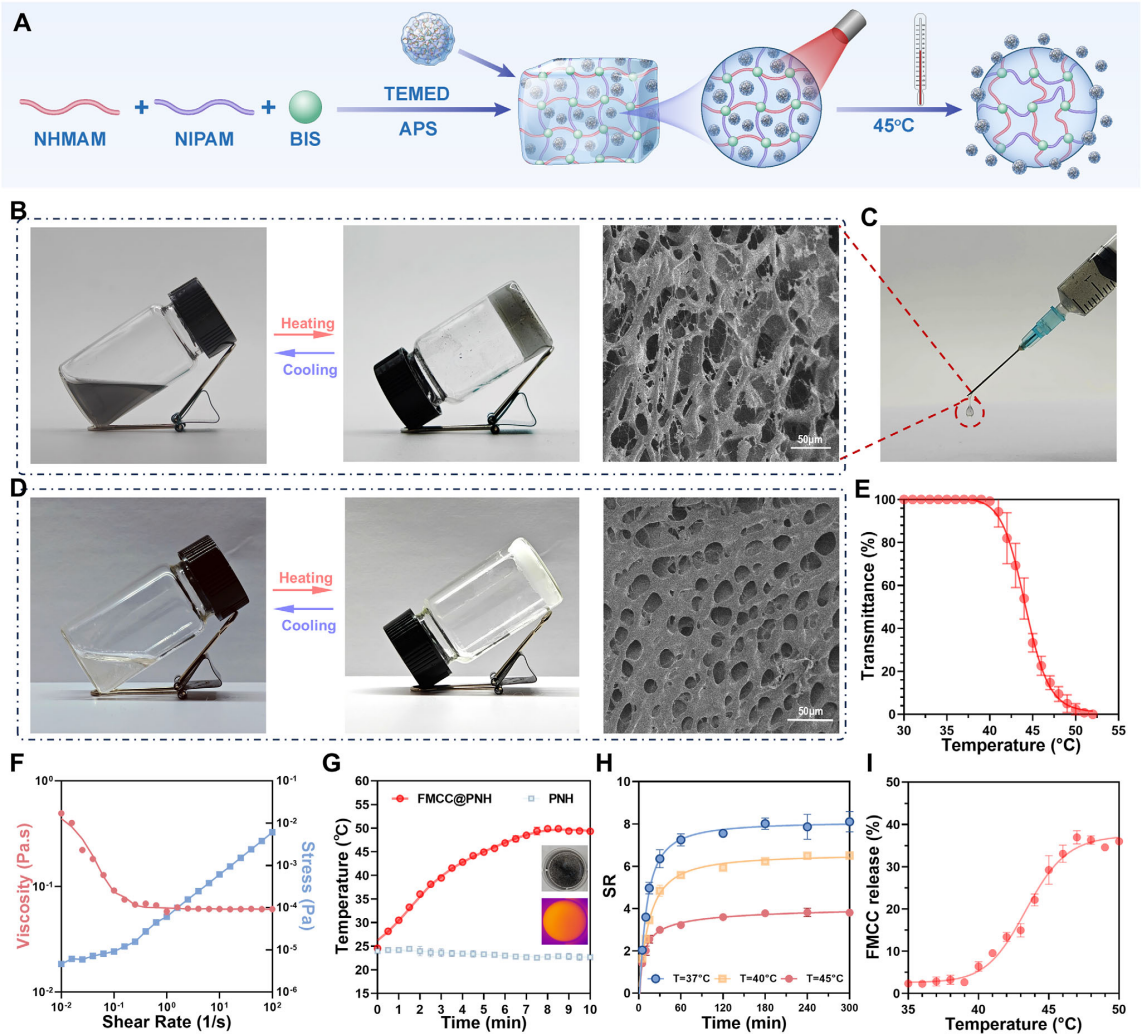
Fabrication and physicochemical characterization of FMCC@PNH. A) Fabrication process of FMCC@PNH: NIPAM and NHMAM were copolymerized to form the PNH hydrogel, which was physically blended with FMCC to obtain FMCC@PNH. Upon NIR irradiation, the PNH undergoes a phase transition to trigger controlled release. B,D) Photographs of FMCC@PNH (B) and PNH (D) at room temperature and after heating, demonstrating thermally induced transparency changes. Insets: corresponding SEM images showing porous (PNH) and dense (FMCC@PNH) microstructures. C) Representative image showing injectable FMCC@PNH droplets extruded from a syringe, highlighting its injectability. E) Temperature‐dependent light transmittance of FMCC@PNH (*n* = 3), confirming its LCST behavior. F) Rheological analysis showing shear‐thinning behavior: viscosity (red) decreases and shear stress (blue) increases with increasing shear rate. G) Photothermal performance of FMCC@PNH and PNH under 808 nm NIR laser irradiation (*n* = 3). Inset: infrared thermal image of FMCC@PNH during irradiation. H) Swelling behavior of FMCC@PNH at various temperatures (*n* = 3), showing maximal expansion below LCST. I) Cumulative FMCC release from FMCC@PNH at different temperatures (*n* = 3), demonstrating temperature‐triggered release above 43 °C.

The thermo‐responsive hydrogel poly(N‐isopropylacrylamide) (PNIPAM) is a widely studied smart material that undergoes a reversible phase transition near its LCST, typically ≈33 °C.^[^ ^35^ ^]^ Above this temperature, weakened hydrogen bonding and enhanced hydrophobic interactions induce polymer collapse and water expulsion, enabling rapid drug release. Copolymerization with hydrophilic NHMAM modulates the LCST, as its structural similarity to NIPAM promotes efficient network integration. As shown in **Figure** **3**A, the PNIPAM‐co‐NHMAM copolymer hydrogel was synthesized via free‐radical polymerization under low‐temperature, oxygen‐free conditions. NIPAM and NHMAM were dissolved in aqueous solution with N, N’‐methylenebisacrylamide (BIS) as a crosslinker and ammonium persulfate (APS) as an initiator, catalyzed by tetramethylethylenediamine (TEMED). The FMCC nanozyme was subsequently incorporated into the hydrogel matrix via physical entrapment.

By varying the NIPAM/NHMAM ratio and adjusting crosslinker/initiator levels (Tables S1 and S2, Supporting Information), we established an injectable hydrogel with an LCST aligned to the photothermal profile of FMCC under 808 nm irradiation. A formulation containing 85 wt.% NHMAM (LCST ≈45 °C) was selected as the final carrier (PNH, Figure S5, Supporting Information). FMCC was incorporated into the hydrogel matrix at varying mass ratios, and photothermal heating curves under NIR irradiation were recorded to assess the temperature responsiveness of the composites (Figures S6 and S7, Supporting Information). The cytocompatibility of the hydrogel formulations was then examined in human embryonic kidney (HEK293) cells without laser exposure, while photothermal cytotoxicity was evaluated in MB49 bladder cancer cells under NIR irradiation using CCK‐8 assays (Figure S8, Supporting Information). Based on these results, an FMCC loading concentration of 0.4 wt.% was selected for subsequent experiments.

FMCC@PNH retained the sharp LCST transition of PNH (Figure 3E) and showed pronounced shear‐thinning behavior (Figure 3F), characterized by a rapid decrease in viscosity and a gradual increase in shear stress with increasing shear rate, indicative of excellent injectability. As shown in Figure 3C, FMCC@PNH could be smoothly extruded through a fine‐gauge needle into uniform gel droplets, further supporting its suitability for intravesical administration. Photographic comparisons of PNH and FMCC@PNH under ambient and elevated temperatures (Figure 3B,D) revealed a transition to increased transparency upon heating, attributed to volume shrinkage, which reversed upon cooling, demonstrating reversible thermal responsiveness. Scanning Electron Microscope (SEM) analysis of freeze‐dried hydrogels showed a well‐organized porous structure in PNH, favorable for drug loading and diffusion, while FMCC@PNH exhibited a denser and more heterogeneous morphology due to the incorporation of FMCC nanozymes. Under 808 nm NIR irradiation, FMCC@PNH rapidly heated to 48.5 °C within 7 min, whereas PNH alone showed negligible change (Figure 3G). At 37 °C, the hydrogel swelled ∼ninefold, retaining FMCC and limiting leakage. Above 43 °C, contraction triggered accelerated release (Figure 3H,I).

Together, these findings highlight the robust photothermal responsiveness, injectability, and stimuli‐sensitive release characteristics of FMCC@PNH, underscoring its potential as a NIR‐activated precision delivery platform for localized bladder cancer therapy.

### 2.3 FMCC@PNH Promotes Ferroptosis in Tumor Cells In Vitro

**Figure 4:**
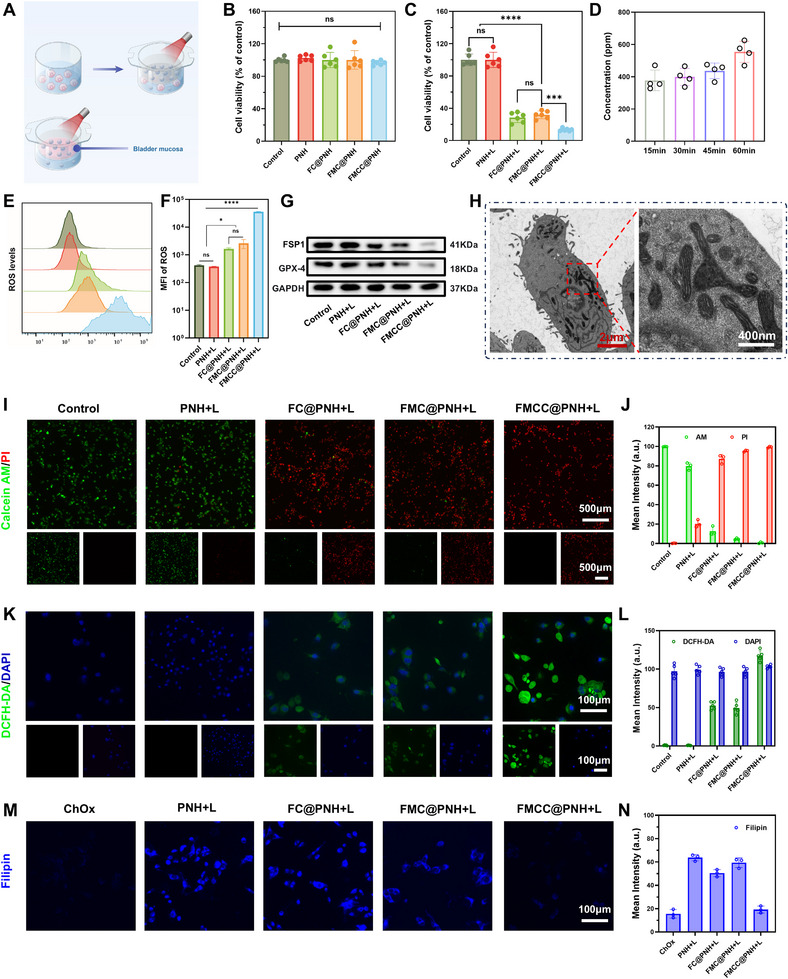
In vitro evaluation of FMCC@PNH enhanced tumor therapeutic efficacy. A) Schematic illustration of the in vitro experimental setup for FMCC release and NIR‐triggered trans‐urothelial penetration across bladder mucosa. B) Cell viability of HEK293 human embryonic kidney cells after 12 h treatment with Control, PNH, FC@PNH, FMC@PNH, and FMCC@PNH without NIR irradiation. C) Viability of MB49 murine bladder cancer cells after 12 h treatment with the same groups under 808 nm NIR irradiation, showing significant photothermal‐enhanced cytotoxicity. D) Quantification of FMCC concentration in the lower chamber during the mucosal penetration assay. E,F) Flow cytometry histogram (E) and quantification of mean fluorescence intensity (F) of intracellular ROS (DCFH‐DA) levels. G) Western blot analysis of ferroptosis‐related proteins GPX4 and FSP1 following various treatments. H) TEM images of MB49 cells showing mitochondrial ultrastructural changes indicative of ferroptosis upon FMCC@PNH + L treatment. I,J) Live/dead staining using Calcein‐AM (green) and PI (red) (I), and corresponding quantification of fluorescence intensity (J). K,L) ROS generation visualized by DCFH‐DA staining (K) and its quantification (L) in MB49 cells. M,N) Filipin staining (M) and fluorescence quantification (N) of free cholesterol in MB49 cells, indicating ChOx‐mediated cholesterol degradation. The data are presented as the means ± SDs and were analyzed by one‐way two‐sided analysis of variance (ANOVA) with GraphPad Prism software. ^*^ *p* <0.05, ^**^ *p* <0.01, ^***^ *p* <0.001, ^****^ *p* <0.0001, “ns” indicates no statistical significance.

To systematically evaluate the in vitro tumoricidal efficacy of FMCC@PNH, an 8 µm pore‐sized Transwell system was employed for hydrogel–cell co‐culture (**Figure** **4**A). Initially, the biocompatibility of FMCC@PNH (FeMn@CN‐ChOx‐CS@PNH), its precursors FC@PNH (SiO_2_@Fe/CN‐CS@PNH) and FMC@PNH (FeMn@CN‐CS@PNH), pure PNH hydrogel, and PBS control was assessed using human embryonic kidney cells (HEK293) without NIR irradiation. CCK‐8 assays revealed no significant cytotoxicity across all groups, confirming the favorable biocompatibility of the hydrogel formulations (Figure 4B). Subsequently, the antitumor efficacy was assessed by incubating the materials with MB49 murine bladder cancer cells, followed by 5 min of 808 nm NIR laser irradiation. Cell viability assays demonstrated that FC@PNH, FMC@PNH, and FMCC@PNH significantly suppressed MB49 viability, with FMCC@PNH achieving the strongest effect, reducing viability to below 20%, indicative of potent photothermal‐enhanced cytotoxicity (Figure 4C). To investigate the trans‐urothelial delivery potential relevant to intravesical therapy, a murine bladder mucosal barrier model was established by mounting isolated bladder mucosa from healthy C57BL/6 mice onto the upper chamber of a Transwell insert (Figure 4A). Upon 10 min of NIR irradiation, FMCC concentration in the lower chamber was quantified via UV–vis spectroscopy at 325 nm based on a standard calibration curve (Figure S9, Supporting Information). The results confirmed effective NIR‐triggered tissue penetration by FMCC@PNH (Figure 4D), supporting its potential for controlled intravesical delivery. This finding was consistent with Confocal laser scanning microscopy (CLSM) imaging, which visualized the distribution of FITC‐labeled FMCC across bladder tissues following intravesical administration (Figure S10, Supporting Information). The penetration capacity of FMCC into tumor tissues was further validated using a 3D tumor spheroid model. FMCC solution alone was applied, followed by 5 min of NIR irradiation and 4 h of incubation. CLSM imaging revealed substantial intratumoral penetration (Figure S11, Supporting Information). To confirm cellular internalization, a fluorescence uptake assay was conducted. Cy5.5‐labeled FMCC (red) was observed within the cytoplasm, situated between the DiO‐labeled membrane (green) and DAPI‐stained nucleus (blue), indicating efficient cellular uptake (Figure S12, Supporting Information). To further evaluate cytotoxicity and elucidate the underlying cell death mechanism, Calcein‐AM/PI dual staining was performed. After 10 min of laser irradiation and 12 h of incubation, marked cell death was observed in all treatment groups, with FMCC@PNH + laser inducing the most extensive cytotoxic effect (Figure 4I,J).

To elucidate the underlying oxidative stress mechanism, intracellular ROS levels were assessed using the DCFH‐DA fluorescent probe (Figure 4K,L). Notably, FMCC@PNH + L treatment induced the highest ROS accumulation among all groups. This trend was consistent with flow cytometry results (Figure 4E,F), confirming enhanced ROS production upon treatment. The elevated ROS levels were attributed to the enzymatic oxidation of intracellular cholesterol by ChOx, which synergistically enhanced the POD‐like catalytic activity of the nanozyme. Filipin staining further validated cholesterol oxidation, revealing a significant decrease in free cholesterol fluorescence in the FMCC@PNH + L group (Figure 4M,N), indicating effective degradation of membrane cholesterol. Compared with ChOx‐free formulations, FMCC@PNH efficiently disrupted intracellular cholesterol metabolism, thereby enhancing catalytic reactivity and facilitating membrane remodeling and ferroptosis initiation. Supporting this, expression of the lipid raft marker CD59 was markedly downregulated following FMCC@PNH + L treatment (Figure S13, Supporting Information), reflecting cholesterol‐mediated membrane disintegration.

**Figure 5:**
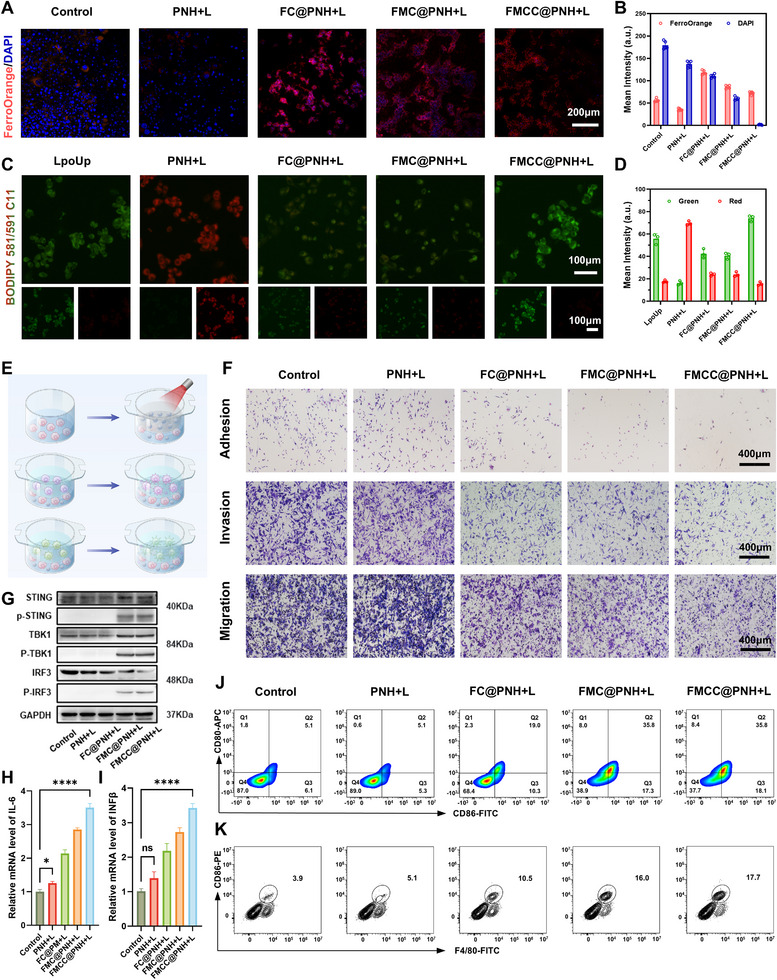
In vitro evaluation of FMCC. A,B) Fluorescence staining of Fe^2+^ (FerroOrange)/ cell nucleus (DAPI) (A) and quantification of mean fluorescence intensity (B). C,D) Lipid peroxidation detected using BODIPY 581/591 C11 probe (C), and quantification of oxidized (green)/reduced (red) signal ratio (D). E) Schematic of the in vitro co‐culture model for immune activation: MB49 pre‐treatment, followed by co‐culture with RAW264.7 macrophages and DCs for phenotype assessment. F) Representative images showing MB49 cell migration, adhesion, and invasion under different treatments. G) Western blot analysis of STING pathway‐related proteins (p‐STING, p‐TBK1, p‐IRF3). H,I) Relative mRNA expression of IL‐6 (H) and IFN‐β (I) measured by qRT‐PCR, reflecting STING‐mediated immune activation. J,K) Flow cytometry analysis of DC maturation (CD80^+^ CD86^+^, J) and macrophage polarization toward the M1 phenotype (CD86^+^ F4/80^+^, K) following FMCC@PNH + L treatment. The data are presented as the means ± SDs and were analyzed by one‐way two‐sided analysis of variance (ANOVA) with GraphPad Prism software. ^*^ *p* <0.05, ^**^ *p* <0.01, ^***^ *p* <0.001, ^****^ *p* <0.0001, “ns” indicates no statistical significance.

Ferroptosis induction was further confirmed by FerroOrange staining and quantitative fluorescence analysis (**Figure** **5**A,B), which showed significant Fe^2+^ accumulation in the FC@PNH + L, FMC@PNH + L, and FMCC@PNH + L groups. The relatively weaker Fe^2+^ signal in the FMC@PNH + L group may result from Mn^2+^ mediated Fe^3+^/Fe^2+^ redox cycling (Mn^2+^ + Fe^3+^ ⇌ Mn^3+^ + Fe^2+^), accelerating Fenton‐like reactions. Remarkably, the FMCC@PNH + L group exhibited a diminished overlap of FerroOrange and DAPI fluorescence, suggesting severe ferroptosis and apoptotic damage accompanied by Fe^2+^ leakage and nuclear condensation. Lipid peroxidation was evaluated using the BODIPY 581/591 C11 probe, which shifts fluorescence from red to green upon oxidation. As shown in Figure 5C,D, FMCC@PNH + L triggered the most pronounced lipid peroxidation, surpassing even the LpoUp positive control, further supporting its potent ferroptosis‐inducing capability.

Western blot analysis provided additional mechanistic insights. As shown in Figure 5G, expression levels of the two key ferroptosis regulators, GPX4 and FSP1, were significantly downregulated following FMCC@PNH + L treatment, while remaining relatively stable in the control and other treatment groups. GPX4, a glutathione‐dependent peroxidase, mitigates lipid peroxidation, whereas FSP1 functions independently by regenerating ubiquinol from CoQ_10_ to inhibit lipid oxidation. The FMCC nanozyme mimicked GSH oxidase activity, effectively depleting intracellular GSH and impairing GPX4 function, while also suppressing FSP1 expression. This dual inhibition effectively disrupts both major ferroptosis defense pathways, amplifying lipid peroxidation and cell death.^[^ ^21^ ^]^ To visualize the ultrastructural features of ferroptosis, TEM was performed. Compared to the control group, which exhibited intact mitochondria with dense cristae (Figure S14, Supporting Information), FMCC@PNH + L‐treated cells displayed classical ferroptosis morphology, including mitochondrial shrinkage, cristae loss, increased membrane density, and vacuole‐like deformities, hallmarks of severe mitochondrial injury (Figure 4H). These results demonstrate that FMCC@PNH induces robust ferroptosis through synergistic ROS generation, lipid peroxidation, and membrane destabilization, providing a promising strategy for effective tumor eradication.

### 2.4 FMCC@PNH Activates the STING Pathway and Stimulates Immune Cell Activation

To assess the capacity of FMCC@PNH to activate innate immune pathways, an in vitro immune activation model was established using a Transwell‐based co‐culture system (Figure 5E). Briefly, MB49 bladder cancer cells were seeded in an 8 µm pore‐size Transwell insert and co‐incubated with the hydrogel, followed by 808 nm NIR laser irradiation for 5 min and a 12 h incubation. The insert was then replaced with a 0.4 µm pore‐size chamber, and RAW264.7 macrophages were added to the upper compartment for an additional 24 h co‐culture. DCs were subjected to the same protocol. This setup mimics the post‐irradiation tumor–immune cell crosstalk mediated by the nanozyme–hydrogel system.

Western blot analysis (Figure 5G) was performed to examine activation of the cGAS–STING pathway. No significant upregulation of phosphorylated STING (p‐STING), TBK1 (p‐TBK1), or IRF3 (p‐IRF3) was observed in the PBS control, PNH + L, or FC@PNH + L groups, indicating minimal pathway activation. In contrast, the FMC@PNH + L and FMCC@PNH + L groups, both containing Mn^2+^, exhibited marked increases in p‐STING, p‐TBK1, and p‐IRF3 levels upon irradiation, confirming Mn^2+^ as a critical STING agonist within this system. To further validate STING pathway activation, the expression of downstream cytokines was assessed by qRT‐PCR. As shown in Figure 5H,I, FMCC@PNH + L treatment significantly upregulated IL‐6 and IFN‐β mRNA levels, suggesting robust induction of type I interferon responses and pro‐inflammatory signaling, both essential for enhancing tumor immunogenicity.

To determine whether this innate immune activation was accompanied by functional immune remodeling, DC maturation and macrophage polarization were evaluated. Flow cytometry analysis (Figure 5J) revealed increased expression of co‐stimulatory molecules CD80 and CD86 on DCs after FMCC@PNH + L treatment, indicative of effective DC maturation and potential promotion of T cell‐mediated adaptive immunity. Moreover, macrophage phenotyping (Figure 5K) demonstrated a significant increase in the proportion of M1‐like (CD86^+^ F4/80^+^) pro‐inflammatory macrophages, supporting enhanced polarization toward an anti‐tumor phenotype, further supporting the immune‐potentiating effect of FMCC@PNH.

### 2.5 Suppression of Tumor Cell Metastasis by FMCC@PNH Hydrogel

To evaluate the impact of different treatments on the metastatic behavior of tumor cells, a Transwell assay was employed to assess the adhesion, invasion, and migration of MB49 bladder cancer cells (Figure 5F), showing a trend consistent with the wound healing assay (Figure S15, Supporting Information). In the control and PNH + L groups, extensive cell transmigration was observed, indicating that hydrogel alone with laser irradiation was insufficient to effectively restrict tumor cell motility. In contrast, the FC@PNH + L group exhibited moderate inhibition of cell migration and adhesion, attributed to the POD‐like activity of the nanozyme, which catalyzed ROS generation under NIR irradiation, inducing oxidative and photothermal damage.

More pronounced suppression was observed in the FMC@PNH + L and FMCC@PNH + L groups, with significantly reduced numbers of transmigrated cells. This enhanced effect was ascribed not only to the elevated ROS levels mediated by nanozyme catalysis but also to the Mn^2+^‐induced activation of the STING pathway. As a known STING agonist, Mn^2+^ stimulates the expression of immunomodulatory cytokines such as IFN‐β and IL‐6, which impede epithelial–mesenchymal transition (EMT), suppress matrix metalloproteinases (MMPs), and disrupt cytoskeletal dynamics, jointly impairing the invasive and migratory capacities of tumor cells.^[^ ^31^ ^]^ Notably, the FMCC@PNH + L group exhibited the most significant anti‐metastatic effect. Beyond ROS elevation and immune activation, this effect was further attributed to the action of ChOx, which catalyzes cholesterol oxidation and disrupts cholesterol‐rich lipid rafts, key platforms for cell adhesion, polarity, and migration signaling. The collapse of these structures interferes with the recruitment of migration‐related molecules and their downstream signaling cascades, fundamentally impairing cancer cell motility.^[^ ^23^ ^]^

Collectively, these results underscore the potent anti‐metastatic potential of FMCC@PNH, a multi‐mechanistic nano‐hydrogel platform, offering a promising strategy for preventing tumor recurrence and metastasis.

### 2.6 In Vivo Biodegradation and Biosafety Evaluation of FMCC@PNH

To systematically assess the in vivo safety and biodegradation of FMCC@PNH for intravesical application, a tenfold therapeutic dose of the hydrogel was subcutaneously administered to healthy C57BL/6 mice. At predetermined intervals, residual gels were harvested and lyophilized for dry weight analysis. As shown in Figure S16 (Supporting Information), FMCC@PNH exhibited gradual degradation over time and was almost completely resorbed by day 28, indicating excellent biodegradability and tissue compatibility.

For biosafety evaluation, hematological and serum biochemical parameters were measured and compared with PBS‐treated controls. As shown in Figure S17 (Supporting Information), key indices, including WBC, PLT, AST, ALT, BUN, and CREA, remained within normal physiological ranges, suggesting negligible systemic toxicity. Histological analysis of major organs (heart, liver, lungs, spleen, and kidneys) via H&E staining showed no pathological alterations or inflammatory infiltration, further confirming the high biocompatibility of the hydrogel (Figure S18, Supporting Information).

### 2.7 Antitumor Efficacy of FMCC@PNH in a Metastatic Bladder Cancer Model

**Figure 6:**
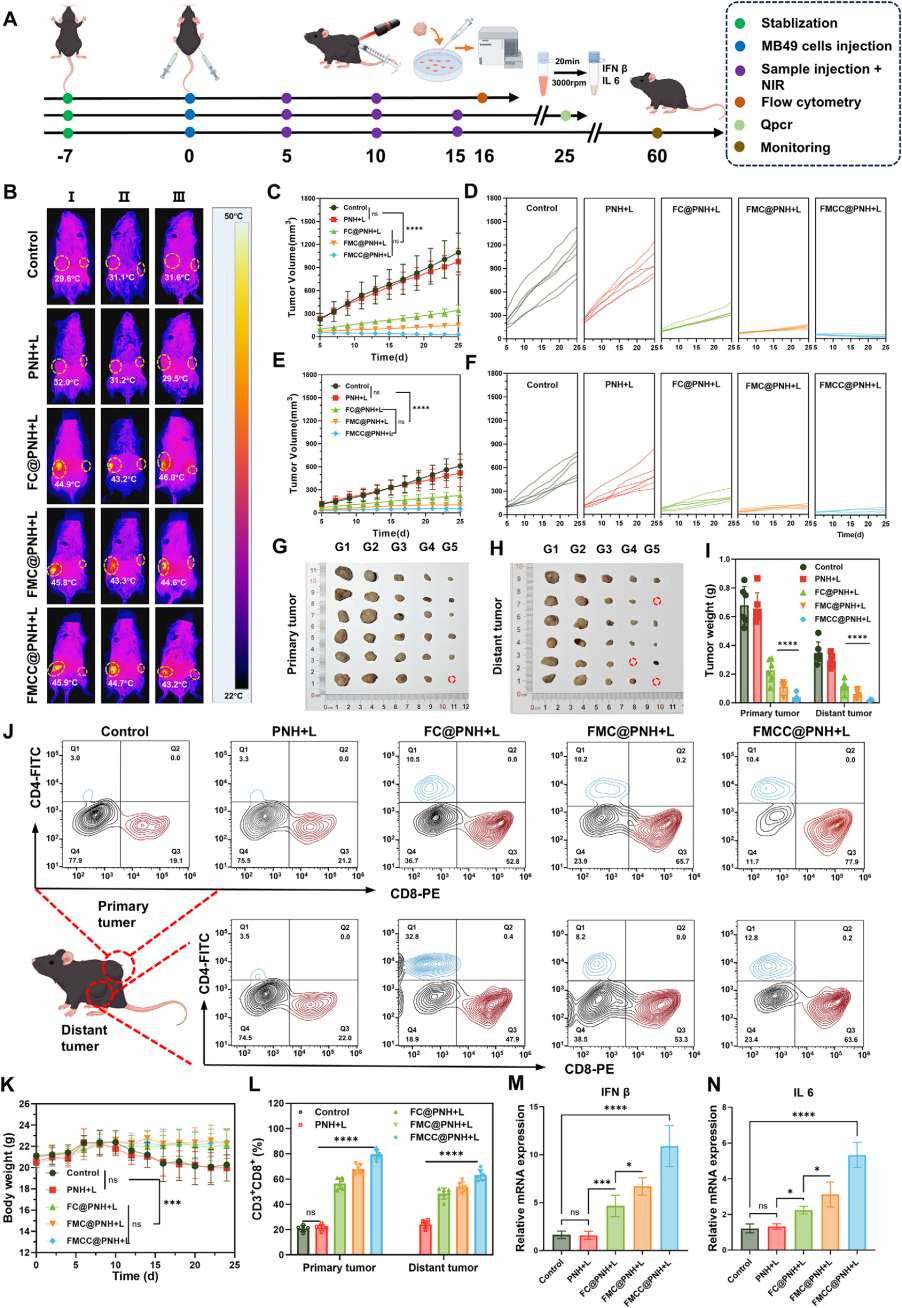
In vivo evaluation of antitumor activity in a bilateral subcutaneous tumor model. A) Schematic illustration of the treatment procedures. B) Thermal images of tumor‐bearing mice injected with FMCC@PNH under 808 nm laser irradiation (1.0 W cm^−2^). C,D) Total (C) and individual (D) growth curves of primary tumor volumes in mice receiving different treatments. E,F) Total (E) and individual (F) growth curves of distant tumor volumes. G,H) Representative digital images of excised primary (G) and distant (H) tumors in different treatment groups. G1: Control, G2: PNH + Laser, G3: FC@PNH + Laser, G4: FMC@PNH + Laser, G5: FMCC@PNH + Laser. I) Tumor weights of both primary and distant tumors in each group. J) Representative flow cytometry contour plots of infiltrating CD3^+^ CD4^+^ and CD3^+^ CD8^+^ T cells in primary tumors (top) and distant tumors (bottom). K) Body weight changes of tumor‐bearing mice during the treatment period. L) Quantification of intratumoral CD8^+^ T cell infiltration. M,N) Relative mRNA expression levels of IFN‐β (M) and IL‐6 (N) in tumor tissue. The data are presented as the means ± SDs and were analyzed by one‐way two‐sided analysis of variance (ANOVA) with GraphPad Prism software. ^*^ *p* <0.05, ^**^ *p* <0.01, ^***^ *p* <0.001, ^****^ *p* <0.0001, “ns” indicates no statistical significance.

To comprehensively evaluate the antitumor efficacy of FMCC@PNH in metastatic bladder cancer, a bilateral subcutaneous MB49 tumor model was established in C57BL/6 mice. A low dose of tumor cells was injected into the left flank to mimic a distant metastatic lesion, while the right side served as the primary tumor site. Mice were randomly divided into five groups: G1: Control, G2: PNH + L, G3: FC@PNH + L, G4: FMC@PNH + L, G5: FMCC@PNH + L. Starting from day 5, only the primary tumor region received peritumoral subcutaneous injections and NIR laser irradiation, while the distant tumor was left untreated (**Figure** **6**A). Infrared thermal imaging (Figure 6B) confirmed that all nanoplatforms produced effective photothermal responses under laser irradiation, consistent with in vitro results.

Throughout the treatment period, tumor volumes on both sides and body weights were closely monitored. Group G5 exhibited the most significant inhibition of both primary and distant tumor growth, with the smallest tumor volumes and weights among all groups (Figure 6C,E,G–I). Notably, suppression of the distant tumor was especially pronounced in this group. Individual tumor growth curves (Figure 6D,F) demonstrated consistent and sustained therapeutic efficacy. Body weight tracking (Figure 6K) revealed noticeable weight loss in G1 and G2, while G3–G5 maintained stable weights, indicating favorable systemic tolerability. Survival analysis (Figure S19, Supporting Information) further supported the biosafety and strong antitumor efficacy of the FMCC@PNH system.

To investigate the immune mechanisms underlying tumor suppression, we assessed immune cell infiltration and cytokine expression in both primary and distant tumors. Flow cytometry (Figure 6J,L) revealed significantly increased infiltration of CD8^+^ cytotoxic T lymphocytes in the G5 group, 79.5% in primary tumors and 64.2% in distant lesions, exceeding all other groups. This suggests robust activation of both local and systemic T cell‐mediated immune responses. The enhanced immune response is primarily attributed to the ChOx component of FMCC, which depletes excess cholesterol in the TME, a known factor in CD8^+^ T cell exhaustion. Evidence indicates that cholesterol overload in tumors induces CD8^+^ T cell dysfunction through endoplasmic reticulum (ER) stress and activation of the XBP1 pathway.^[^ ^25^ ^]^ Therefore, cholesterol depletion effectively restores CD8^+^ T cell function. ChOx‐driven metabolic reprogramming not only alleviates T cell exhaustion but also enhances CTL‐mediated antitumor immunity.^[^ ^26^, ^27^ ^]^ Moreover, qPCR analysis (Figure 6M,N) showed upregulated expression of IFN‐β and IL‐6 mRNA in all treatment groups, with the greatest elevation in G5. This is likely due to Mn^2+^‐mediated STING pathway activation, which promotes type I interferon and pro‐inflammatory cytokine production, further enhancing immune cell recruitment and systemic antitumor activity.

### 2.8 Antitumor Efficacy of FMCC@PNH in a Post‐Surgical Recurrent Bladder Cancer Model

**Figure 7:**
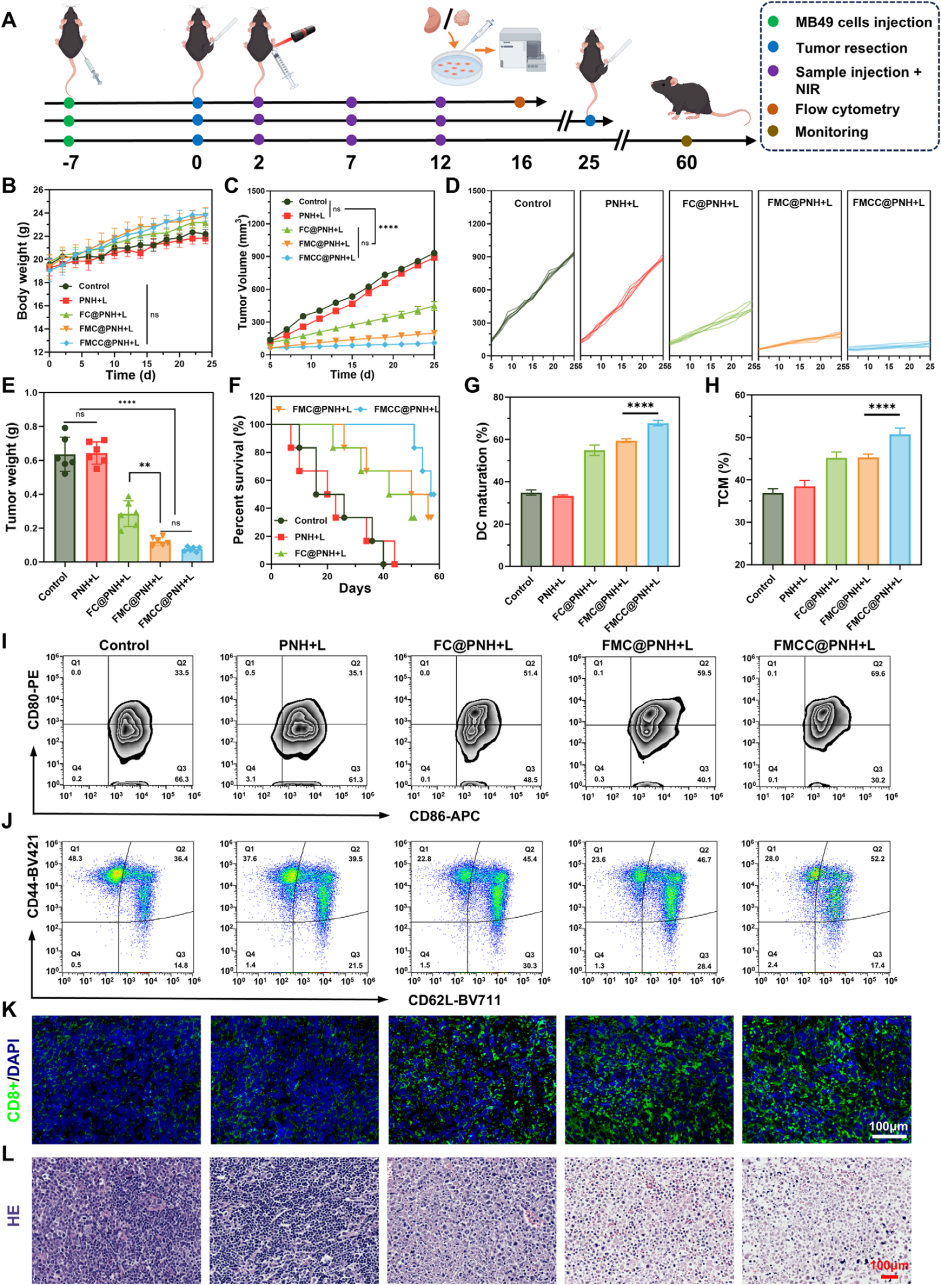
In vivo evaluation of antitumor activity in a recurrent tumor model. A) Schematic illustration of the treatment procedures. B) Body weight changes of tumor‐bearing mice under different treatments. C,D) Total (C) and individual (D) tumor volume curves. E) Tumor weights in each treatment group. F) Survival curves of mice in each group. G) Proportion of TCM cells in the spleens of treated mice. H) Proportion of mature DCs in tumor tissues. I) Representative flow cytometry plots of mature DCs (gated on CD11c^+^ CD80^+^ CD86^+^) in tumor tissues. J) Representative flow cytometry plots showing TCM cells (gated on CD44^+^ CD62L^+^) in the spleen. K) CLSM images of CD8^+^ T cells immunofluorescence staining. L) Representative H&E‐stained images of tumor sections from each group. The data are presented as the means ± SDs and were analyzed by one‐way two‐sided analysis of variance (ANOVA) with GraphPad Prism software. ^*^ *p* <0.05, ^**^ *p* <0.01, ^***^ *p* <0.001, ^****^ *p* <0.0001, “ns” indicates no statistical significance.

Bladder cancer's high recurrence rate remains a major clinical challenge that compromises long‐term patient survival.^[^ ^1^ ^]^ To evaluate the potential of the FMCC@PNH hydrogel system in preventing tumor recurrence post‐surgery, we established a murine model of incomplete tumor resection. Subcutaneous tumors were induced by MB49 cell injection seven days prior to treatment. On day 0, mice underwent partial tumor resection and were randomly divided into five groups as previously described. Hydrogel administration and NIR laser irradiation were performed on days 2, 7, and 12. Throughout the treatment, mouse body weights and tumor regrowth were monitored. As shown in **Figure** **7**B–F, body weights remained stable across all groups (Figure 7B), indicating good biocompatibility. Group G5 significantly delayed tumor regrowth (Figure 7C,D), resulting in the smallest final tumor volumes (Figure 7E) and longest survival (Figure 7F).

Immunofluorescence staining (Figure 7K) revealed minimal CD8^+^ T cell infiltration in G1 and G2, while G3 showed moderate infiltration. G4 and G5 demonstrated widespread and intense CD8^+^ T cell presence, indicating robust cytotoxic T cell recruitment. These findings were supported by H&E staining (Figure 7L), where G1 and G2 retained intact tumor architecture. G3 induced focal necrosis, G4 showed extensive destruction, and G5 had the fewest residual tumor cells. Flow cytometry of tumor‐infiltrating DCs revealed that mature DCs (CD80^+^ CD86^+^) were highest in G5 (67.7%) compared to G1 (34.9%) and G2 (33.2%) (Figure 7G,I). This suggests that photothermal‐induced immunogenic cell death promotes antigen release and DC maturation. Analysis of splenic T cell subsets showed that G5 had a significant increase in TCM cells, (CD44^+^ CD62L^+^), reaching 40.3% (Figure 7H,J). This enhancement likely results from Mn^2^⁺‐mediated STING activation and ChOx‐driven cholesterol depletion, which promote type I interferon release and relieve CD8^+^ T cell exhaustion, ultimately establishing durable immune memory.^[^ ^31^ ^]^

Collectively, FMCC@PNH combined with PTT not only suppresses postoperative recurrence but also elicits systemic immune responses by enhancing DC maturation, CD8^+^ T cells infiltration, and TCM cells formation, offering a promising strategy for relapse and metastasis prevention.

### 2.9 Antitumor Efficacy of FMCC@PNH in an Orthotopic Bladder Cancer Model

**Figure 8:**
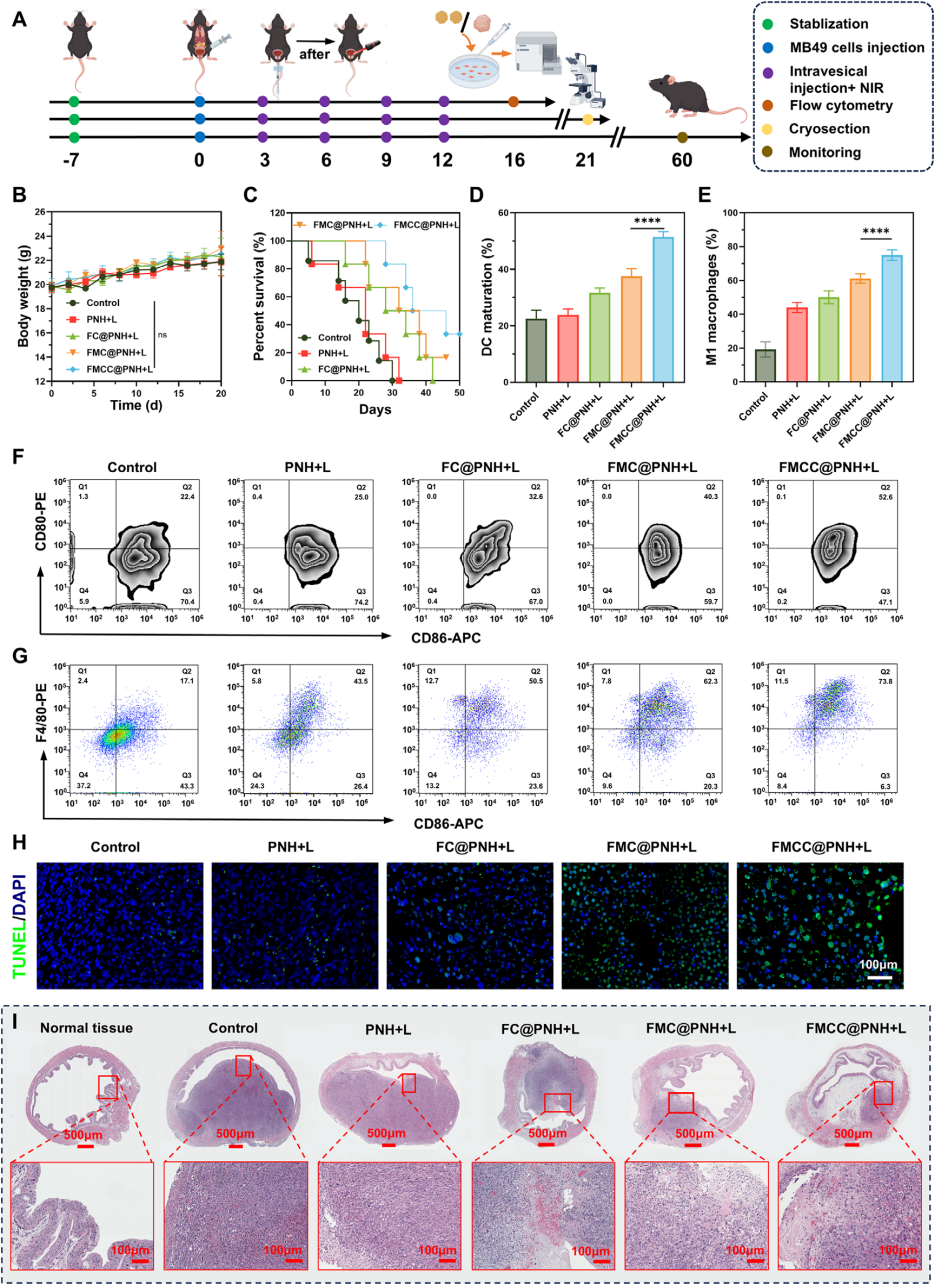
In vivo evaluation of therapeutic efficacy in an orthotopic bladder tumor model. A) Schematic illustration of the treatment procedures. B) Body weight changes of tumor‐bearing mice receiving different treatments. C) Survival curves of mice in each treatment group. D) Proportion of mature DCs in inguinal lymph nodes. E) Quantification of M1‐type macrophages in tumor tissues. F) Representative flow cytometric plots of mature DCs (gated on CD11c^+^ CD80^+^ CD86^+^) in lymph nodes. G) Representative flow cytometry plots showing M1‐type macrophages (CD86^+^ F4/80^+^) in lymph nodes. H) Representative CLSM images of TUNEL (green) and DAPI (blue) co‐staining in tumor sections. I) Representative H&E‐stained cross‐sections of orthotopic bladder tumors. The data are presented as the means ± SDs and were analyzed by one‐way two‐sided analysis of variance (ANOVA) with GraphPad Prism software. ^*^ *p* <0.05, ^**^ *p* <0.01, ^***^ *p* <0.001, ^****^ *p* <0.0001, “ns” indicates no statistical significance.

To simulate clinical bladder cancer treatment, an orthotopic bladder tumor model was developed. Following a lower abdominal incision under anesthesia, MB49 cells were directly injected into the bladder wall (Figure S20A, Supporting Information). Mice received intravesical hydrogel administration combined with NIR laser irradiation on days 3, 6, 9, and 12, using the same treatment groups. Ultrasound imaging monitored tumor progression (Figure S21, Supporting Information), while body weights were recorded to evaluate systemic safety (**Figure** **8**A). No significant body weight changes were observed (Figure 8B), indicating good biocompatibility. Tumor volumes in G3–G5 were significantly reduced compared to controls (Figure S22, Supporting Information), and infrared thermal imaging confirmed effective photothermal conversion across all formulations (Figure S20B, Supporting Information). Survival analysis demonstrated prolonged survival in G5 (Figure 8C). Urinalysis showed that G3–G5 effectively alleviated hematuria, indicating urothelial protection (Figure S23, Supporting Information).

To assess immune activation, bilateral inguinal lymph nodes were analyzed by flow cytometry. The proportion of mature DCs (CD80^+^ CD86^+^) increased from 22.5% (G1) and 23.8% (G2) to 31.7% (G3), 37.6% (G4), and peaked at 51.5% in G5 (Figure 8D,F), indicating enhanced antigen presentation and systemic immunity. Flow cytometry also revealed a shift in TAMs toward the pro‐inflammatory M1 phenotype (F4/80^+^ CD86^+^), especially in G5 (Figure 8E,G). M1 macrophages, known for secreting pro‐inflammatory cytokines and tumoricidal activity, reflect improved local immune conditions conducive to antitumor responses. Histological analysis of bladder tissue (Figure 8I) revealed intact tumor architecture in G1 and G2, while G3 showed partial necrosis and immune infiltration. G4 and G5 exhibited widespread necrosis, nuclear pyknosis, and dense immune cell infiltration, indicating strong local immune activation. TUNEL staining (Figure 8H) confirmed that G5 induced the highest level of apoptosis, consistent with enhanced DC maturation and M1 polarization. These findings collectively demonstrate that FMCC@PNH hydrogel remodels the tumor immune microenvironment and activates both innate and adaptive immune responses, leading to synergistic antitumor effects.

## 3 Conclusion

In this study, we developed a multifunctional therapeutic platform, FMCC@PNH, by ingeniously integrating the phase‐transition‐controlled release capability of a thermo‐responsive hydrogel (PNH) with the photothermal properties of a bimetallic nanozyme (FMCC). Upon intravesical administration, this hybrid hydrogel enabled localized and precise drug delivery for bladder cancer. Under near‐infrared (NIR) laser irradiation, the FMCC‐mediated photothermal effect triggered a rapid sol–gel phase transition in the PNH matrix, thereby promoting on‐demand release of catalytically active FMCC. The released FMCC exhibited potent multienzyme‐mimetic activities, including peroxidase (POD), catalase (CAT), and glutathione oxidase (GSHox)‐like functions, while the incorporation of natural cholesterol oxidase (ChOx) further enhanced its catalytic network. This synergistically induced robust reactive oxygen species (ROS) generation, which in turn triggered ferroptosis‐mediated tumor cell death. Importantly, ferroptosis not only eliminated tumor cells but also facilitated the release of damage‐associated molecular patterns (DAMPs) and immunostimulatory signals, thereby providing a mechanistic bridge to activate the STING pathway and initiate downstream antitumor immunity. Consequently, FMCC@PNH depleted intratumoral cholesterol and simultaneously amplified innate immune sensing, ultimately eliciting potent systemic immune responses.

Mechanistically, FMCC@PNH significantly promoted CD8^+^ T cell infiltration into tumor tissues, increased the proportion of mature dendritic cells (CD80^+^ CD86^+^) in draining lymph nodes, and facilitated M1‐like polarization of tumor‐associated macrophages, collectively reshaping the immunosuppressive tumor microenvironment (TME). A series of in vitro and in vivo experiments confirmed the excellent biosafety and therapeutic efficacy of FMCC@PNH, demonstrating its capability to inhibit tumor growth, metastasis, and recurrence.

In summary, FMCC@PNH represents a light‐responsive nanoplatform that integrates photothermal therapy (PTT), chemodynamic therapy, ferroptosis induction, and immune activation. By linking ferroptosis‐induced oxidative stress with immune pathway activation, this strategy not only achieves efficient tumor eradication but also reshapes the TME toward an immunostimulatory phenotype. This work provides a promising avenue for postoperative intravesical immunotherapy of bladder cancer and offers strong potential for clinical translation in precision oncology.

## Conflict of Interest

The authors declare no conflict of interest.

## Supporting information



Supporting Information

## Data Availability

The data that support the findings of this study are available from the corresponding author upon reasonable request.

## References

[1] L. Dyrskjot , D. E. Hansel , J. A. Efstathiou , M. A. Knowles , M. D. Galsky , J. Teoh , D. Theodorescu , Nat. Rev. Dis. Primers 2023, 9, 58. 37884563 10.1038/s41572-023-00468-9 PMC11218610

[2] H. Sung , J. Ferlay , R. L. Siegel , M. Laversanne , I. Soerjomataram , A. Jemal , F. Bray , Ca‐Cancer J. Clin. 2021, 71, 209. 33538338 10.3322/caac.21660

[3] M. Babjuk , M. Burger , E. M. Comperat , P. Gontero , A. H. Mostafid , J. Palou , B. W. G. van Rhijn , M. Roupret , S. F. Shariat , R. Sylvester , R. Zigeuner , O. Capoun , D. Cohen , J. L. Dominguez Escrig , V. Hernandez , B. Peyronnet , T. Seisen , V. Soukup , Eur. Urol. 2019, 76, 639. 31443960 10.1016/j.eururo.2019.08.016

[4] N. V. Jafari , J. L. Rohn , Mucosal Immunol. 2022, 15, 1127. 36180582 10.1038/s41385-022-00565-0 PMC9705259

[5] A. T. Lenis , P. M. Lec , K. Chamie , J. Am. Med. Assoc. 2020, 324, 1980.

[6] J. Zhu , Y. Zhu , J. Huang , W. Zhang , J. Hou , B. Z. Tang , D. Wang , Adv. Mater. 2025, 37, 2502452. 10.1002/adma.202502452 40345972

[7] R. Railkar , M. R. Siddiqui , T. Sanford , H. Kobayashi , P. L. Choyke , P. K. Agarwal , Clin. Cancer Res. 2020, 26, A09.

[8] T. Mao , H. Zhang , J. Cui , Z. Zhao , D. Jiao , W. Zhang , World J. Surg. Oncol. 2022, 20, 324. 36175920 10.1186/s12957-022-02786-w PMC9520848

[9] X. A. Jia , J. Wang , E. R. Wang , Adv. Mater. 2024, 36, 2309261. 10.1002/adma.202309261 38016341

[10] S. H. Qin , H. Y. Zhao , X. Y. Luo , F. Wang , J. Liu , Y. Ding , Y. Hu , ACS Nano 2024, 18, 32235. 39499796 10.1021/acsnano.4c13087

[11] H. Y. Yoon , H. M. Yang , C. H. Kim , Y. T. Goo , M. J. Kang , S. Lee , Y. W. Choi , Expert Opin. Drug Deliv. 2020, 17, 1555. 32791923 10.1080/17425247.2020.1810016

[12] G. Li , Q. Lei , F. Wang , D. Deng , S. Wang , L. Tian , W. Shen , Y. Cheng , Z. Liu , S. Wu , Small 2019, 15, 1900936. 10.1002/smll.201900936 31074941

[13] B. Zheng , D. Liu , X. Qin , D. Zhang , P. Zhang , Int. J. Nanomed. 2025, 20, 2241. 10.2147/IJN.S505427 PMC11849417 39995958

[14] L. M. Ensign , R. Cone , J. Hanes , J. Controlled Release 2014, 190, 500. 10.1016/j.jconrel.2014.04.033 PMC4142075 24830303

[15] U. Prabhakar , H. Maeda , R. K. Jain , E. M. Sevick‐Muraca , W. Zamboni , O. C. Farokhzad , S. T. Barry , A. Gabizon , P. Grodzinski , D. C. Blakey , Cancer Res. 2013, 73, 2412. 23423979 10.1158/0008-5472.CAN-12-4561 PMC3916009

[16] Y. Ye , Y. Liu , S. Ma , X. Li , W. Wang , X. Chen , J. Zheng , Z. Fan , Y. Jiang , Y. Liao , Bioact. Mater. 2025, 52, 422. 40585387 10.1016/j.bioactmat.2025.06.004 PMC12206045

[17] W. Zhang , K. Cai , Z. Sun , Q. Xiang , L. Yuan , M. Fu , X. Liu , M. F. F. Foda , Z. Ye , J. Huang , H. Liu , H. Han , H. Liang , H. Dong , X. Zhang , ACS Nano 2023, 17, 18932. 37768554 10.1021/acsnano.3c04175

[18] B. Vigani , S. Rossi , G. Sandri , M. C. Bonferoni , C. M. Caramella , F. Ferrari , Pharmaceutics 2020, 12, 859. 32927595 10.3390/pharmaceutics12090859 PMC7559482

[19] Y. Chen , W. Liu , S. Wan , H. Wang , Y. Chen , H. Zhao , C. Zhang , K. Liu , T. Zhou , L. Jiang , Q. Cheng , X. Deng , Adv. Funct. Mater. 2024, 34, 2309191.

[20] F. Alves , D. Lane , T. P. M. Nguyen , A. I. Bush , S. Ayton , Signal Transduction Targeted Ther. 2025, 10, 2. 10.1038/s41392-024-02088-5 PMC11696223 39746918

[21] B. R. Stockwell , J. P. F. Angeli , H. Bayir , A. I. Bush , M. Conrad , S. J. Dixon , S. Fulda , S. Gascon , S. K. Hatzios , V. E. Kagan , K. Noel , X. Jiang , A. Linkermann , M. E. Murphy , M. Overholtzer , A. Oyagi , G. C. Pagnussat , J. Park , Q. Ran , C. S. Rosenfeld , K. Salnikow , D. Tang , F. M. Torti , S. V. Torti , S. Toyokuni , K. A. Woerpel , D. D. Zhang , Cell 2017, 171, 273. 28985560 10.1016/j.cell.2017.09.021 PMC5685180

[22] J. Zheng , M. Conrad , Cell Metab. 2020, 32, 920. 33217331 10.1016/j.cmet.2020.10.011

[23] A. Goebel , M. Rauner , L. C. Hofbauer , T. D. Rachner , Biochim. Biophys. Acta Rev. Cancer 2020, 1873, 188351. 32007596 10.1016/j.bbcan.2020.188351

[24] J. Alfonso , Nat. Cancer 2024, 5, 1789. 39690217 10.1038/s43018-024-00849-3

[25] X. Ma , E. Bi , Y. Lu , P. Su , C. Huang , L. Liu , Q. Wang , M. Yang , M. F. Kalady , J. Qian , A. Zhang , A. A. Gupte , D. J. Hamilton , C. Zheng , Q. Yi , Cell Metab. 2019, 30, 143. 31031094 10.1016/j.cmet.2019.04.002 PMC7061417

[26] Y. Zeng , Y. Luo , K. Zhao , S. Liu , K. Wu , Y. Wu , K. Du , W. Pan , Y. Dai , Y. Liu , M. Ren , F. Tian , L. Zhou , C. Gu , Cancer Res. 2024, 84, 3402. 39047230 10.1158/0008-5472.CAN-23-3703

[27] Y. Gao , Y. A. Khan , W. Mo , K. I. White , M. Perkins , R. A. Pfuetzner , J. G. Trapani , A. T. Brunger , T. Nicolson , Cell Rep. 2023, 42, 112345. 37027300 10.1016/j.celrep.2023.112345 PMC10524599

[28] K. E. d. Visser , J. A. Joyce , Cancer Cell 2023, 41, 374. 36917948 10.1016/j.ccell.2023.02.016

[29] M. Shen , X. Jiang , Q. Peng , L. Oyang , Z. Ren , J. Wang , M. Peng , Y. Zhou , X. Deng , Q. Liao , J. Hematol. Oncol. 2025, 18, 40. 40188340 10.1186/s13045-025-01691-5 PMC11972543

[30] L. Wang , M. Y. Wen , X. T. Cao , Science 2019, 365, 656. 10.1126/science.aav0758 31320558

[31] C. G. Wang , Y. K. Guan , M. Z. Lv , R. Zhang , Z. Y. Guo , X. M. Wei , X. X. Du , J. Yang , T. Li , Y. Wan , X. D. Su , X. J. Huang , Z. F. Jiang , Immunity 2018, 48, 675. 29653696 10.1016/j.immuni.2018.03.017

[32] M. H. Abu Elella , O. M. Kolawole , Int. J. Biol. Macromol. 2024, 277, 134531. 39116977 10.1016/j.ijbiomac.2024.134531

[33] X. He , S. Zhang , Y. Tian , W. Cheng , H. Jing , Int. J. Nanomed. 2023, 18, 1433. 10.2147/IJN.S405020 PMC10042261 36992822

[34] J. Li , J. Wang , J. Zhang , T. Han , X. Hu , M. M. S. Lee , D. Wang , B. Z. Tang , Adv. Mater. 2021, 33, 2105999. 10.1002/adma.202105999 34651361

[35] L. Tang , L. Wang , X. Yang , Y. Fen , Y. Li , W. Feng , Prog. Mater. Sci. 2021, 115, 100702.

