# Supplementary material for: NIR‐Activatable Domino Cascade Catalysis Nanozyme Reactor for Multi‐Mechanism Synergistic Immunotherapy in Bladder Cancer
Source: Adv Sci (Weinh). 2025 Dec 12;13(18):e13913. doi: 10.1002/advs.202513913 (PMC13042462; doi:10.1002/advs.202513913)
Supplement: Supplementary file 1 — Supporting Information [file ADVS-13-e13913-s001.docx]

**NIR-Activatable Domino Cascade Catalysis Nanozyme Reactor for Multi-Mechanism Synergistic Immunotherapy in Bladder Cancer**

*Yongnan Jiang^†^, Qingling Zhang^†^, Yuhan Zhang^†^, Xinlu Yu, Bo Jia, Yulong Dong, Yalong Wu, Kelong Fan, Xinquan Gu*, Lei Ji*, Wei Jiang*, Bin Liu**

Dr. Y. Jiang., Y. Zhang., B. Jia., Y. Dong., Y. Wu., Prof. X. Gu., and B. Liu.

Department of Urology, China-Japan Union Hospital of Jilin University, Changchun, Jilin, China. Email: [guxq@jlu.edu.cn](mailto:guxq@jlu.edu.cn); [liubin6628@jlu.edu.cn](mailto:liubin6628@jlu.edu.cn).

Miss. X. Yu

Department of Otolaryngology, Second Hospital of Jilin University, Changchun, Jilin, China.

Dr. Q. Zhang

Department of Dermatology, China-Japan Union Hospital of Jilin University, Changchun, Jilin, China.

Dr. L. Ji

Department of Cardiology, China-Japan Union Hospital of Jilin University, Changchun, Jilin, China. Email: [jilei@jlu.edu.cn](mailto:jilei@jlu.edu.cn).

Prof. W. Jiang

State Key Laboratory of Metabolic Dysregulation & Prevention and Treatment of Esophageal Cancer, Tianjian Laboratory of Advanced Biomedical Sciences, Academy of Medical Sciences, Zhengzhou University, Zhengzhou 450052, China. Email: [weijiang@zzu.edu.cn](mailto:weijiang@zzu.edu.cn).

Prof. K. Fan

CAS Engineering Laboratory for Nanozyme, Key Laboratory of Biomacromolecules (CAS), CAS Center for Excellence in Biomacromolecules, Institute of Biophysics, Chinese Academy of Sciences, Beijing 100101, China.

**Experimental section**

**Materials and methods**

**Materials:** Colloidal silica (Ludox AM-40), cholesterol oxidase (ChOx), ammonium persulfate (APS), tetramethylethylenediamine (TEMED), 3,3′,5,5′-tetramethylbenzidine (TMB), 5,5′-dithiobis-(2-nitrobenzoic acid) (DTNB), and 5,5-dimethyl-1-pyrroline-N-oxide (DMPO) were purchased from Sigma-Aldrich (USA). Aniline monomer, FeCl_2_, MnCl_2_, ethanol, acetic acid, N-isopropylacrylamide (NIPAM), N-hydroxymethylacrylamide (NHMAM), N, N′-methylenebisacrylamide (BIS), and acetonitrile were obtained from Aladdin (Shanghai, China). Ammonia solution was purchased from Sinopharm Chemical Reagent Co., Ltd. (China). Chitosan, 4% paraformaldehyde, Giemsa stain, and crystal violet were acquired from Solarbio (Beijing, China). Matrix gel, cholesterol assay kits, CCK-8 assay kits, live/dead cell staining kits, Reactive oxygen species (ROS) detection kits, lipid peroxidation kits and apoptosis detection kit were purchased from Beyotime (Shanghai, China). Transwell cell culture inserts were obtained from NEST (Wuxi, China). Dulbecco’s modified Eagle’s medium (DMEM), phosphate-buffered saline (PBS), and fetal bovine serum (FBS) were obtained from Gibco (USA), while penicillin/streptomycin solution was purchased from Servicebio (China). FerroOrange and Filipin were supplied by MedChemExpress (USA). Primary antibodies against GPX4, FSP1, and GAPDH and secondary antibodies (anti-rabbit and anti-mouse IgG) were obtained from Servicebio (Wuhan, China); γ-H2AX, anti-CD59 was from Proteintech (Wuhan, China); antibodies against STING, TBK1, IRF3, and their phosphorylated forms were from Cell Signaling Technology (USA). Tribromoethanol was acquired from Avertin (Shanghai, China). All antibodies used in flow cytometry are listed in Table S3.

**Instruments:** A muffle furnace (KJ-M1700, Kejing, China) was used for high-temperature treatments. The hydrodynamic size and zeta potential of nanomaterials were measured using a Zetasizer (Nano ZS90, Malvern, UK). Transmission electron microscopy (TEM) imaging was performed on a Talos F200S (ThermoFisher, USA). X-ray photoelectron spectroscopy (XPS) was conducted using an ESCALAB 250Xi (ThermoFisher, USA), and Fourier transform infrared (FT-IR) spectra were collected using a Nicolet iS10 (ThermoFisher, USA). X-ray absorption fine structure (XAFS) spectra were acquired at the BL14W1 beamline at the Shanghai Synchrotron Radiation Facility (SSRF, China). Thermogravimetric analysis (TGA) was carried out on a TGA 5500 instrument (TA Instruments, USA). Dissolved oxygen levels were detected using a dissolved oxygen analyzer (HD-RY100, HORDE· ELECTRIC, China). Electron spin resonance (ESR) spectra were recorded using a Bruker A300 (Germany). Photothermal heating was induced with an 808 nm NIR laser (Diomede, China) and monitored using an infrared thermal imager (FLIR E6, FLUKE, USA). Morphology was further assessed using a field-emission scanning electron microscope (SU8010, Hitachi, Japan). UV–Vis absorption spectra were collected using a UV-2600 spectrophotometer (Shimadzu, Japan). The percentage of light transmittance was measured using a turbidimeter (TH-110, China). Rheological behavior was tested using a DHR-2 rheometer (TA Instruments, USA). Fluorescence imaging was performed using a fluorescence inverted microscope (Axio Observer 7, Zeiss, Germany), flow cytometry was conducted using a FACS Calibur system (BD Biosciences, San Jose, USA), and confocal laser scanning microscopy (CLSM) was performed with a STELLARIS8 DIV system (Leica, Germany). Fluorescence imaging was conducted using a Bio-Rad imaging system (USA). Quantitative real-time PCR (qPCR) was performed on a QuantStudio 5 Real-Time PCR System (Applied Biosystems, USA), and RNA concentration was measured using a NanoDrop spectrophotometer (ThermoFisher, USA). Tumor size in mice was assessed using a portable ultrasound system (DP-20Vet, Mindray, China)

**Synthesis of FMCC Nanozyme**

Colloidal silica (Ludox AM-40) was used as the silica source. Aniline monomers were polymerized on the surface of silica nanoparticles via chemical oxidative polymerization under acidic conditions, forming core–shell structured SiO_2_@PANI nanoparticles. The resulting SiO_2_@PANI was then treated with 1 M aqueous ammonia to induce deprotonation, yielding SiO_2_@EB-PANI. Subsequently, the deprotonated product was dispersed in acetonitrile, and FeCl_2_ was added at a molar ratio of Fe: PANI = 1:200. The mixture was stirred at room temperature for 12 h to allow Lewis’s acid doping of Fe^2+^ into the polymer, affording the SiO_2_@Fe-PANI precursor. This precursor was subjected to pyrolysis at 800 °C for 3 h under an inert argon atmosphere to obtain Fe-doped carbon/nitrogen materials (SiO_2_@Fe/CN). The SiO_2_ template was then partially etched by incubating the material in a 3 M NaOH/ H_2_O/ EtOH solution (v/v = 1:1) at 100 °C, producing Fe/CN materials containing residual silica.

To endow the material with enhanced catalytic and immunomodulatory functionalities, the Fe/CN was dispersed in an ethanol/water solution containing different concentrations of MnCl_2_ and stirred under mild conditions (room temperature or 50 °C) for 12 h. Mn^2+^ ions were incorporated via electrostatic interactions and coordination with nitrogen donor sites to form Mn–Nx moieties, yielding FeMn@CN. Finally, FeMn@CN was co-incubated with cholesterol oxidase (ChOx) under mildly acidic conditions at room temperature and slowly added dropwise into a 0.1%–0.5% chitosan solution prepared in 0.5% acetic acid. Through ionotropic gelation, a stable chitosan-based coating was formed on the nanoparticle surface, ultimately producing the multifunctional nanozyme system, termed FMCC.

**Labeling of FMCC Nanoparticles**

To fluorescently label FMCC nanoparticles, two different fluorescent dyes—fluorescein isothiocyanate (FITC) and Cy5.5—were conjugated separately to the chitosan shell. For FITC labeling, FMCC nanoparticles (5 mg) were dispersed in 5 mL of carbonate-bicarbonate buffer (0.1 M, pH 9.0) under gentle stirring. FITC (1 mg) was dissolved in 1 mL of anhydrous DMSO and added dropwise to the FMCC dispersion. The reaction mixture was stirred in the dark at room temperature for 12 h. For Cy5.5 labeling, a similar protocol was followed: Cy5.5-NHS ester (1 mg) was dissolved in DMSO and slowly added to the FMCC dispersion in carbonate buffer (pH 8.5), allowing the NHS ester to react with the primary amines on chitosan. After incubation, both mixtures were centrifuged (12,000 rpm, 10 min) and washed three times with deionized water and ethanol to remove unbound dye. The resulting fluorescently labeled nanoparticles (FITC-FMCC and Cy5.5-FMCC) were stored in PBS at 4 °C for further use.

**Synthesis of PNH Hydrogel and FMCC@PNH Composite Hydrogel**

PNIPAM-co-NHMAM (PNH) thermos-responsive hydrogels with various lower critical solution temperatures (LCST) were synthesized by copolymerizing N-isopropylacrylamide (NIPAM) and N-hydroxymethylacrylamide (NHMAM) in deionized water at different molar ratios. N, N’-methylenebisacrylamide (BIS) was added as a crosslinker, ammonium persulfate (APS) as an initiator, and the solution was degassed under vacuum. Subsequently, Tetramethylethylenediamine (TEMED) was introduced to initiate polymerization. The resulting hydrogels were thoroughly washed at low temperature to remove unreacted monomers. FMCC nanozyme was then incorporated into the hydrogel network via physical blending, forming a multifunctional composite hydrogel platform (FMCC@PNH) with integrated photothermal and enzymatic catalytic activities.

**Characterization of FMCC Nanozyme**

The hydrodynamic diameter and zeta potential of FMCC were measured using a Zetasizer (Nano ZS90, Malvern, UK). The morphology and structure were characterized by transmission electron microscopy (TEM, Talos F200S, ThermoFisher, USA), and elemental distributions were analyzed by energy-dispersive spectroscopy (EDS). X-ray photoelectron spectroscopy (XPS, ESCALAB 250Xi, ThermoFisher, USA) was used to analyze the valence states of elements, and Fourier-transform infrared spectroscopy (FT-IR, Nicolet iS10, ThermoFisher, USA) confirmed the surface functional groups. X-ray absorption fine structure (XAFS) spectroscopy (BL14W1, SSRF, China) was employed to investigate the metal coordination environments. Thermogravimetric analysis (TGA) was performed on a TGA 5500 instrument (TA Instruments, USA).

**Evaluation of FMCC Enzymatic Activity**

Peroxidase (POD)-like activity was assessed using the colorimetric oxidation of TMB. Glutathione oxidase (GSHox)-like activity was evaluated by quantifying residual GSH via DTNB-based colorimetry. Catalase (CAT)-like activity was measured using a dissolved oxygen meter by tracking O_2_ concentration. Reactive oxygen species (ROS) generation was analyzed by electron spin resonance using DMPO as a spin-trapping agent.

**Cholesterol Depletion Capability of FMCC**

FMCC (100 μg/mL), cholesterol oxidase (100 μg/mL), and cholesterol (10 μM) were incubated in PBS at 37 °C for 12 h. The residual cholesterol content was quantified using a commercial cholesterol assay kit (Beyotime, China).

**Photothermal Performance of FMCC Nanozyme**

An aqueous dispersion of FMCC (200 μg/mL) was irradiated using an 808 nm laser (1.0 W/cm^2^, Diomede, China), and infrared thermal images were recorded every 30 s using an infrared thermal imaging camera (FLIR E6, FLUKE, USA). The temperature–time curve was plotted accordingly. For photothermal stability testing, FMCC was subjected to five heating–cooling cycles under 808 nm laser irradiation (5 min irradiation at 1.0 W/cm^2^ followed by 10 min of natural cooling). Temperature data were collected every 30 s.

**Structural and Functional Characterization of FMCC@PNH**

Freeze-dried FMCC@PNH samples were characterized using a field-emission scanning electron microscope (SEM, SU8010, Hitachi, Japan) at an accelerating voltage of 5 kV. The LCST of the hydrogels was determined using a UV–vis spectrophotometer (UV-2600, Shimadzu, Japan). Hydrogels were loaded into quartz cuvettes, and the transmittance at 300 nm was monitored as the temperature was increased from 30 °C to 50 °C at a rate of 1 °C/min. The LCST was defined as the inflection point corresponding to a sharp decrease in transmittance, which was measured using a turbidimeter. Rheological behavior was tested at 25 °C using a rheometer (DHR-2, TA Instruments, USA) equipped with a 40 mm parallel plate and a 1 mm gap. Shear rate was varied from 0.01 s^-1^ to 100 s^-1^, and the viscosity was plotted on a logarithmic scale with a dual-axis display showing viscosity and shear stress.

**Photothermal Characterization of FMCC@PNH Composites**

Composite hydrogels containing different weight ratios of FMCC (0.1%, 0.2%, 0.4%, 0.8%, and 1.6%) were prepared and designated as FMCC@PNH-I through FMCC@PNH-V, respectively. Photothermal performance was evaluated using the same procedure as described for FMCC alone.

**FMCC Release from FMCC@PNH Hydrogel**

The release profile of FMCC from the FMCC@PNH composite hydrogel was determined using UV–vis spectrophotometry. The absorbance at 325 nm was recorded, and a calibration curve was established using FMCC solutions at concentrations ranging from 0 to 500 μg/mL. The concentration of FMCC released into phosphate-buffered saline (PBS) was calculated by comparing the absorbance values to the standard curve. For thermally triggered release experiments, FMCC@PNH was irradiated with an 808 nm NIR laser at an appropriate power density to elevate the hydrogel temperature. Subsequently, the concentration of FMCC released into PBS was quantified using the above method.

**Swelling Behavior of FMCC@PNH Hydrogel**

To evaluate the temperature-responsive swelling behavior of the PNH hydrogel, freeze-dried hydrogel samples were first weighed to determine their dry mass (Wd). The samples were then immersed in PBS (pH 7.4) at different preset temperatures. At predetermined time points (5, 10, 15, 30, 60, 120, 180, 240, and 300 min), the hydrogels were removed, gently blotted with filter paper to remove surface water, and weighed to obtain the swollen mass (Ws). The swelling ratio (SR) was calculated using the following equation:

$$\text{SR =(}\text{Ws}\text{ - Wd)/Wd}$$

All experiments were conducted in triplicate, and SR-time curves were plotted accordingly.

**Bladder Mucosal Permeability of FMCC Nanozyme**

Bladders were aseptically harvested from healthy C57BL/6 mice, and the bladder mucosa was carefully dissected and spread onto the upper chamber of a 24-well Transwell insert equipped with an 8 μm polycarbonate (PC) membrane (NEST, China), ensuring direct contact with the membrane surface.^[1]^ Serum-free medium was added to the upper chamber, while complete culture medium was added to the lower chamber. FMCC nanozymes (200 μg each), pre-synthesized in chitosan (CS) solutions of varying concentrations (0.05%, 0.1%, 0.3%, 0.5%, and 1%), were applied to the upper chamber. These formulations were designated as groups G1 to G5, respectively. After incubation at 37 °C for 3 h, the upper chambers were removed, and the FMCC concentration in the lower chamber was quantified to evaluate trans-mucosal permeability.

**Mucosal Penetration of FMCC@PNH Hydrogel**

Under sterile conditions, bladder tissues were isolated from healthy C57BL/6 mice. The bladder mucosa was dissected and placed onto the upper chamber of a 24-well Transwell insert with an 8 μm PC membrane, ensuring tight contact with the membrane surface. FMCC@PNH (100 μL) was added to the upper chamber, and 500 μL of complete medium was added to the lower chamber. The hydrogel was then irradiated with an 808 nm NIR laser (1.0 W/cm^2^) for 10 min, followed by incubation at 37 °C for 1 h. FMCC concentrations in the lower chamber were measured every 15 min to evaluate the dynamic penetration behavior.

**Ion Release Study by ICP-MS:**

FMCC nanoparticles (2 mg/mL) were dispersed in 5 mL of pH 7.4 PBS, pH 6.5 PBS, pH 6.5 PBS containing 10% FBS, or freshly collected mouse urine, respectively, and incubated at 37 °C with gentle shaking. At predetermined time points (1, 3, 7, and 14 days), samples were centrifuged (12,000 rpm, 10 min), and the supernatants were collected for analysis. The concentrations of Mn and Fe were quantified using inductively coupled plasma mass spectrometry (ICP-MS, Agilent 7900). Release percentages were calculated relative to the total elemental content determined after complete nanoparticle digestion in aqua regia.

**Cell Culture**

Mouse bladder cancer cells (MB49 **RRID:** **CVCL_7076**) and human embryonic kidney cells (HEK293 **RRID:** **CVCL_0045**) were obtained from the Cell Bank of the Chinese Academy of Sciences (Shanghai Institute of Biochemistry and Cell Biology, China). Mouse macrophages (RAW264.7 **RRID:** **CVCL_0493**) and immature bone marrow-derived dendritic cells (BMDCs) were kindly provided by Dr. Xu Zhou (Jilin University). All cells were cultured in high-glucose DMEM supplemented with 10% fetal bovine serum and 1% penicillin/streptomycin at 37°C in a humidified atmosphere containing 5% CO_2_.

**3D Tumor Spheroid Culture and Cy5.5-FMCC Treatment**

3D tumor spheroids were established using the ultra-low attachment (ULA) plate method. Briefly, MB49 bladder cancer cells were seeded into 96-well ULA round-bottom plates (Corning® Costar®, USA) at a density of 5 × 10^3^ cells per well in 200 μL of complete DMEM medium. The plates were centrifuged at 1000 rpm for 10 min to facilitate cell aggregation and then incubated at 37 °C with 5% CO_2_ for 4–5 days to allow spheroid formation. Then, Cy5.5-FMCC was diluted to 100 μg/mL in serum-free DMEM and gently added to each well (replacing half of the medium) to minimize disruption of the spheroid structure. For the light-activated treatment group, spheroids were incubated with Cy5.5-FMCC for 4 h, followed by near-infrared (NIR) laser irradiation at 808 nm (1.0 W/cm^2^) for 5 min. The control group received the same Cy5.5-FMCC treatment without NIR irradiation, under identical incubation conditions. After treatment, spheroids were further cultured for 4 h. After incubation, spheroids were carefully transferred to confocal culture dishes and then observed using a CLSM.

**Cellular Uptake of Cy5.5-FMCC**

MB49 cells were seeded in confocal culture dishes at a density of 1 × 10^5^ cells per well and incubated overnight. Cells were then incubated with Cy5.5-FMCC nanoparticles (50 μg/mL) in serum-free medium for 4 hours. After incubation, cells were washed three times with cold PBS to remove unbound nanoparticles and fixed with 4% paraformaldehyde for 15 min at room temperature. Subsequently, the cell membrane was stained with DiO (5 μM) for 15 min, followed by DAPI (1 μg/mL) staining for nuclear visualization. After final PBS washes, the samples were imaged using a CLSM to evaluate the cellular uptake and localization of Cy5.5-FMCC.

**Transmission Electron Microscopy (TEM) of Cells**

To observe the intracellular localization of FMCC nanoparticles and ultrastructural changes, transmission electron microscopy (TEM) was performed. MB49 cells were seeded in 6-well plates at a density of 2 × 10^5^ cells per well and incubated overnight. Cells were then treated with FMCC nanoparticles (50μg/mL) for 6 h. After treatment, cells were washed with PBS, harvested, and fixed with 2.5% glutaraldehyde in 0.1 M phosphate buffer (pH 7.4) at 4 °C overnight.

The fixed cells were washed three times with phosphate buffer and post-fixed with 1% osmium tetroxide (OsO_4_) for 1 h at room temperature. After additional washing, samples were dehydrated through a graded ethanol series (30%, 50%, 70%, 90%, and 100%), followed by infiltration with epoxy resin. Ultrathin sections (~70 nm) were cut using an ultramicrotome (Leica UC7), mounted on copper grids, and stained with uranyl acetate and lead citrate. Finally, the sections were observed using a transmission electron microscope (Hitachi HT7700, Japan) operating at 80 kV.

**Cytocompatibility and Cytotoxicity Assays**

For cytocompatibility testing, HEK293 cells were seeded at a density of 5 × 10^4^ cells per well into the lower chambers of a 24-well Transwell plate (8 μm PC membrane) and cultured overnight. Then, 200 μL of PBS (G1), PNH (G2), FC@PNH (G3), FMC@PNH (G4), or FMCC@PNH (G5) was added to the upper chambers. After 24 h of incubation, cell viability was assessed using the CCK-8 assay.

For cytotoxicity evaluation, MB49 cells were seeded at 5 × 10^4^ cells/well into the lower chambers of 24-well Transwell inserts (NEST, China) and incubated overnight. Subsequently, 200 μL of the following formulations were added to the upper chambers: PBS (G1), PNH + Laser (1.0 W/cm^2^, 5 min) (G2), FC@PNH + Laser (1.0 W/cm^2^, 5 min) (G3), FMC@PNH + Laser (1.0 W/cm^2^, 5 min) (G4), and FMCC@PNH + Laser (1.0 W/cm^2^, 5 min) (G5). After 24 h of co-incubation, the CCK-8 assay was performed to determine cell viability.

For live/dead staining, MB49 cells were treated as described above. After 24 h, the culture medium was removed, and the cells were washed three times with PBS. A Live/Dead Cell Staining Kit using Calcein-AM and Propidium Iodide (PI) was applied, and cells were incubated for 30 min at 37 °C. Fluorescence images were acquired using a fluorescence inverted microscope (FIM).

**Intracellular ROS Detection**

Intracellular ROS in MB49 cells were detected using the DCFH-DA fluorescent probe. After 24 h of treatment as described above, cells were incubated with DCFH-DA for 1 h at 37 °C. Fluorescence was observed via FIM, and ROS levels were quantitatively analyzed by flow cytometry.

**Intracellular Fe^2+^ Detection**

MB49 cells were seeded onto cell-climbing glass slides and incubated overnight. Treatments were applied to the upper chamber of Transwell inserts as follows: PBS (G1), PNH + Laser (1.0 W/cm^2^, 5 min) (G2), FC@PNH + Laser (1.0 W/cm^2^, 5 min) (G3), FMC@PNH + Laser (1.0 W/cm^2^, 5 min) (G4), and FMCC@PNH + Laser (1.0 W/cm^2^, 5 min) (G5), with 200 μL per group, followed by 24 h of co-incubation. Then, the upper chambers were removed, and 1 μM FerroOrange probe was added and incubated at 37 °C for 30 min to label intracellular Fe^2+^. Cells were fixed with 4% paraformaldehyde for 15 min and stained with DAPI for 10 min. The coverslips were carefully transferred onto microscope slides and observed using a CLSM.

**Cholesterol Staining**

MB49 cells were seeded onto cell-climbing glass slides and incubated overnight. The upper chambers were treated with 200 μL of ChOx (G1), PNH + Laser (1.0 W/cm^2^, 5 min) (G2), FC@PNH + Laser (1.0 W/cm^2^, 5 min) (G3), FMC@PNH + Laser (1.0 W/cm^2^, 5 min) (G4), or FMCC@PNH + Laser (1.0 W/cm^2^, 5 min) (G5) and co-incubated for 24 h. After removing the upper chambers, 50 μg/mL Filipin was added to stain cholesterol for 2 h. The coverslips were carefully transferred onto microscope slides and observed using a CLSM.

**Lipid Peroxidation Staining**

MB49 cells were seeded on climbing slides and cultured overnight. A positive control (2× LpoUp, G1) was added directly to the lower chamber. The upper chambers were treated with 200 μL of PNH + Laser (1.0 W/cm^2^, 5 min) (G2), FC@PNH + Laser (1.0 W/cm^2^, 5 min) (G3), FMC@PNH + Laser (1.0 W/cm^2^, 5 min) (G4), or FMCC@PNH + Laser (1.0 W/cm^2^, 5 min) (G5). After 12 h of incubation, cells were stained with 2 μM BODIPY 581/591 C11 at 37 °C for 30 min. The coverslips were carefully transferred onto microscope slides and observed using a CLSM.

**Fluorescence Quantification Analysis**

Fluorescence images were quantitatively analyzed using ImageJ software (NIH, USA). Images were first converted to 8-bit grayscale, and background noise was subtracted using the "Subtract Background" function. Regions of interest (ROIs) were selected manually or via threshold segmentation. Mean gray values and integrated densities were measured for each ROI. Corrected total cell fluorescence (CTCF) was calculated to eliminate background influence. All measurements were normalized for statistical comparison of fluorescence intensities across different groups.

**Cell Adhesion Assay**

The lower chambers of 24-well culture plates were precoated with 10 μg/mL fibronectin (FN) overnight at 4 °C. After aspiration, wells were blocked with 1% BSA at 37 °C for 1 h and rinsed three times with PBS. MB49 cells (5 × 10^4^ cells/well) were seeded in the pretreated lower chambers and cultured under the same experimental conditions as described above for 24 h. Cells were then fixed with 4% paraformaldehyde at room temperature for 30 min and stained with Giemsa solution for 15 min. Images were captured using an inverted microscope.

**Cell Invasion Assay**

After treatment according to the aforementioned grouping and protocols, MB49 cells were harvested by trypsinization. Matrigel was diluted with serum-free medium and added to the upper inserts of 24-well Transwell chambers (8 μm pore size), followed by incubation to allow gelation. Treated cells were resuspended in serum-free medium (200 μL) and seeded into the upper chambers, while 600 μL of complete medium was added to the lower chambers. After 24 h of incubation at 37 °C, non-invading cells on the upper surface of the membrane were removed with a cotton swab. Cells that had invaded through the membrane were fixed with 4% paraformaldehyde for 30 min and stained with 0.1% crystal violet for 20 min. Inserts were mounted on glass slides, and the invaded cells were observed using an inverted microscope.

**Cell Migration Assay**

After treatment following the protocol described above, MB49 cells were collected, washed, and resuspended in serum-free medium. A 24-well Transwell chamber was prepared by adding 700 μL of culture medium containing 20% FBS to the lower chamber as a chemoattractant. A total of 300 μL of the treated cell suspension was added to the upper chambers. After 12 h of incubation at 37 °C, non-migrated cells on the upper surface were removed. The migrated cells on the bottom side of the membrane were fixed with 4% paraformaldehyde for 40 min and stained with 0.1% crystal violet for 15 min. Membranes were dried, mounted on glass slides, and observed using an inverted microscope.

**Wound Healing Assay**

MB49 cells were seeded into 24-well plates and allowed to reach ~80% confluency overnight. A linear wound was created using the tip of a pipette. Cells were then rinsed with PBS and the upper chambers were treated with 200 μL of PBS (G1), PNH + Laser (1.0 W/cm^2^, 5 min) (G2), FC@PNH + Laser (1.0 W/cm^2^, 5 min) (G3), FMC@PNH + Laser (1.0 W/cm^2^, 5 min) (G4), and FMCC@PNH + Laser (1.0 W/cm^2^, 5 min) (G5) in the upper chambers of Transwell plates. After 24 h of incubation, the wound areas were imaged using an inverted microscope.

**Evaluation of Antitumor Immune Response in Vitro**

To evaluate the immunomodulatory effects, MB49 cells were seeded into the lower chambers of 12-well Transwell inserts (0.4 μm pore size, NEST, China) at a density of 2×10^5^ cells/mL in 1.5 mL of medium. According to the experimental groups, 500 μL of PBS (G1), PNH + Laser (1.0 W/cm^2^, 5 min) (G2), FC@PNH + Laser (1.0 W/cm^2^, 5 min) (G3), FMC@PNH + Laser (1.0 W/cm^2^, 5 min) (G4), and FMCC@PNH + Laser (1.0 W/cm^2^, 5 min) (G5) were added to the upper chambers and co-incubated for 24 h. After 24 h of incubation, the upper inserts were replaced with new ones containing 1×10^4^ RAW264.7 or BMDCs in 500 μL of medium. After an additional 24 h of co-culture, immune cells were collected for flow cytometry analysis.

**Western Blot Analysis**

For protein expression analysis, MB49 cells were seeded in the lower chambers of 12-well Transwell inserts (8 μm pore size) at a density of 2×10^5^ cells/mL with 1.5 mL of medium. The upper chambers were treated with 500 μL of the corresponding formulations according to the previously described experimental groups and protocols (G1–G5). After 24 h of treatment, the upper chambers were removed, and the cells in the lower chambers were lysed with RIPA buffer. Protein concentrations were determined using a BCA assay. Equal amounts of protein were mixed with loading buffer, separated via SDS-PAGE, and transferred onto PVDF membranes. The membranes were blocked with skim milk and incubated overnight at 4 °C with primary antibodies against GPX4 (1:1000), FPS1 (1:1000), CD59 (1:1000), γ-H2AX (1:5000), STING (1:1000), TBK1 (1:1000), and IRF3 (1:1000). For phosphorylated proteins, the membranes were stripped and re-incubated with anti-p-STING, anti-p-TBK1, and anti-p-IRF3 antibodies (all 1:1000) overnight at 4 °C. After washing with TBST, membranes were incubated with HRP-conjugated rabbit or mouse secondary antibodies (1:50000), and protein bands were visualized using a BIO-RAD imaging system.

**Quantitative Real-Time PCR (qPCR) Analysis**

MB49 cells were treated according to the previously described groups and protocols. Total RNA was extracted using TRIzol reagent, and RNA concentration and purity were assessed using a NanoDrop spectrophotometer. Equal amounts of RNA were reverse-transcribed into cDNA using a commercial reverse transcription kit. qPCR was performed using SYBR Green Master Mix on a real-time PCR system to quantify the mRNA levels of IL-6 and IFN-β, with GAPDH as the internal reference gene. Each sample was run in triplicate. The relative expression levels were calculated using the 2^−ΔΔCt method and reported as mean ± standard deviation (SD).

**In Vivo Evaluation of FMCC@PNH**

All animal experiments were conducted in strict accordance with the “Guidelines for the Care and Use of Laboratory Animals” and were approved by the Ethics Committee of Jilin University (Approval No. SY202407023). Male C57BL/6J mice (6–8 weeks old) were obtained from Henan Skobes Biotechnology Co., Ltd. (Anyang, China). To assess the biosafety of the FMCC@PNH system, mice were randomly divided into control and treatment groups. On days 0, 6, and 12, mice were subcutaneously injected with 1 mL of the respective formulations at a dose of 50 mg/kg body weight. On day 16, all mice were euthanized, and major organs (heart, liver, spleen, lungs, and kidneys) were collected for H&E staining. Additionally, blood samples were collected to analyze white blood cell and platelet counts, as well as liver and kidney function parameters, including aspartate aminotransferase (AST), alanine aminotransferase (ALT), blood urea nitrogen (BUN), and creatinine (CREA) levels.

**In Vivo Degradation of Hydrogel**

To assess the in vivo degradation of the hydrogel, 1mL of pre-formed hydrogel was subcutaneously injected into the dorsal region of C57BL/6J mice under tribromoethanol anesthesia. At predetermined time points (days 3, 7, 14, 21 and 28), the residual hydrogels were carefully excised and rinsed with PBS to remove surface impurities. The collected samples were lyophilized and weighed to determine the remaining dry mass. The degradation percentage was calculated by comparing the residual weight to the initial dry weight.

**Fluorescence Imaging of FMCC Penetration in Mouse Bladder Tissue**

To investigate the mucosal penetration of FMCC nanoparticles, FITC-FMCC (500 μg/mL, 100 μL) was intravesically instilled into the bladders of 6–8 weeks old BALB/c mice. After 1 h of retention, the bladders were harvested, gently washed with ice-cold PBS to remove unabsorbed nanoparticles, and embedded in optimal cutting temperature (OCT) compound. Samples were cryosectioned into 8 μm slices using a cryostat (Leica CM1950) and mounted onto glass slides. Tissue sections were fixed with 4% paraformaldehyde (PFA) for 15 min at room temperature, rinsed with PBS, and counterstained with DAPI (1 μg mL^-1^) for 5 min to visualize cell nuclei. Sections were imaged with a fluorescence microscope (Zeiss Axio Observer Z1) using appropriate filter sets.

**In Vivo Antitumor Efficacy in Bilateral Subcutaneous Bladder Cancer Model**

Specific pathogen-free (SPF) male C57BL/6J mice (6–8 weeks) were subcutaneously injected with 1×10^6^ MB49 cells in the right abdomen and 5×10^5^ MB49 cells in the left abdomen to establish a bilateral tumor model. On days 5, 10, and 15, mice were treated with 200 μL of PBS (G1), PNH + Laser (1.0 W/cm^2^, 5 min) (G2), FC@PNH + Laser (1.0 W/cm^2^, 5 min) (G3), FMC@PNH + Laser (1.0 W/cm^2^, 5 min) (G4), and FMCC@PNH + Laser (1.0 W/cm^2^, 5 min) (G5) via right-side intratumoral injection. The dose was equivalent to 10 mg/kg body weight (n = 18). Each group was randomly divided into A, B, and C subgroups (n = 6).

**Subgroup A:** On day 16, mice were euthanized and bilateral tumors were collected and minced. Tumor tissues were digested with collagenase IV (1 mg/mL) and DNase I (0.1 mg/mL) at 37 °C for 45 min, followed by gentle pipetting. The suspension was filtered through a 70 μm strainer and washed with PBS. Single-cell suspensions were stained with 7-AAD to exclude dead cells, followed by staining with Anti-CD3, anti-CD8, and anti-CD4 antibodies conjugated to APC, PE, and FITC, respectively, were used for staining. Samples were incubated at 4 °C in the dark for 30 min, then washed and analyzed using a flow cytometer (e.g., BD LSRFortessa). Live cells were gated by FSC/SSC and 7-AAD exclusion, followed by identification of CD3^+^, CD4^+^, and CD8^+^ T-cell populations. Single-stain and FMO controls were used for gating. FlowJo software was used for data analysis.

**Subgroup C:** Tumor volumes and body weights were recorded during treatment. Tumor volume was calculated as:V=width^2^×length×0.52. On day 25, mice were euthanized and tumors were weighed. Right-side tumors were sampled for qPCR analysis. To control for tumor size variation, 10 mg of tumor tissue from each sample was used for RNA extraction (TRIzol), and RNA quality was assessed by NanoDrop. cDNA was synthesized from 1 μg total RNA using a reverse transcription kit. SYBR Green-based qPCR was performed to assess target gene expression, with GAPDH as the internal reference. Each group was tested in triplicate, and relative expression was calculated using the 2^–ΔΔCt method.

**Group C:** Mice were monitored daily for health status and survival over 60 days. Survival curves were plotted using the Kaplan–Meier method.

**Antitumor Effect in a Recurrent Bladder Cancer Model**

A subcutaneous tumor model was established by injecting 1 × 10^6^ MB49 cells into the right flank of specific pathogen-free (SPF) male C57BL/6J mice. Seven days later, most of the tumor was surgically resected to simulate tumor recurrence. On days 2, 7, and 12 post-surgery, mice received subcutaneous injections (200 μL, equivalent to 10 mg/kg body weight) of PBS (G1), PNH + Laser (1.0 W/cm^2^, 5 min) (G2), FC@PNH + Laser (1.0 W/cm^2^, 5 min) (G3), FMC@PNH + Laser (1.0 W/cm^2^, 5 min) (G4), and FMCC@PNH + Laser (1.0 W/cm^2^, 5 min) (G5) (n = 18 per group). Mice in each group were randomly divided into three subgroups (A, B, and C; n = 6 per subgroup).

**Subgroup A**: On day 16, mice were euthanized, and tumor tissues and spleens were harvested. Tissues were mechanically dissociated and enzymatically digested in a solution containing 1 mg/mL collagenase IV and 0.1 mg/mL DNase I at 37 °C for 45 min, with gentle pipetting to facilitate dissociation. After filtration through a 70 μm cell strainer and PBS washing, single-cell suspensions were obtained. To exclude dead cells, APC-Cy7 dye was added and incubated in the dark for 10 min. Tumor and spleen cells were then stained with fluorophore-conjugated antibodies cocktails. The tumor panel included Cy5.5-CD45, BV421-CD11c, APC-CD86, and PE-CD80. The spleen panel included APC-CD3, BV711-CD62L, and BV421-CD44. Staining was performed at 4 °C for 30 min in the dark, followed by PBS washing.

Flow cytometry was performed using a BD LSRFortessa cytometer. Cell populations were gated based on FSC/SSC, and dead cells were excluded by gating out APC-Cy7^+^ cells. For lymph node samples, CD45^+^CD11c^+^ cells were gated, and the proportions of CD80^+^ and CD86^+^ cells were analyzed. For spleen samples, CD3^+^ T cells were selected, and the proportions of CD62L^+^ and CD44^+^ subsets were assessed. Single-stain and fluorescence-minus-one (FMO) controls were used to set compensation and gating thresholds. Data analysis was conducted using FlowJo software.

**Subgroup B**: Tumor volume and body weight were monitored during treatment. Tumor volume (mm^3^) was calculated as (width)^2^× (length) × 0.52. On day 25, mice were euthanized, and tumor weights were recorded. Tumor tissues were fixed in 4% paraformaldehyde for hematoxylin-eosin (H&E) staining and immunofluorescence. For immunofluorescence, sections underwent antigen retrieval, blocking, and staining with anti-CD8 fluorescent antibody, followed by DAPI nuclear counterstaining. Slides were sealed and imaged using a fluorescence microscope. H&E staining was performed following standard deparaffinization, hydration, staining, and mounting procedures. Tumor structure, cellular morphology, and necrotic regions were examined under a light microscope.

**Subgroup C**: Mice were monitored daily for health status and survival over 60 days. Survival curves were plot-ted using the Kaplan–Meier method.

**In Vivo Antitumor Efficacy in an Orthotopic Bladder Cancer Model**

Male SPF-grade C57BL/6J mice were used to establish an orthotopic bladder tumor model. Under anesthesia, a lower abdominal incision was made to expose the bladder. A fine needle was then used to inject 1 × 10^6^ MB49 bladder cancer cells into the bladder wall to initiate tumor development. On days 3, 6, 9, and 12 post-inoculation, intravesical instillation was performed using a 26G indwelling intravenous needle (Huaren, China). Prior to instillation, the bladder was rinsed three times via the urethra with PBS to remove residual urine. Mice were then treated with 200 μL of the following formulations: PBS (G1), PNH + Laser (1.0 W/cm^2^, 5 min) (G2), FC@PNH + Laser (1.0 W/cm^2^, 5 min) (G3), FMC@PNH + Laser (1.0 W/cm^2^, 5 min) (G4), and FMCC@PNH + Laser (1.0 W/cm^2^, 5 min) (G5). The dose was standardized to 10 mg/kg body weight (n = 18 per group). Each group was further divided randomly into three subgroups (A, B, C; n = 6 per subgroup).

**Subgroup A**: On day 16, bladder tumor tissues and inguinal lymph nodes were carefully dissected and then finely minced into small pieces, followed by enzymatic digestion with collagenase IV (1 mg/mL) and DNase I (0.1 mg/mL) at 37 °C for 45 min to yield single-cell suspensions. The dissociated cells were filtered through a 70 μm cell strainer, washed, and centrifuged. Dead cells were excluded by staining with APC-Cy7 viability dye (BioLegend, USA) for 10 min in the dark. Subsequently, lymph node cells and bladder tumor cells were separately incubated with a mixture of fluorophore-conjugated surface antibodies for staining. The antibody panel included: CD45–Cy5.5, CD11c–BV421, CD86–APC, CD80–PE, CD11b–FITC, and F4/80–PE. Staining was performed at 4 °C for 30 minutes in the dark, followed by washing with PBS.Flow cytometric analysis was performed using an instrument such as the BD LSRFortessa. During data acquisition, cell populations were first gated based on FSC/SSC parameters, and dead cells (APC-Cy7^+^) were excluded. Only live cells were included in subsequent analysis.CD45^+^CD11c^+^ dendritic cells were gated to assess the proportions of CD80^+^ and CD86^+^ subpopulations. Similarly, CD45^+^CD11b^+^ macrophages were gated for further analysis of F4/80^+^ and CD86^+^ subpopulations.Fluorescence compensation and gating were calibrated using single-stained and fluorescence-minus-one (FMO) controls. Data analysis was performed using FlowJo software (BD, USA).

**Subgroup B:** Mice were monitored for body weight throughout the treatment period to assess systemic toxicity. Tumor volume was non-invasively evaluated using an ultrasound imaging system at predetermined time points. Tumor dimensions, including length, width, and height, were obtained from ultrasound images, and tumor volume was calculated using the ellipsoid formula:

Volume = (length × width^2^) / 2

On day 21, urine samples were collected via gentle abdominal massage and analyzed immediately using commercial urine dipsticks (Multistix, Siemens, Germany) to assess hematuria. Red blood cell content was evaluated semi-quantitatively and recorded as negative (−), trace (±), or positive (+ to +++), indicating increasing severity. Mice were monitored daily for general health and survival over a 60-day period. Survival curves were plotted using the Kaplan–Meier method. Then mice were euthanized, and bladder tissues were harvested for hematoxylin and eosin (H&E) staining. Standard protocols were followed for paraffin embedding, deparaffinization, rehydration, staining, and mounting. Histopathological features, including tumor architecture, cellular morphology, and necrotic regions, were evaluated under a light microscope. In addition, terminal deoxynucleotidyl transferase dUTP nick end labeling (TUNEL) staining was performed on adjacent sections to evaluate apoptosis within the tumor tissues. TUNEL assays were conducted using a commercially available apoptosis detection kit according to the manufacturer's instructions. Following staining, cell nuclei were counterstained with DAPI apoptotic nuclei, (TUNEL-positive) were visualized via fluorescence microscopy.

**Subgroup C:** Mice were monitored daily for health status and survival over 60 days. Survival curves were plot-ted using the Kaplan–Meier method.

**Statistical Analysis**

Data are presented as the means ± SDs. ANOVA or Student's t-tests were used for comparisons between groups. Statistical significance was considered when *p < 0.05, **p < 0.01, ***p < 0.001, and ****p < 0.0001. “ns” indicates no statistical significance. All the data were analyzed with GraphPad Prism 8.**Supplementary Figures and Tables**

**
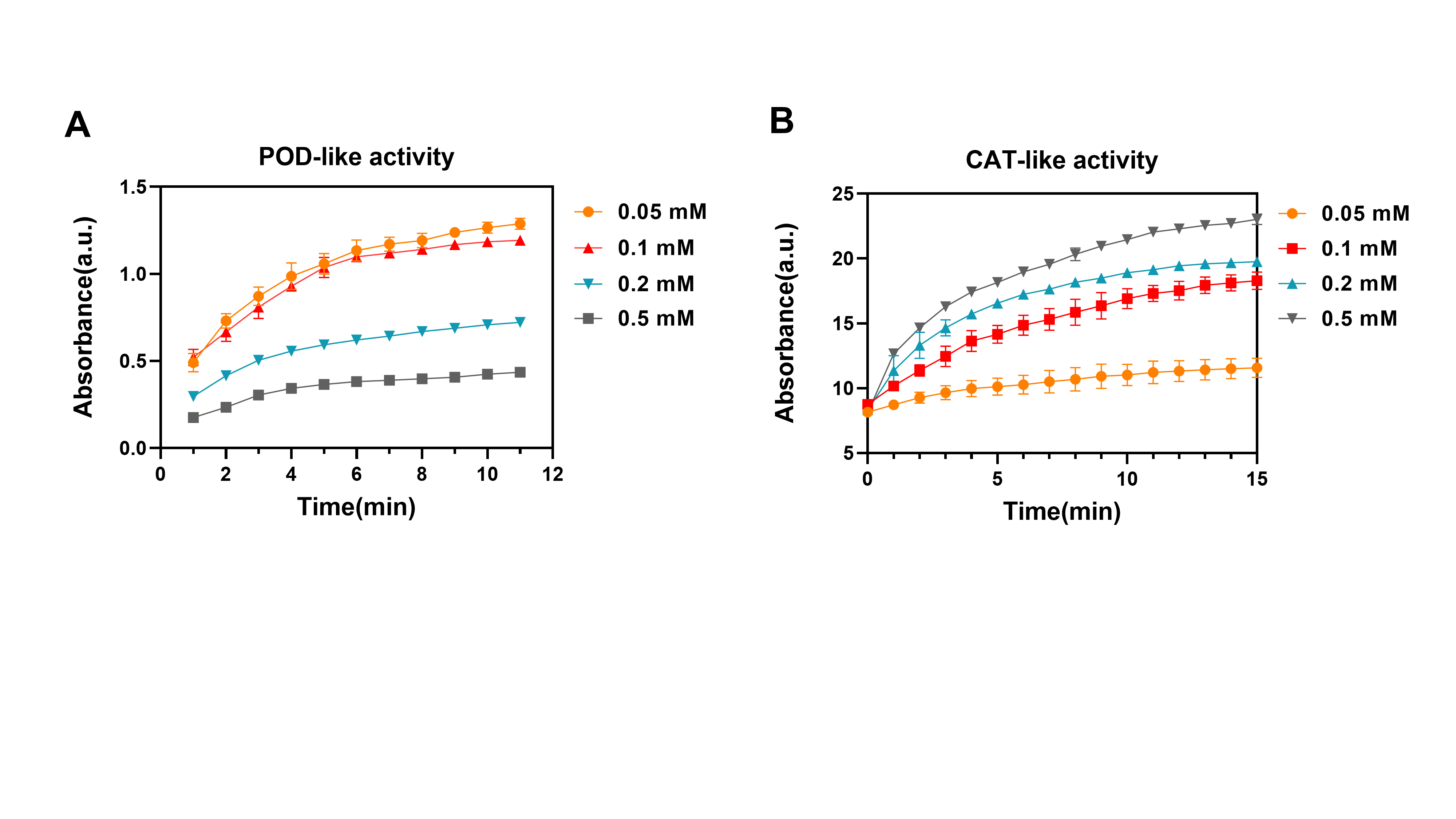
**

**Figure S1.** POD- (A) and CAT-like (B) activities of FMCC nanozymes synthesized with varying concentrations of MnCl_2_ solutions (n = 3).


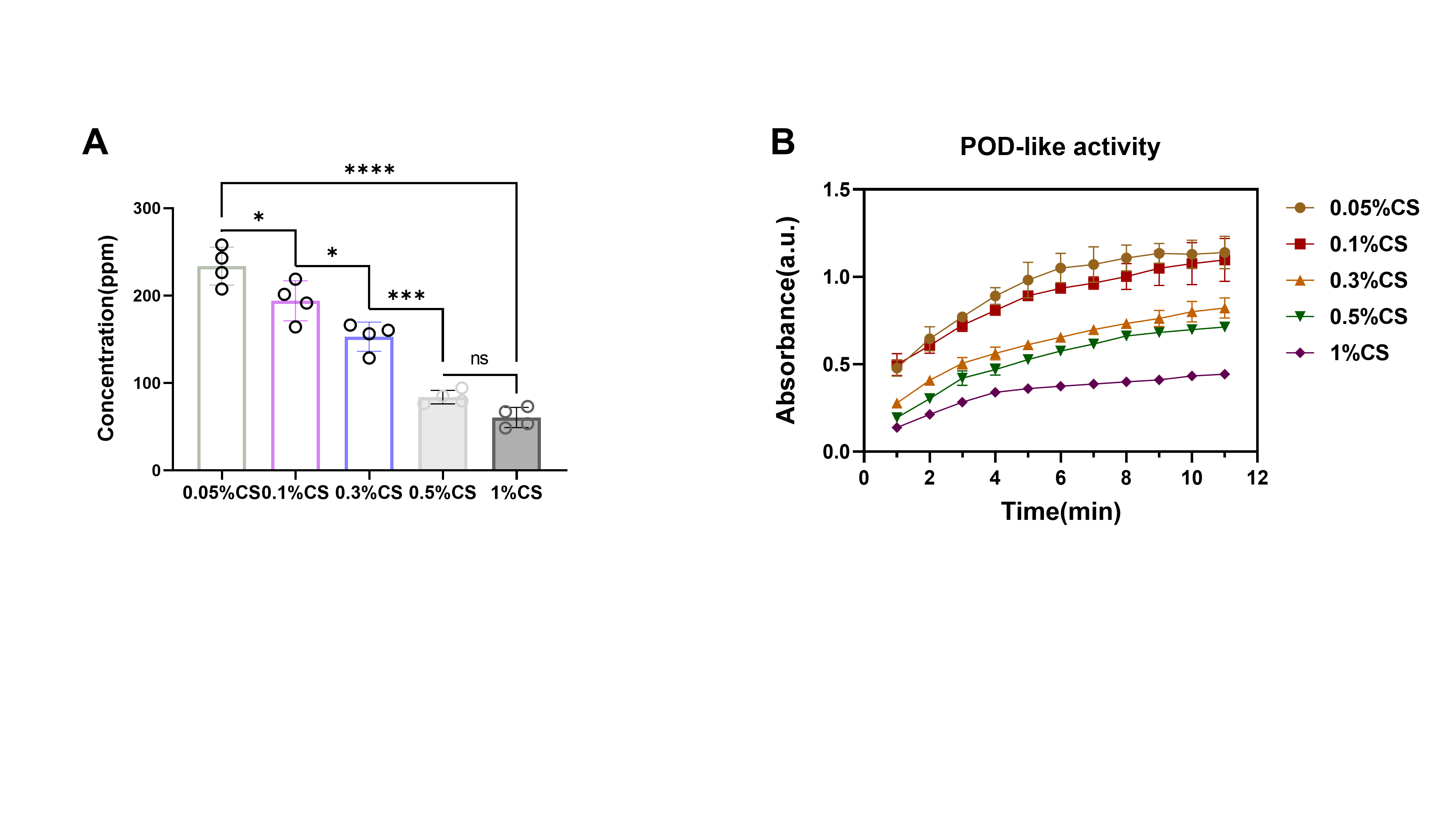


**Figure S2.** Effect of FMCC synthesized with different concentrations of chitosan on bladder mucosal penetration and POD-like activity. (A) The transwell-based evaluation of mucosal penetration in vitro by FMCC nanozymes synthesized in chitosan solutions of varying concentrations, with quantification based on their concentrations in the lower chamber. B, Evaluation of POD-like activity of FMCC nanozymes synthesized in chitosan solutions with different concentrations. The data are presented as the means ± SDs and were analyzed by one-way two-sided analysis of variance (ANOVA) with GraphPad Prism software. *p < 0.05, **p < 0.01, ***p < 0.001, ****p < 0.0001, “ns” indicates no statistical significance.





**Figure S3.** Normalized Fe K-edge X-ray absorption near-edge structure (XANES) spectra of FMCC compared with Fe foil, Fe_2_O_3_, and Fe-Pc, indicating the valence state and local coordination environment of Fe species in FMCC.


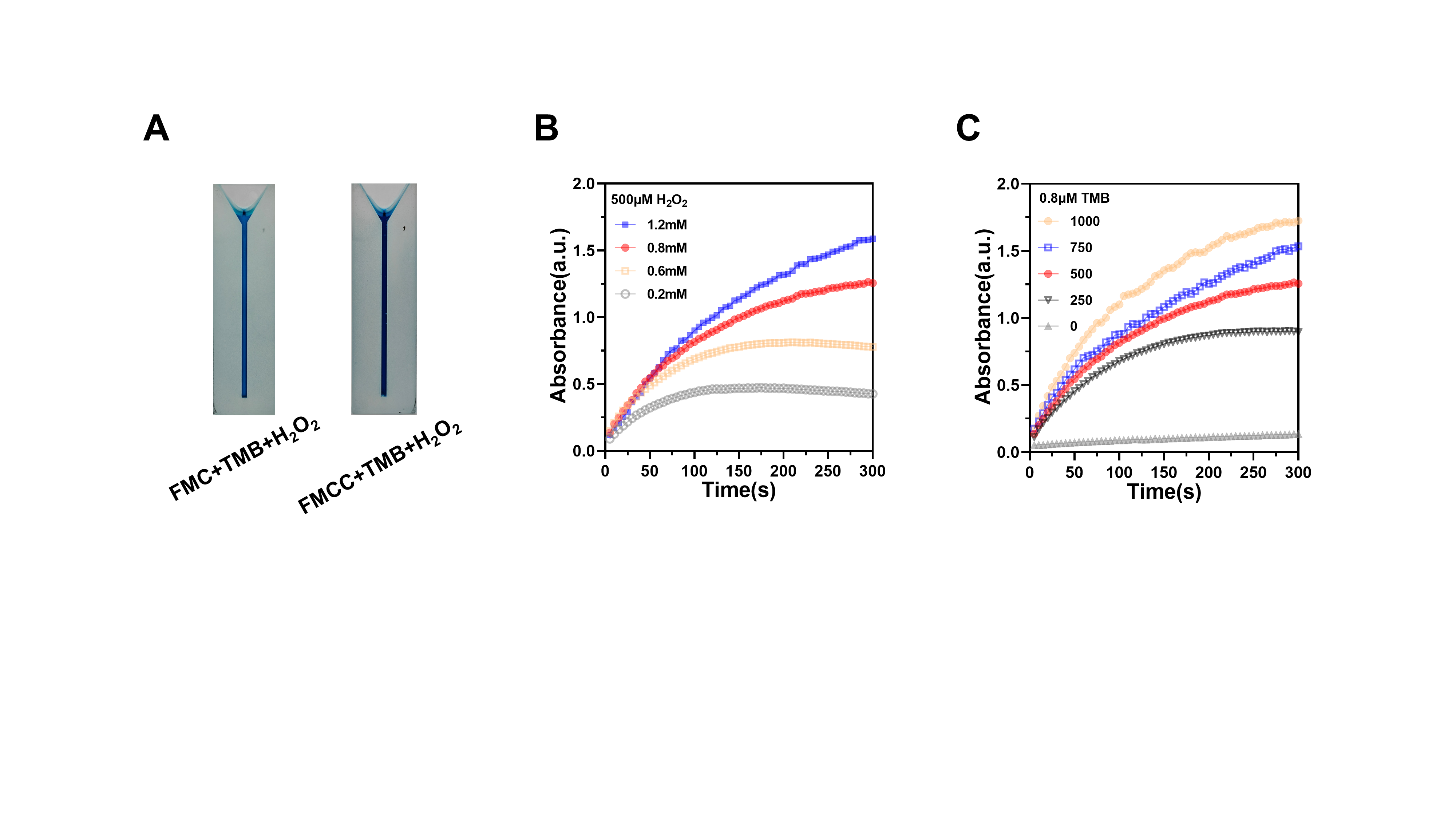


**Figure S4.** The development of blue coloration in cuvettes catalyzed by FMCC and FMC using TMB and H_2_O_2_ as substrates.


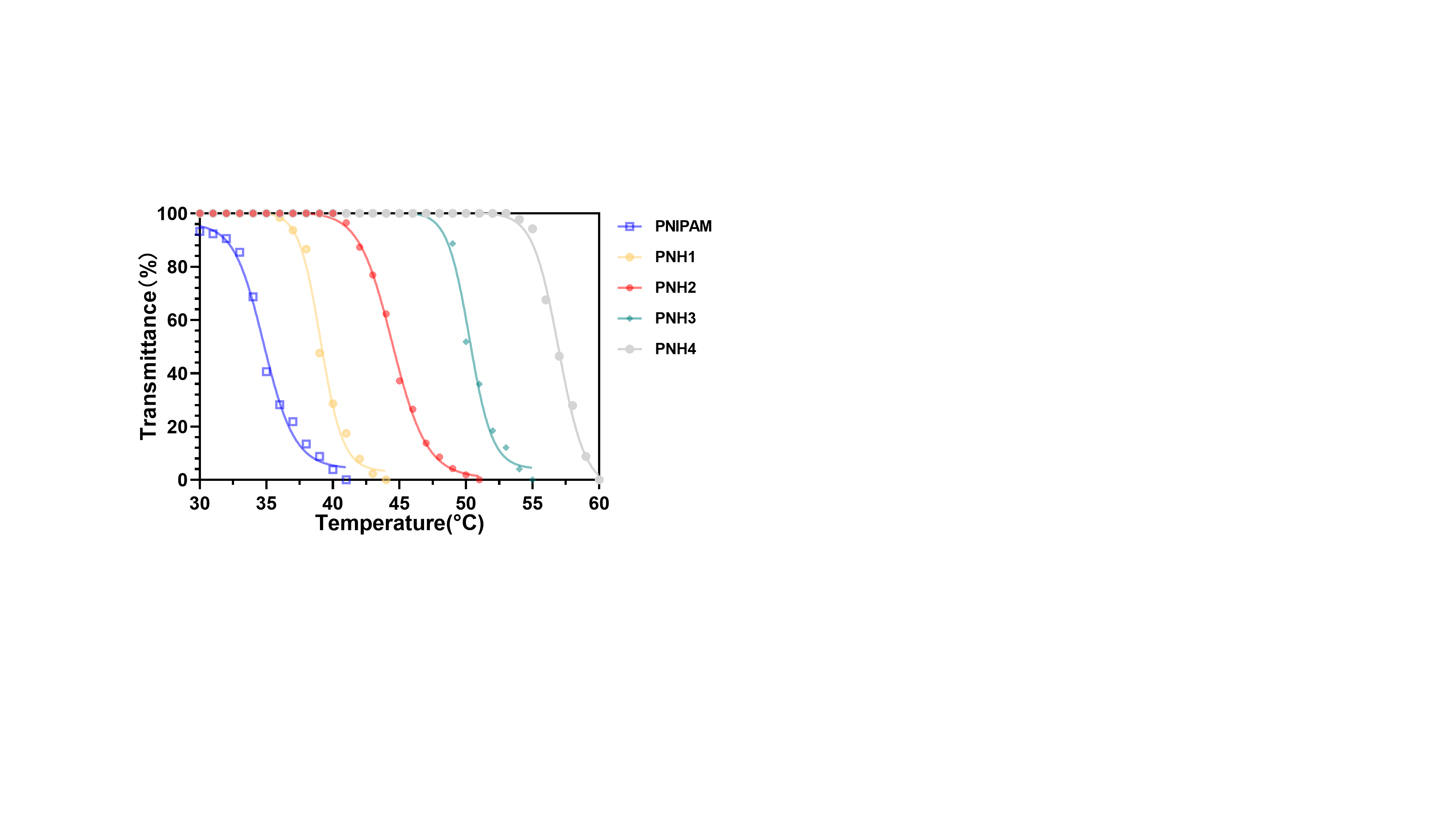


**Figure S5.** Thermo-responsive PNH hydrogels with various lower critical solution temperatures (LCSTs) were synthesized by copolymerizing N-isopropylacrylamide (NIPAM) and N-hydroxymethyl acrylamide (NHMA) at different molar ratios, yielding PNIPAM, PN1, PN2, PN3, and PN4, respectively. LCST is defined as the inflection point at which a sharp decline in transmittance occurs, as measured by a turbidimeter.


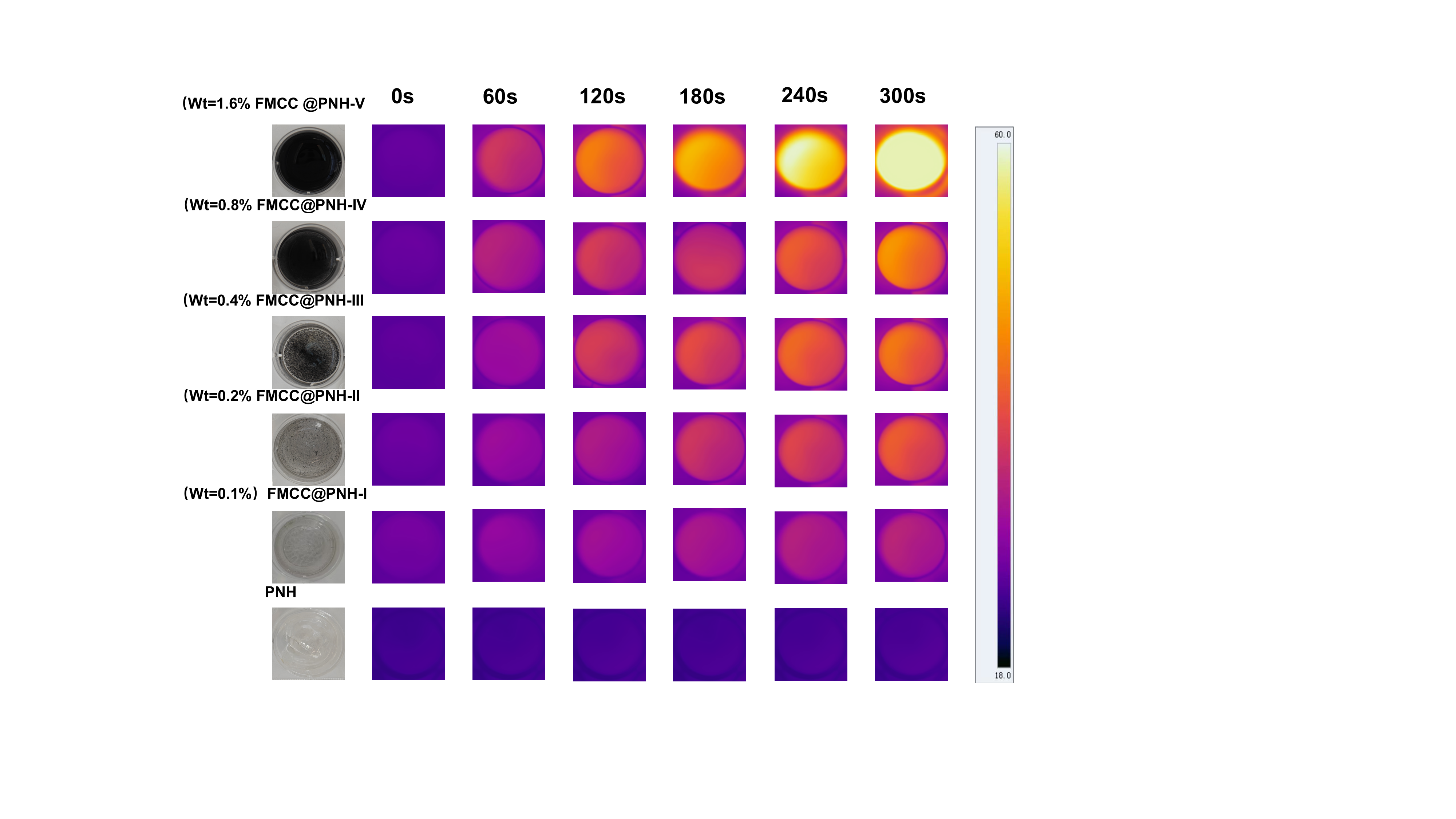


**Figure S6.** FMCC nanoparticles were incorporated into the PNH hydrogel at varying mass percentages, and thermal imaging was performed following 808 nm near-infrared (NIR) laser irradiation (1.0 W/cm^2^) .


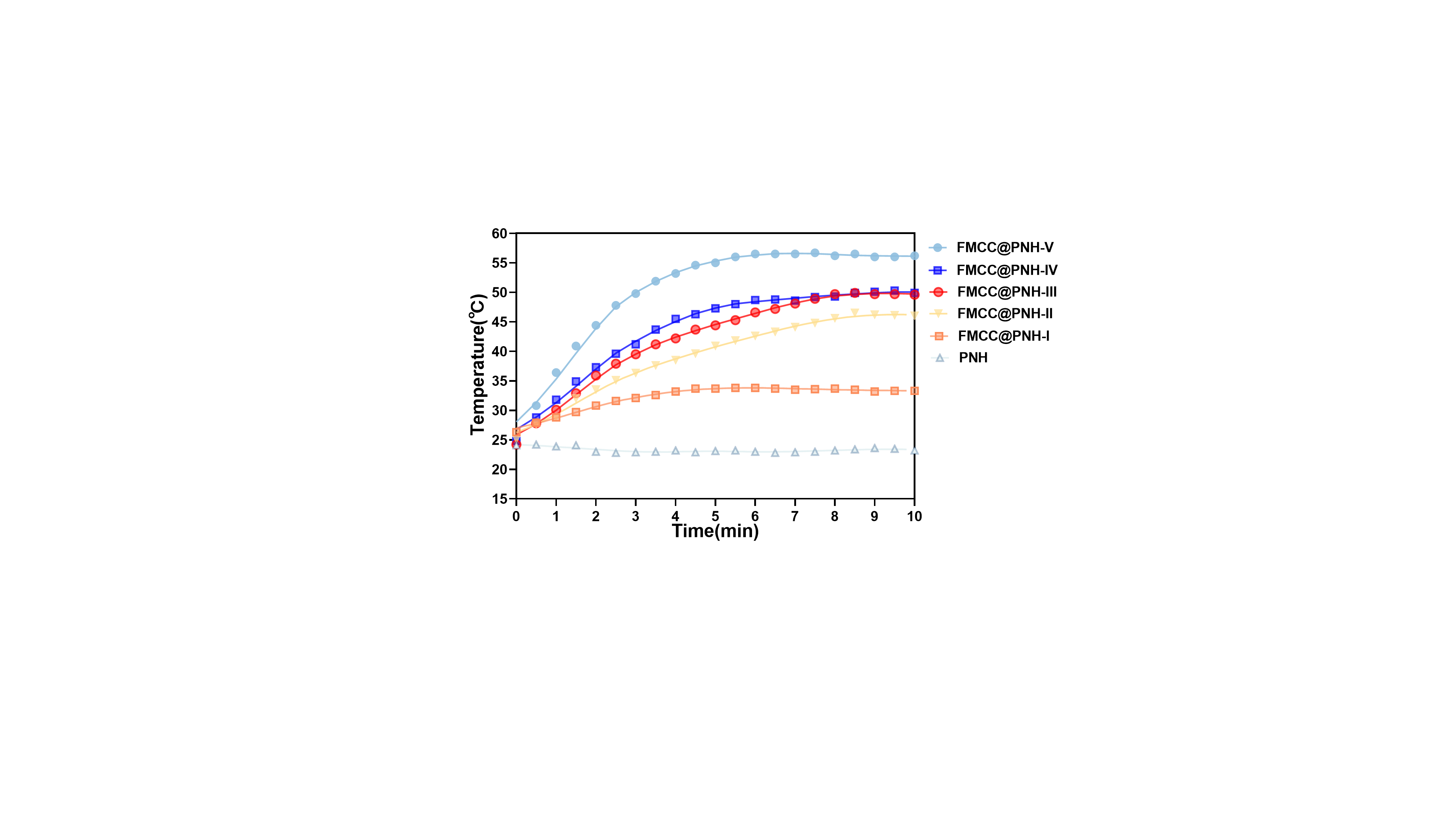


**Figure S7.** The temperature-time variation curves of each group.


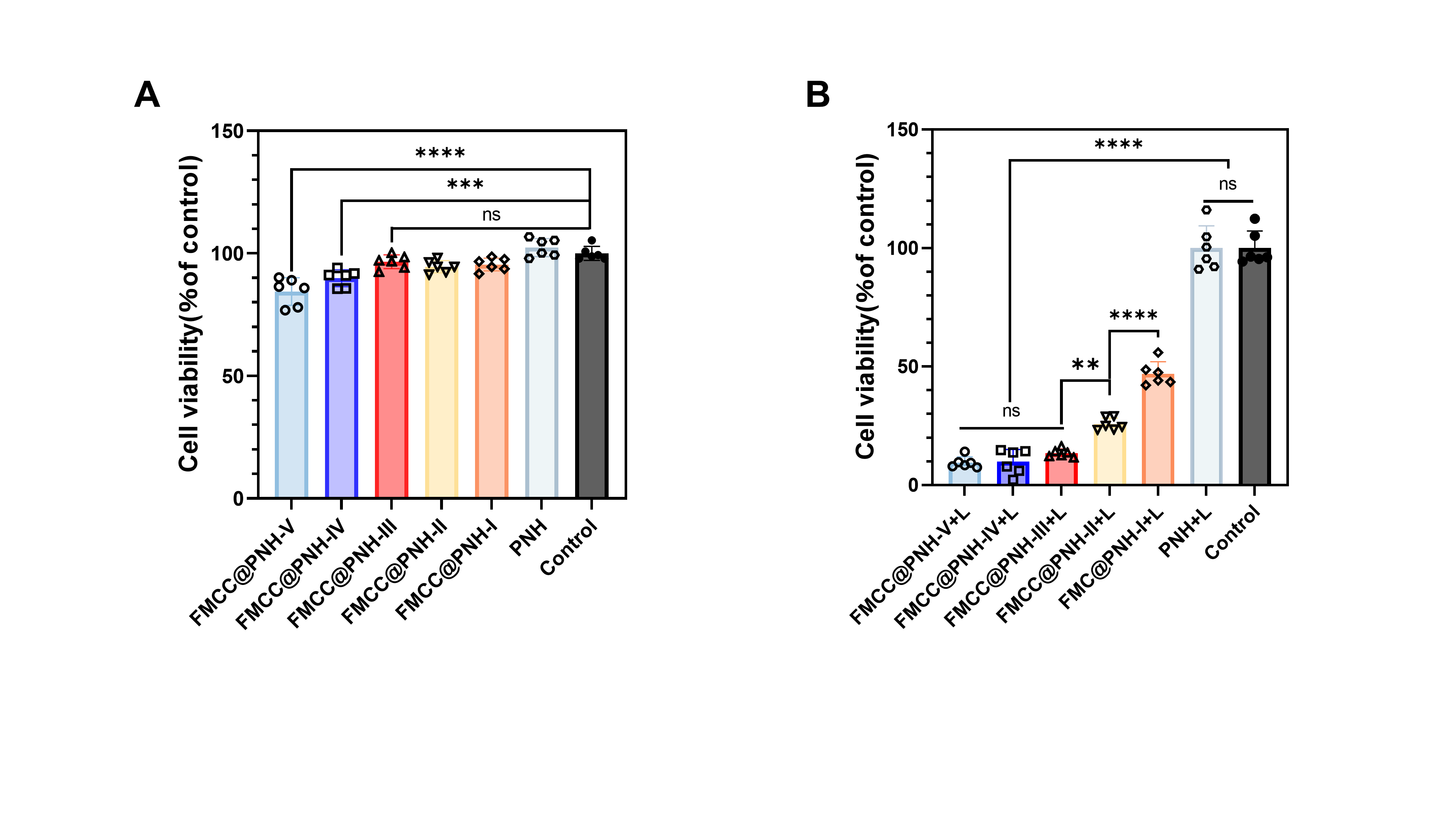


**Figure S8.** Cytocompatibility and Cytotoxicity Assays. (A) The cell viability of each group without laser irradiation was measured using the CCK-8 assay kit. (B) The cell viability of each group under 808 nm near-infrared light irradiation (1.0 W/cm^2^, 5min) was measured using the CCK-8 assay kit. The data are presented as the means ± SDs and were analyzed by one-way two-sided analysis of variance (ANOVA) with GraphPad Prism software. *p < 0.05, **p < 0.01, ***p < 0.001, ****p < 0.0001, “ns” indicates no statistical significance.


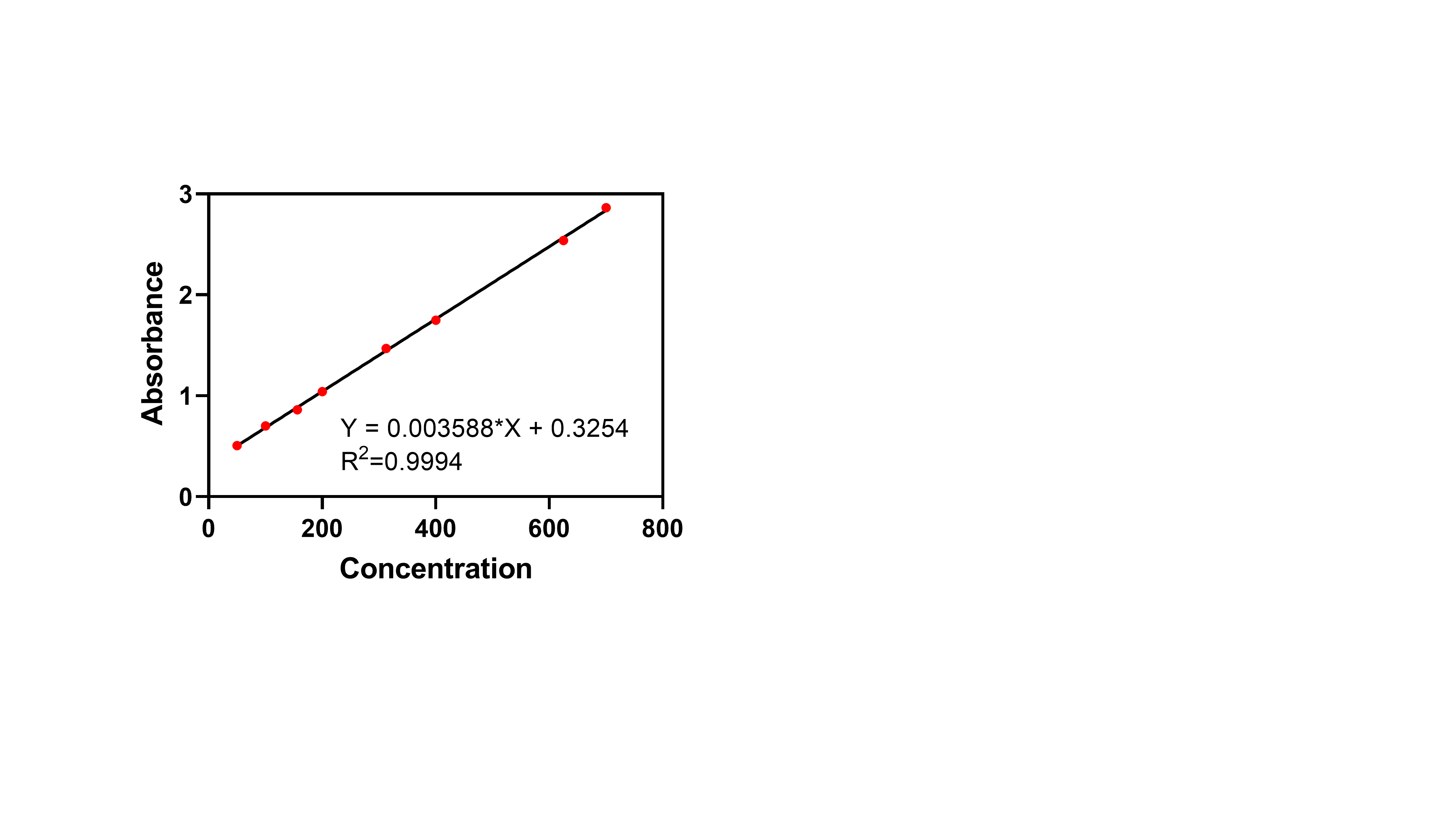


**Figure S9.** Standard calibration curve of FMCC.


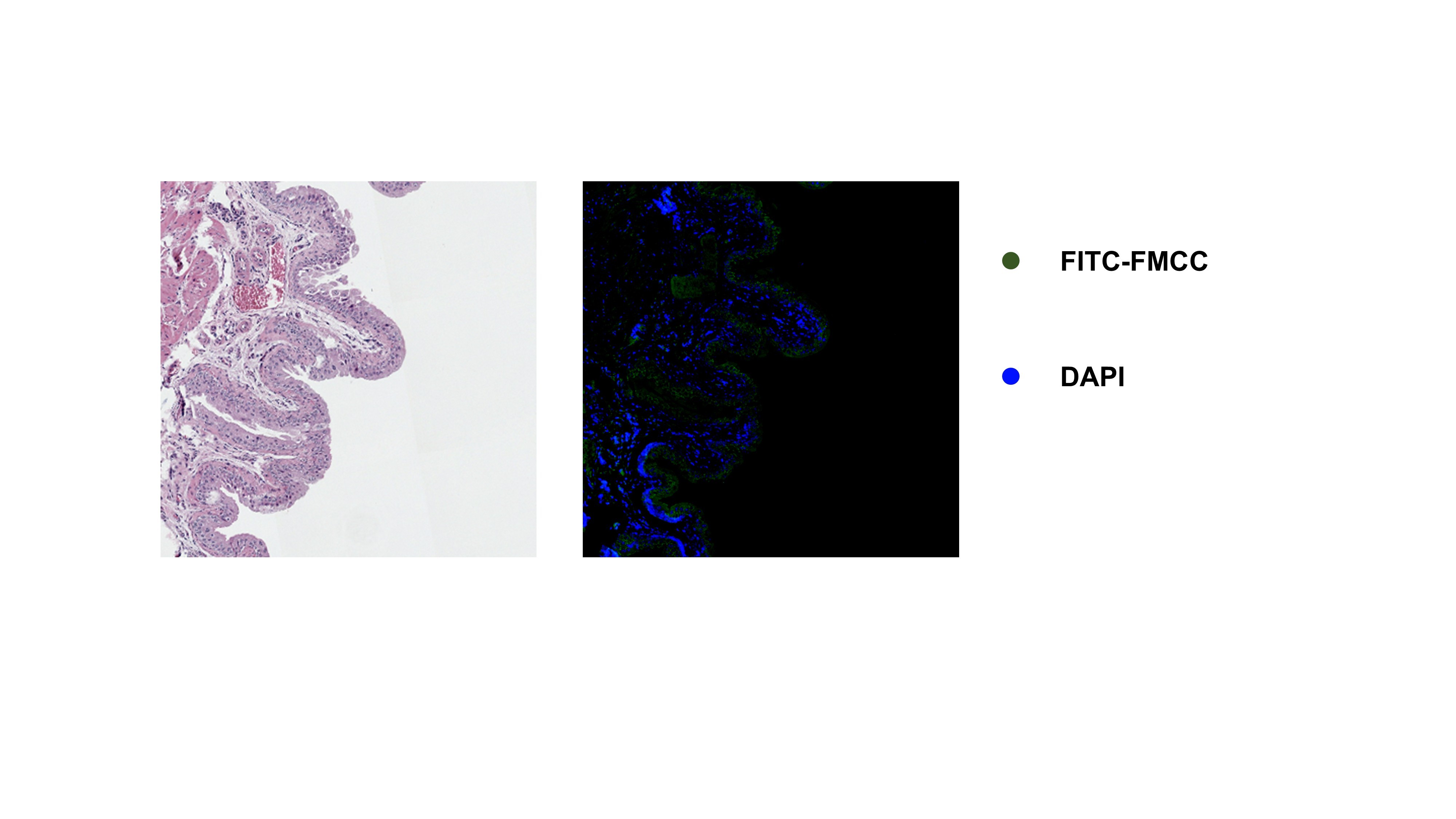


**Figure S10.** Fluorescence images demonstrating the penetration of FITC-labeled FMCC nanozymes in bladder mucosa.


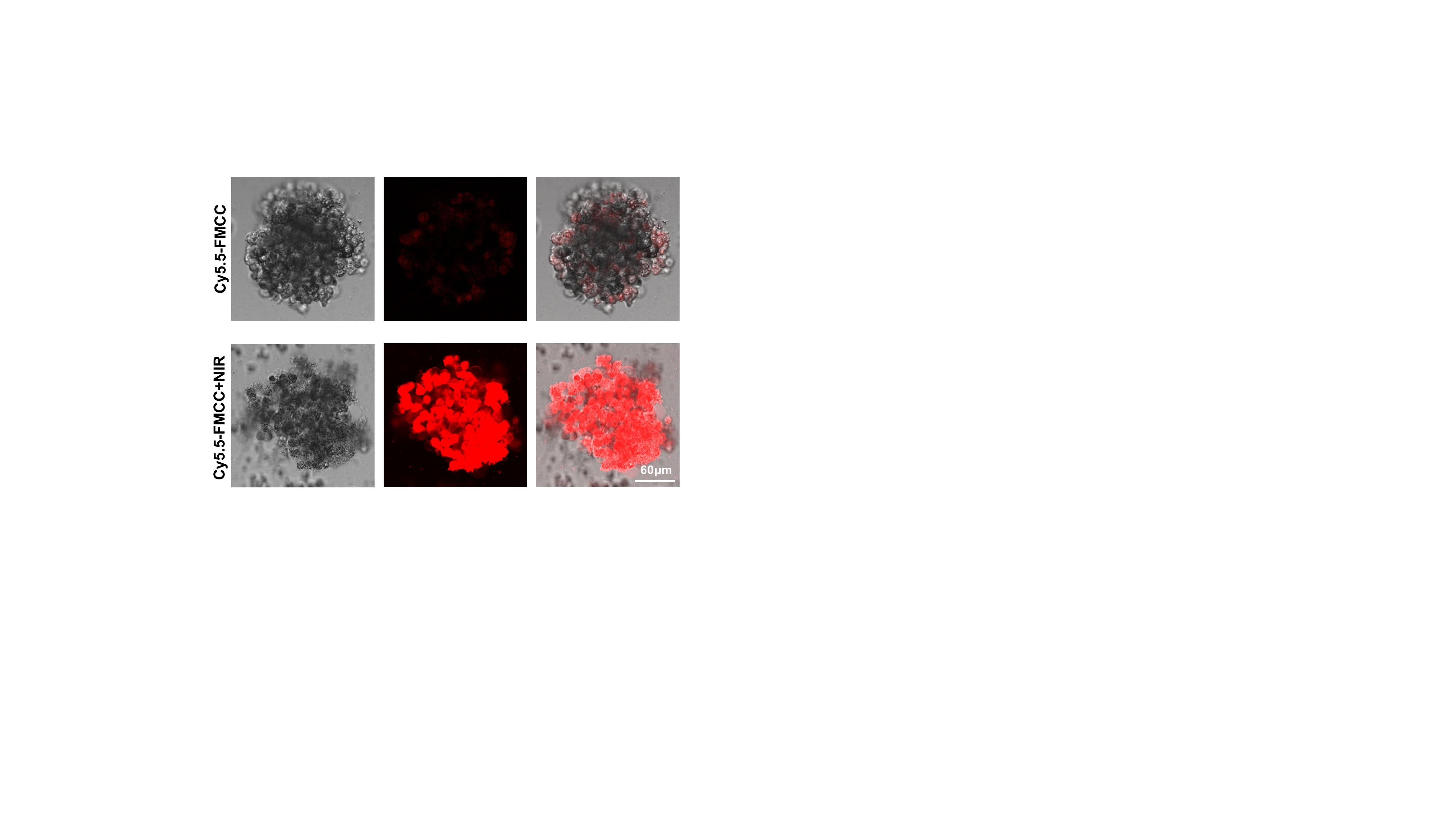


**Figure S11.** 3D tumor spheroid penetration assay was conducted to evaluate the ability of Cy5.5-FMCC nanoparticles to infiltrate the tumor mass.


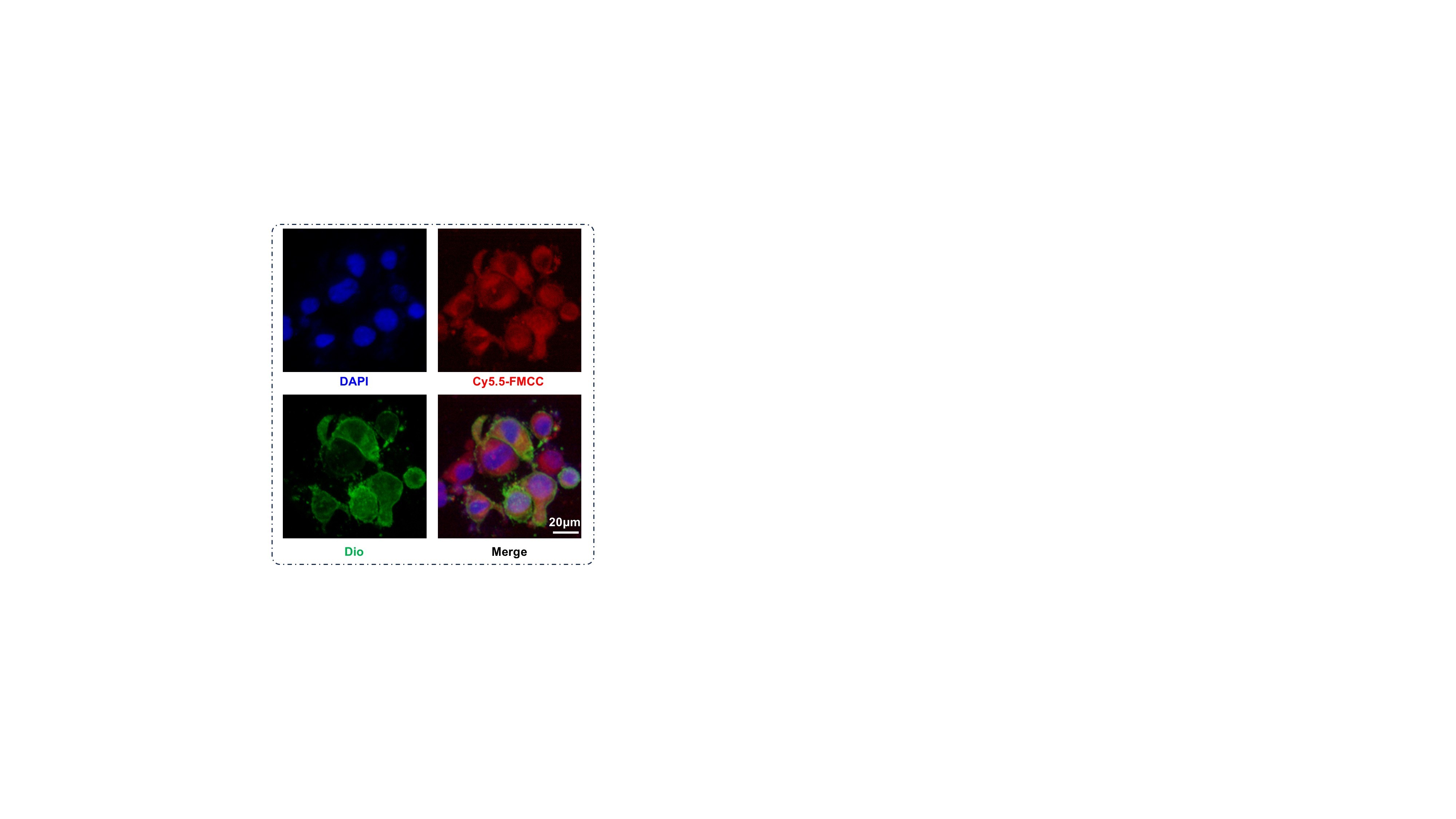


**Figure S12.** Cellular uptake assay confirmed the internalization of Cy5.5-FMCC into cells.


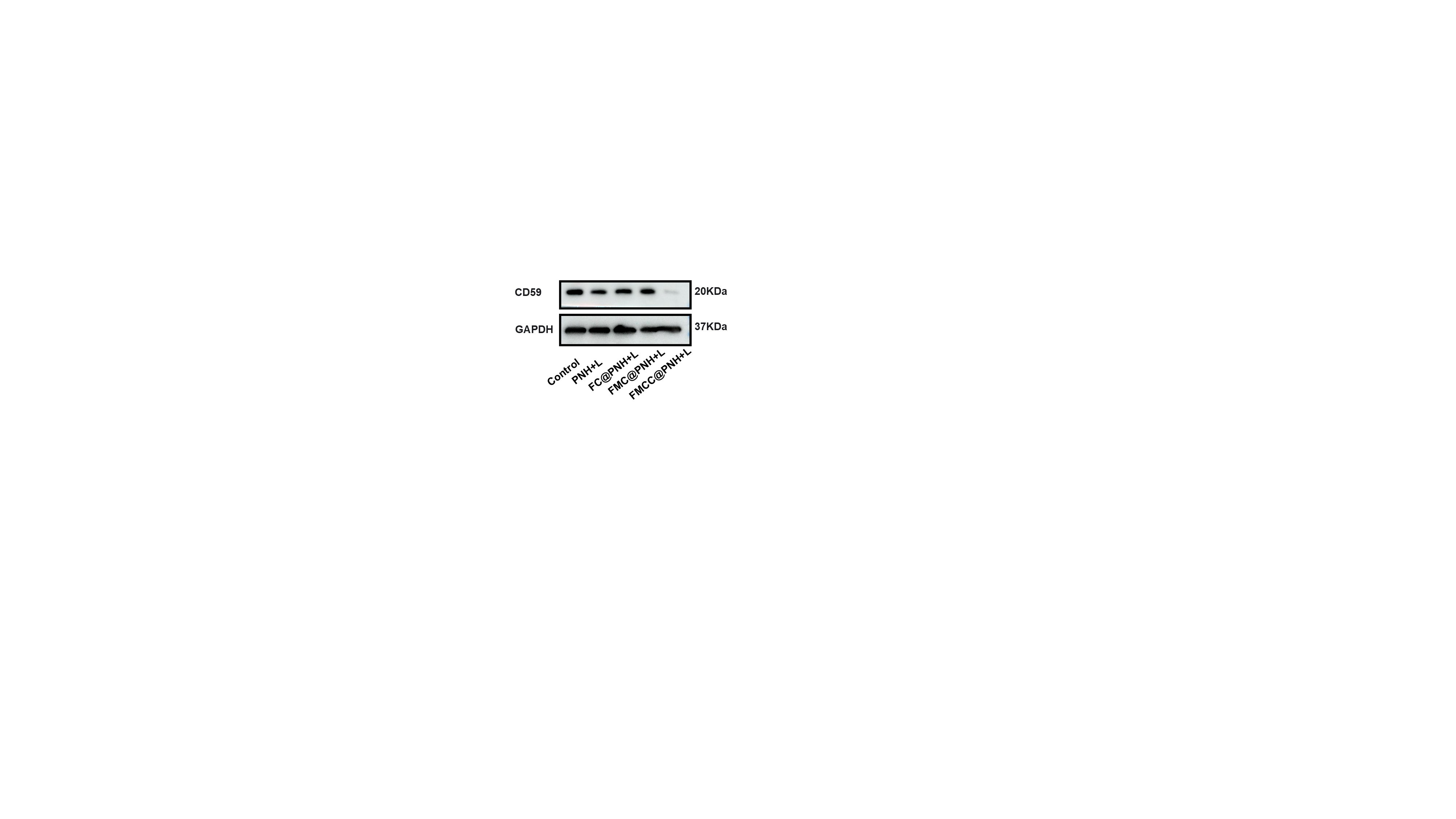


**Figure S13.** Western blotting analysis of the expression of CD59 in MB49 cells after different treatments.


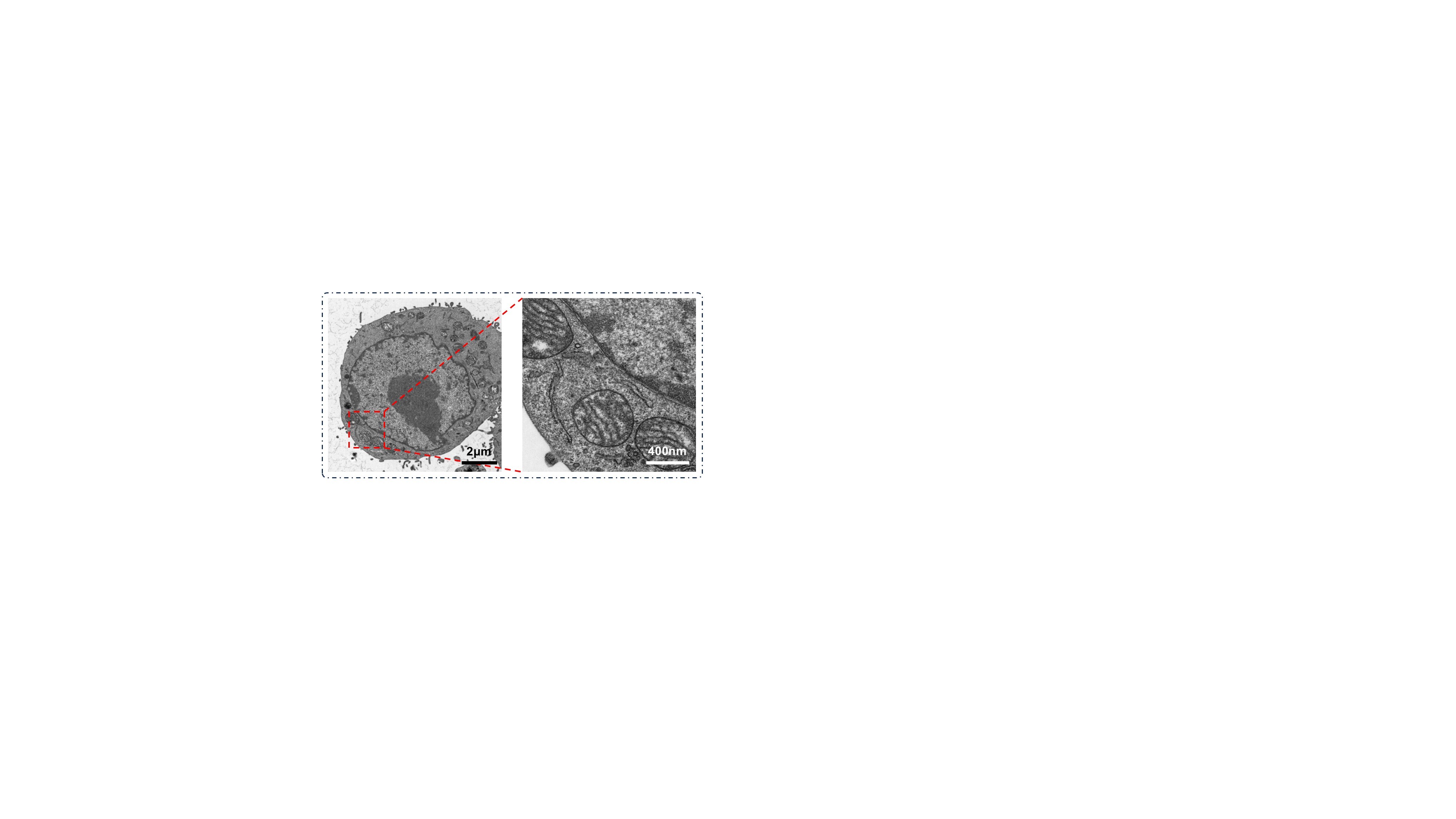


**Figure S14.** Representative TEM images showing the ultrastructural features and mitochondrial morphology of control group cells.


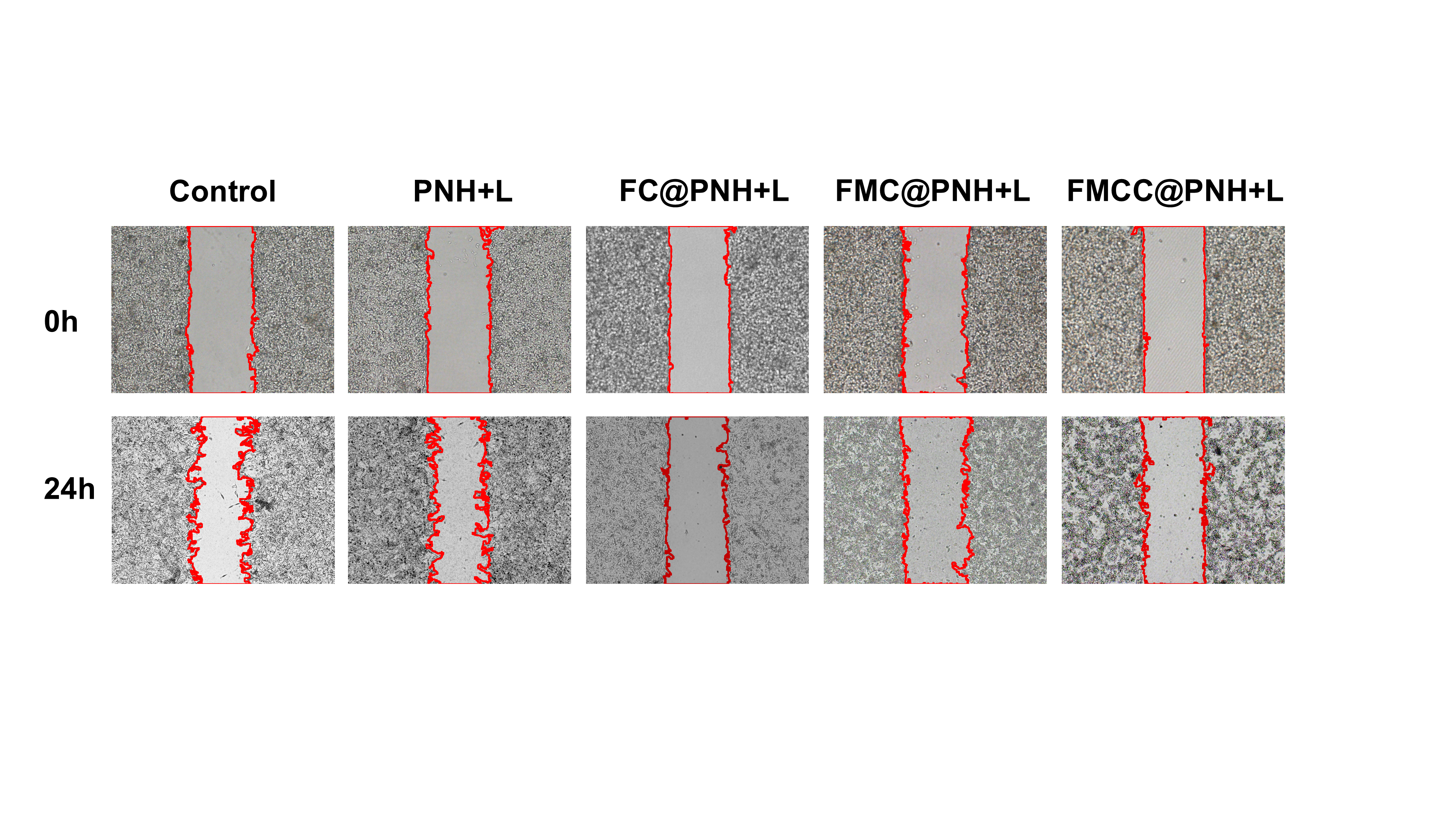


**Figure S15.** The cell scratch assay was used to evaluate the migration ability of cells after drug treatment in each group.


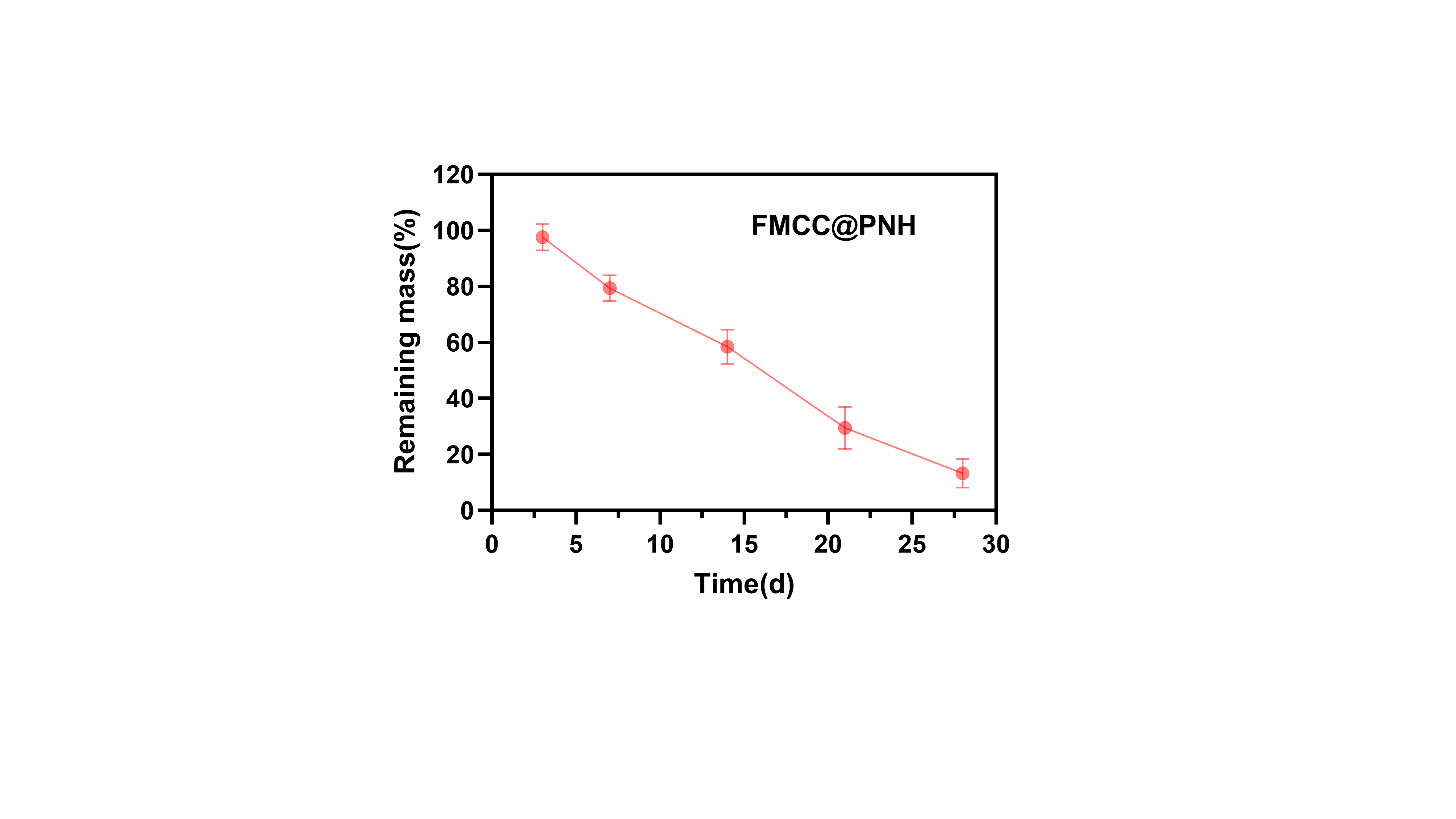


**Figure S16.** Degradation rate profile over time of FMCC@PNH nanozyme hybrid hydrogels subcutaneously implanted in mice.


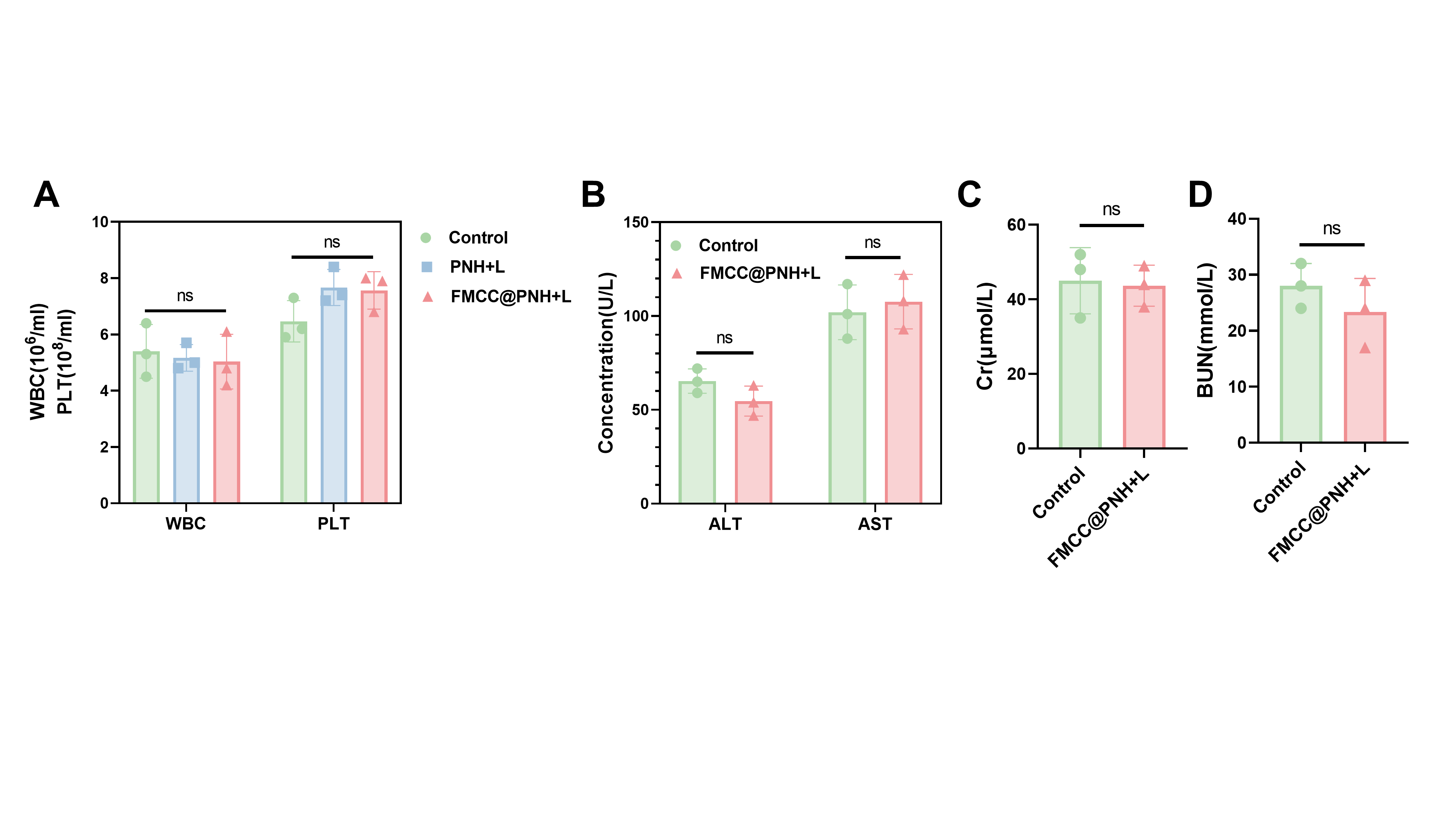


**Figure S17.** Safety evaluation. (A) Hematological analysis showing white blood cell (WBC) and platelet (PLT) counts across Control, PNH+L, and FMCC@PNH+L groups. (B) Serum biochemical analysis of liver function markers: alanine aminotransferase (ALT) and aspartate aminotransferase (AST). (C, D) Renal function indicators including serum creatinine (Cr) and blood urea nitrogen (BUN) levels in Control and FMCC@PNH+L groups. The data are presented as the means ± SDs and were analyzed using one-way two-sided analysis of variance (ANOVA) and unpaired two-tailed Student’s t-test with GraphPad Prism software. *p < 0.05, **p < 0.01, ***p < 0.001, ****p < 0.0001, “ns” indicates no statistical significance.


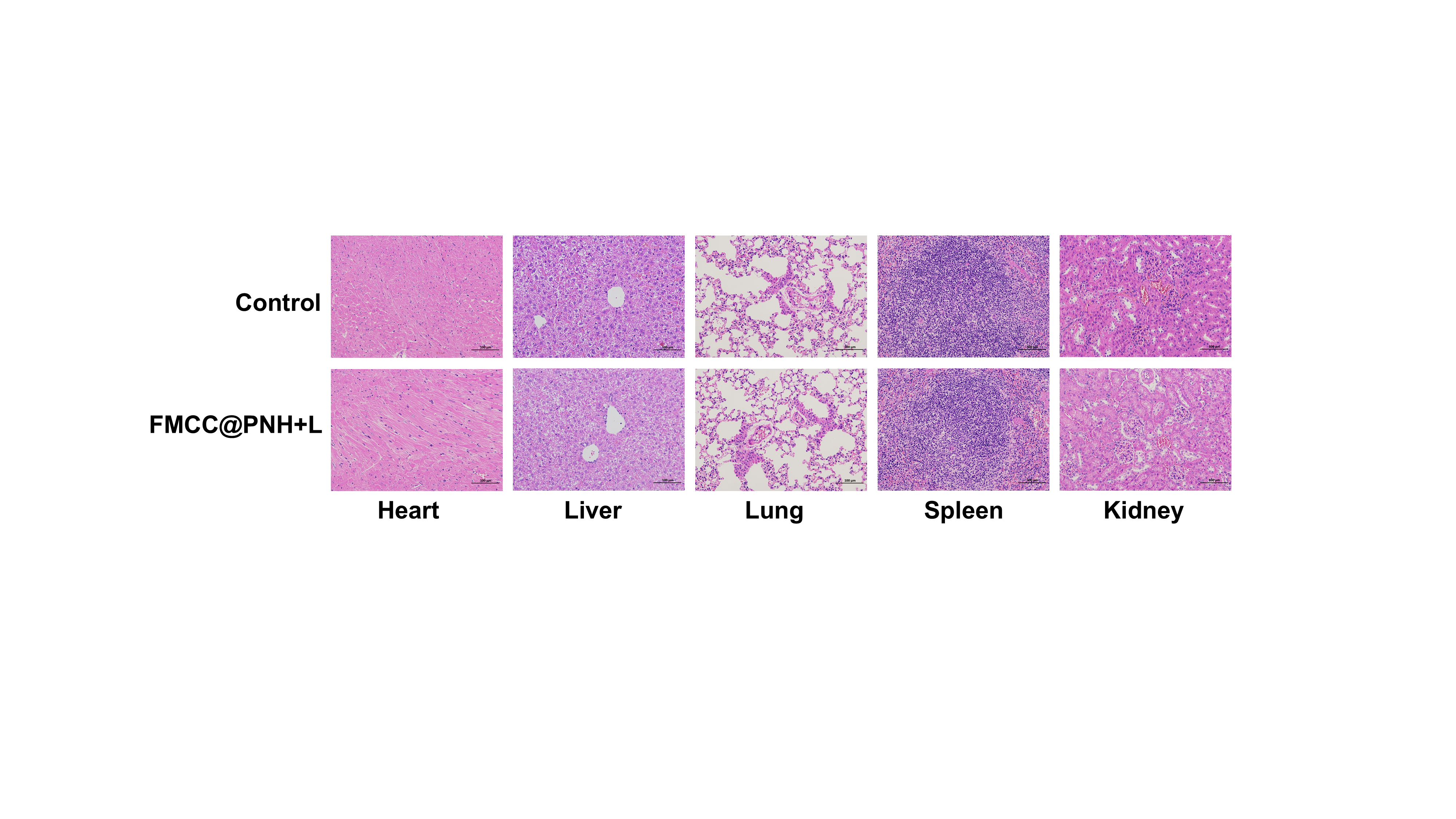


**Figure S18.** Histopathological evaluation by H&E staining of cardiac, hepatic, pulmonary, splenic, and renal tissues in control versus FMCC@PNH+Laser treated groups.


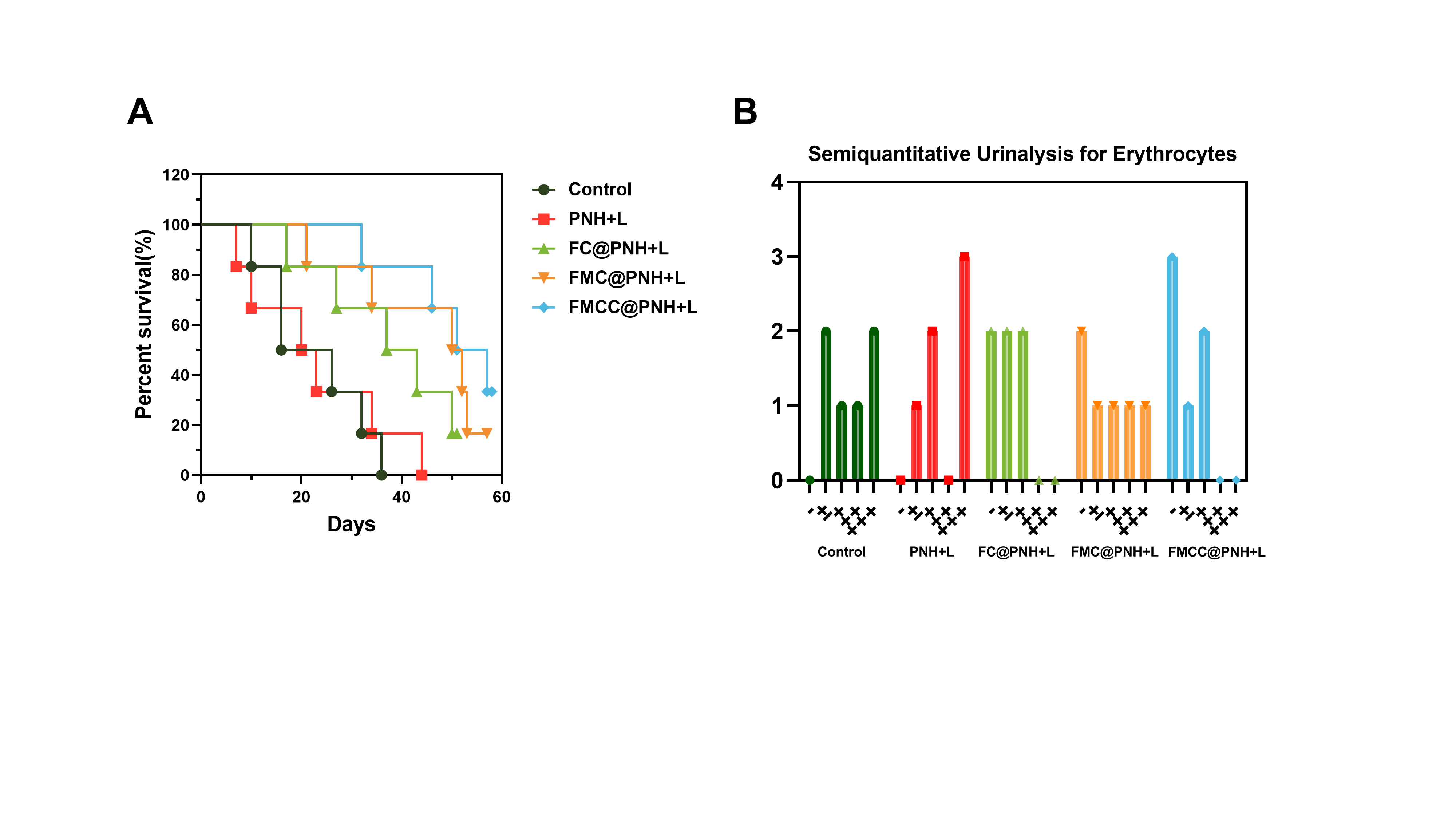


**Figure S19.** Murine Survival Curve in a Bilateral Subcutaneous Bladder Cancer Model.


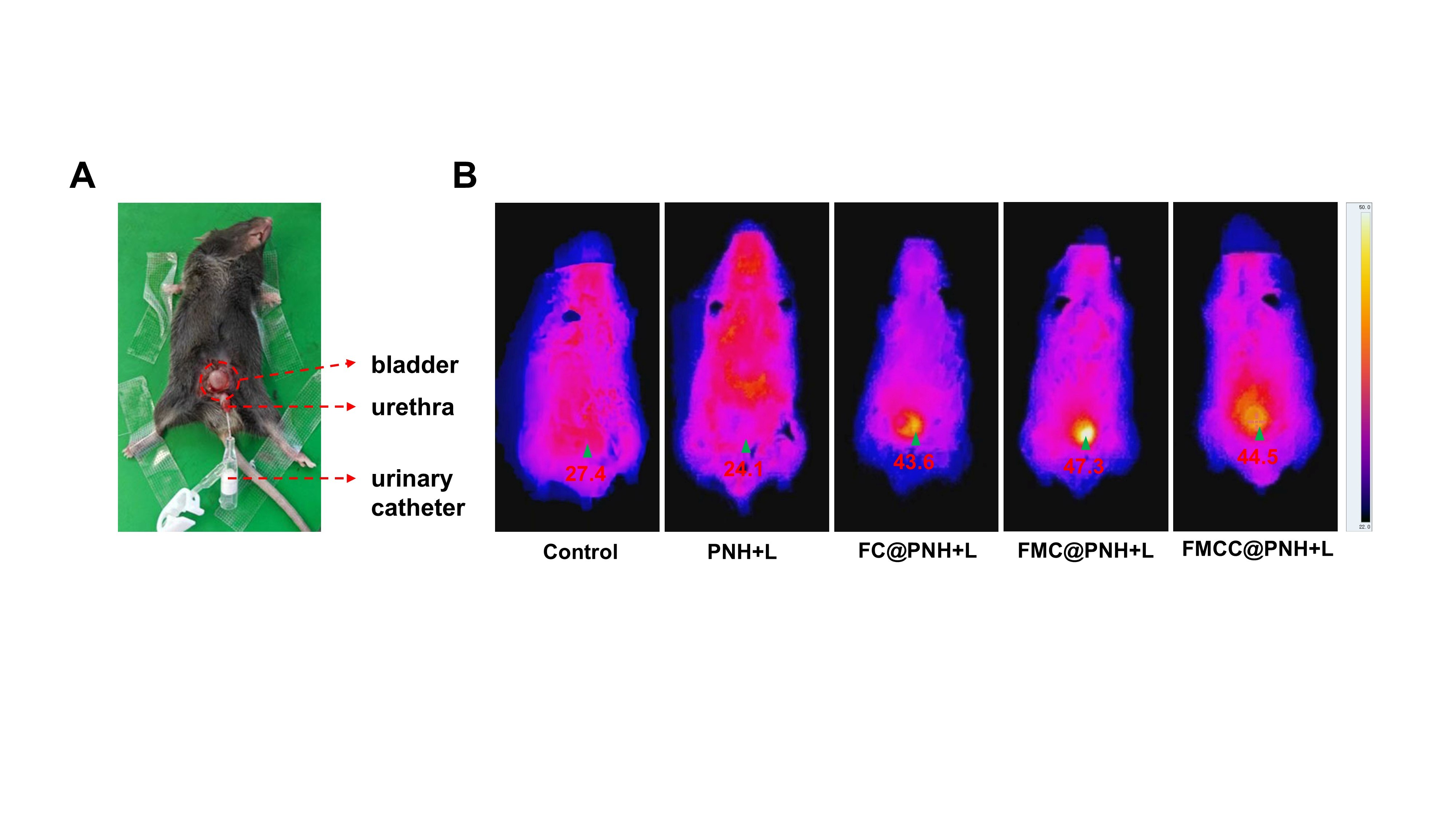


**Figure S20.** Schematic diagrams of intravesical administration and abdominal incision in mice, and photothermal images of orthotopic bladder tumors in mice . (A) Schematic representation of the abdominal incision and intravesical drug administration approach in a murine orthotopic bladder cancer model, the image shows the bladder status after transurethral administration of 100 µL PBS. (B) Infrared thermal images of mice bearing orthotopic bladder tumors after different treatments under 808 nm laser irradiation (1.0 W/cm^2^, 5 min). Groups included Control, PNH+L, FC@PNH+L, FMC@PNH+L, and FMCC@PNH+L. The highest surface temperatures (°C) at the tumor sites are labeled, demonstrating enhanced photothermal conversion efficiency of FMCC@PNH.


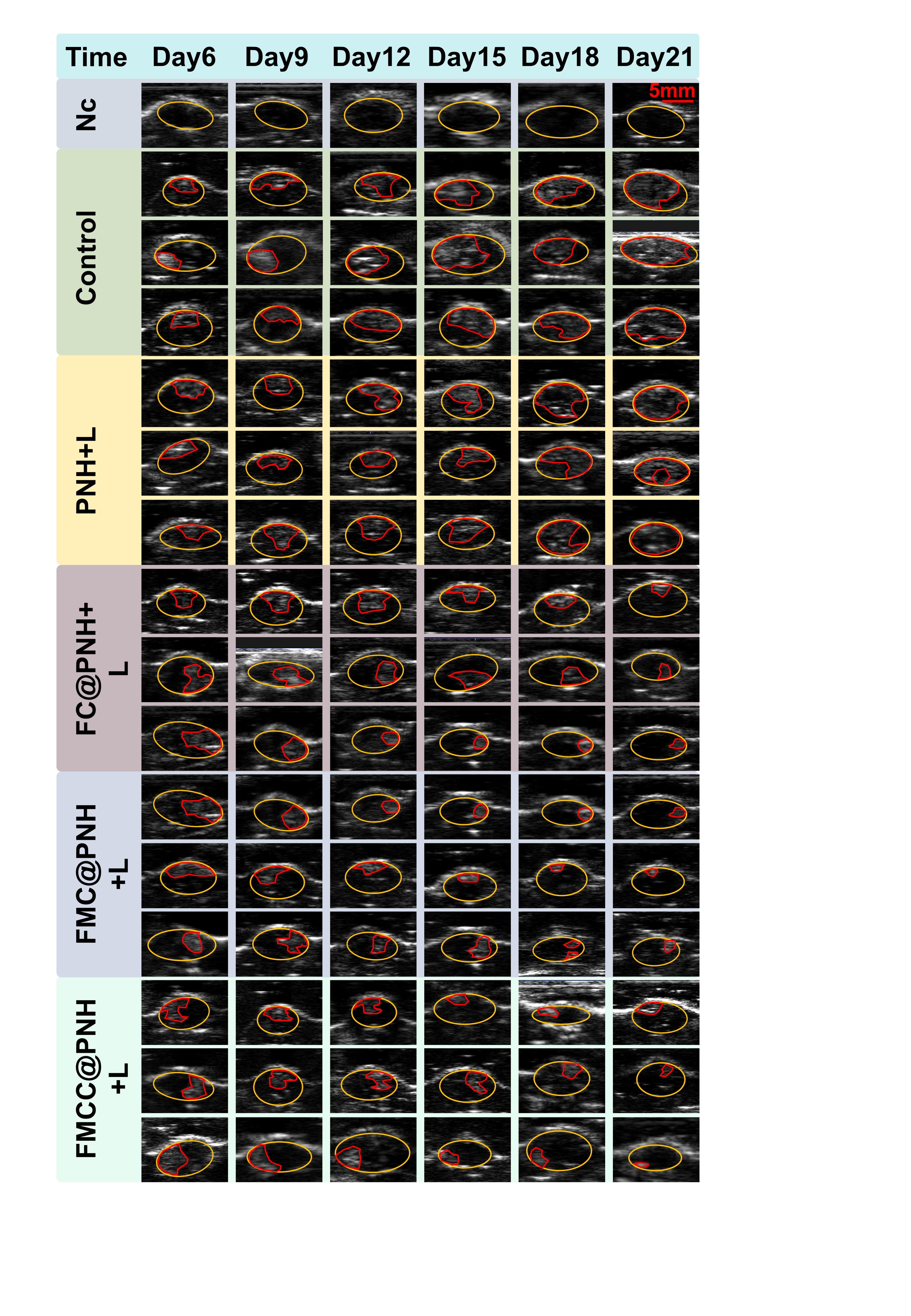


**Figure S21.** Representative bladder ultrasound images of mice from different treatment groups (Nc: blank group, Control, PNH+L, FC@PNH+L, FMC@PNH+L, and FMCC@PNH+L) at various treatment time points.


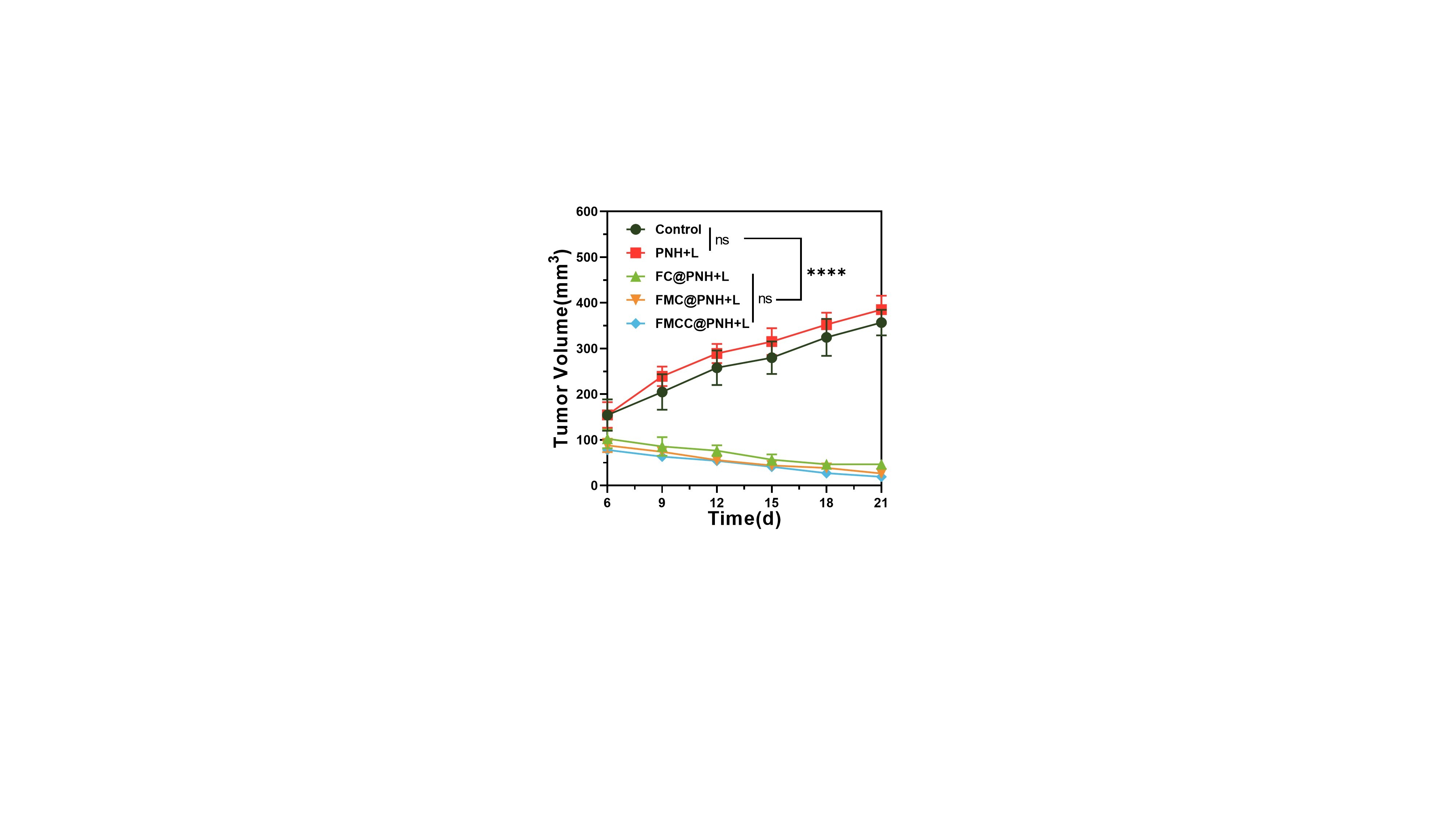


**Figure S22.**  Tumor growth curves showing changes in tumor volume over time.


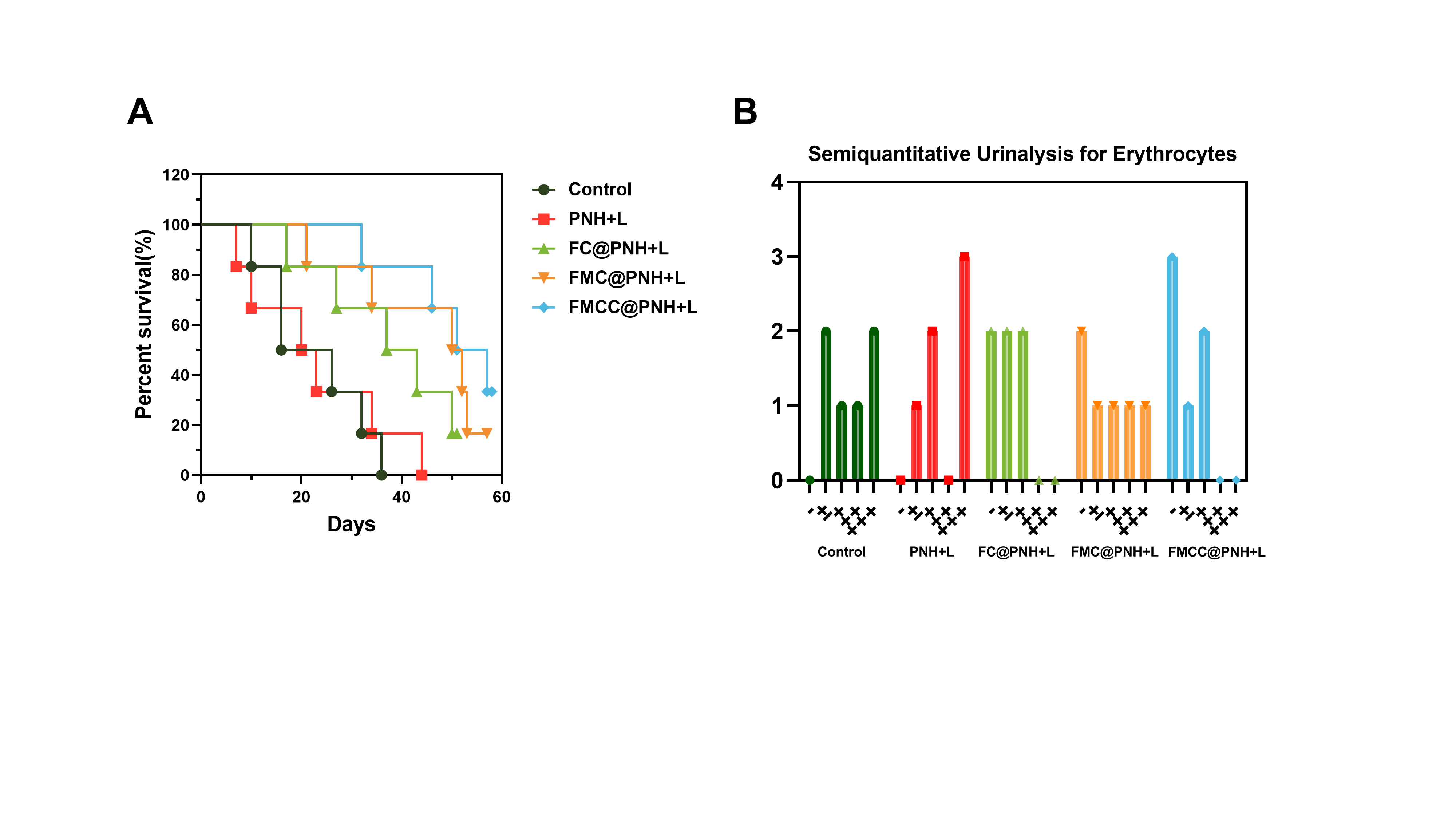


**Figure S23.**  Semi-quantitative evaluation of hematuria in mice bearing orthotopic bladder cancer across different treatment groups.


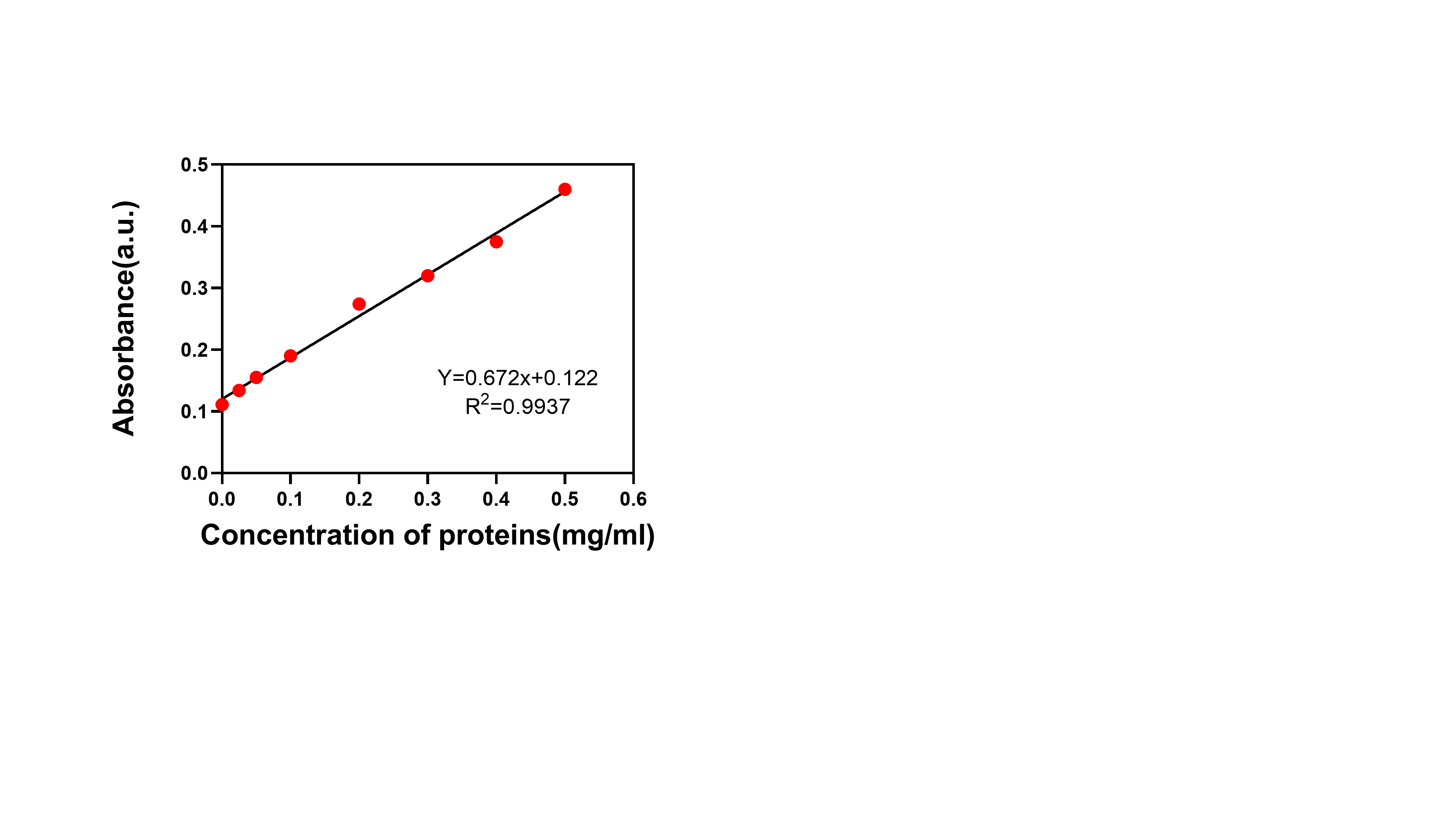


**Figure S24.** Protein quantification by BCA assay with a standard curve.


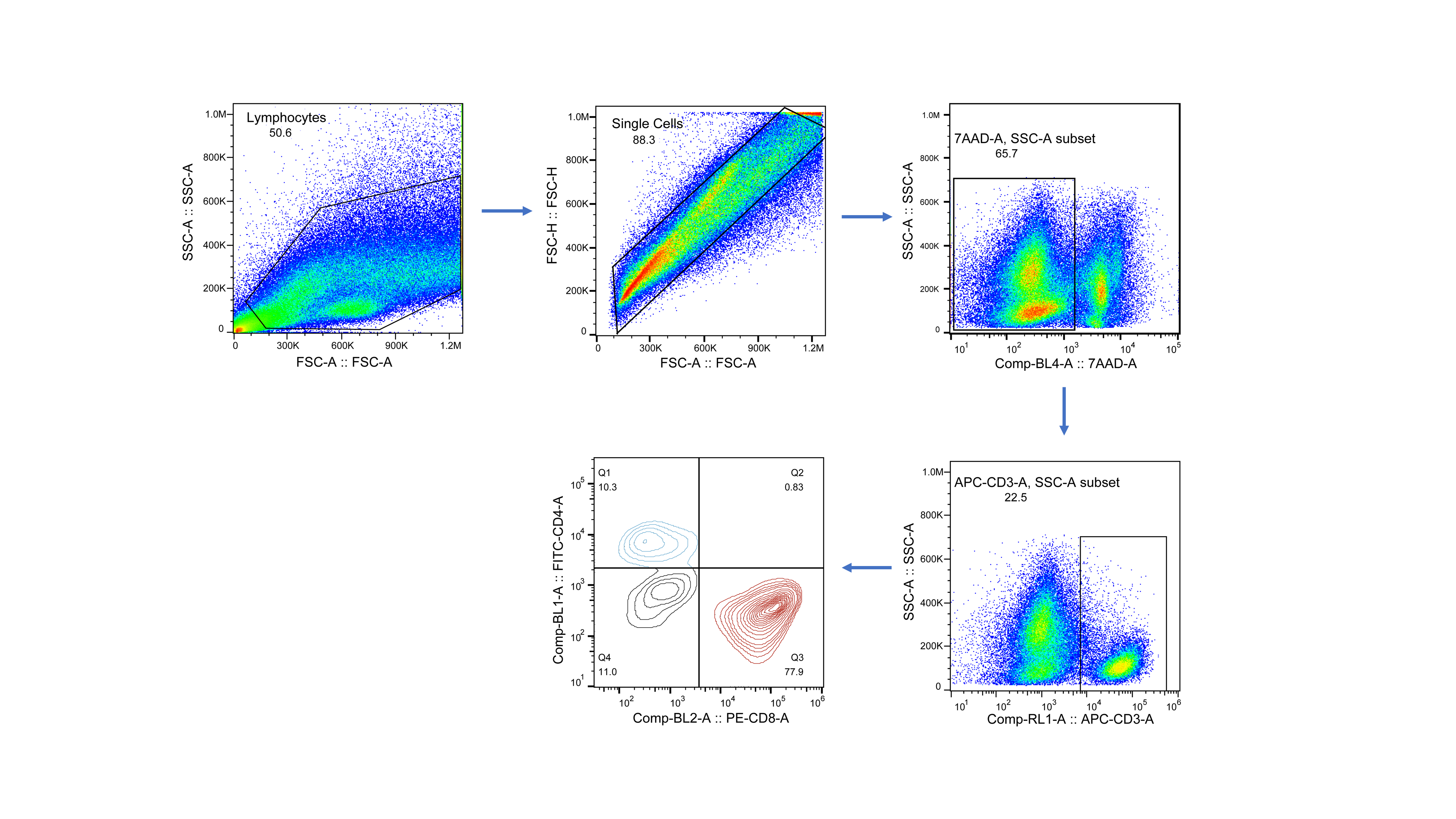


**Figure S25.** Gating strategy for flow cytometry of CD8^+^ cells exhaustion in tumors.


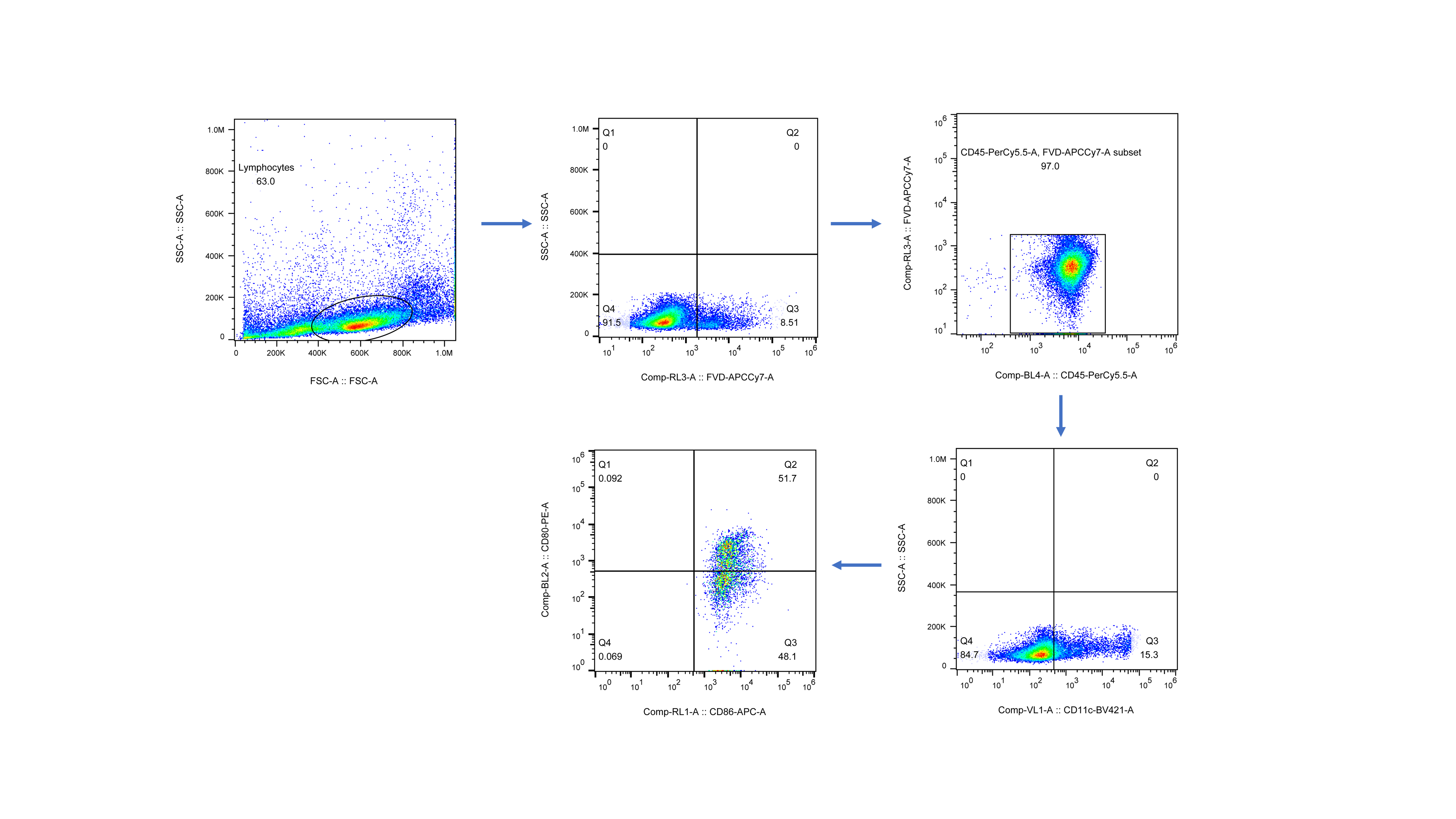


**Figure S26.** Gating strategy for flow cytometry of DCs in lymph nodes.


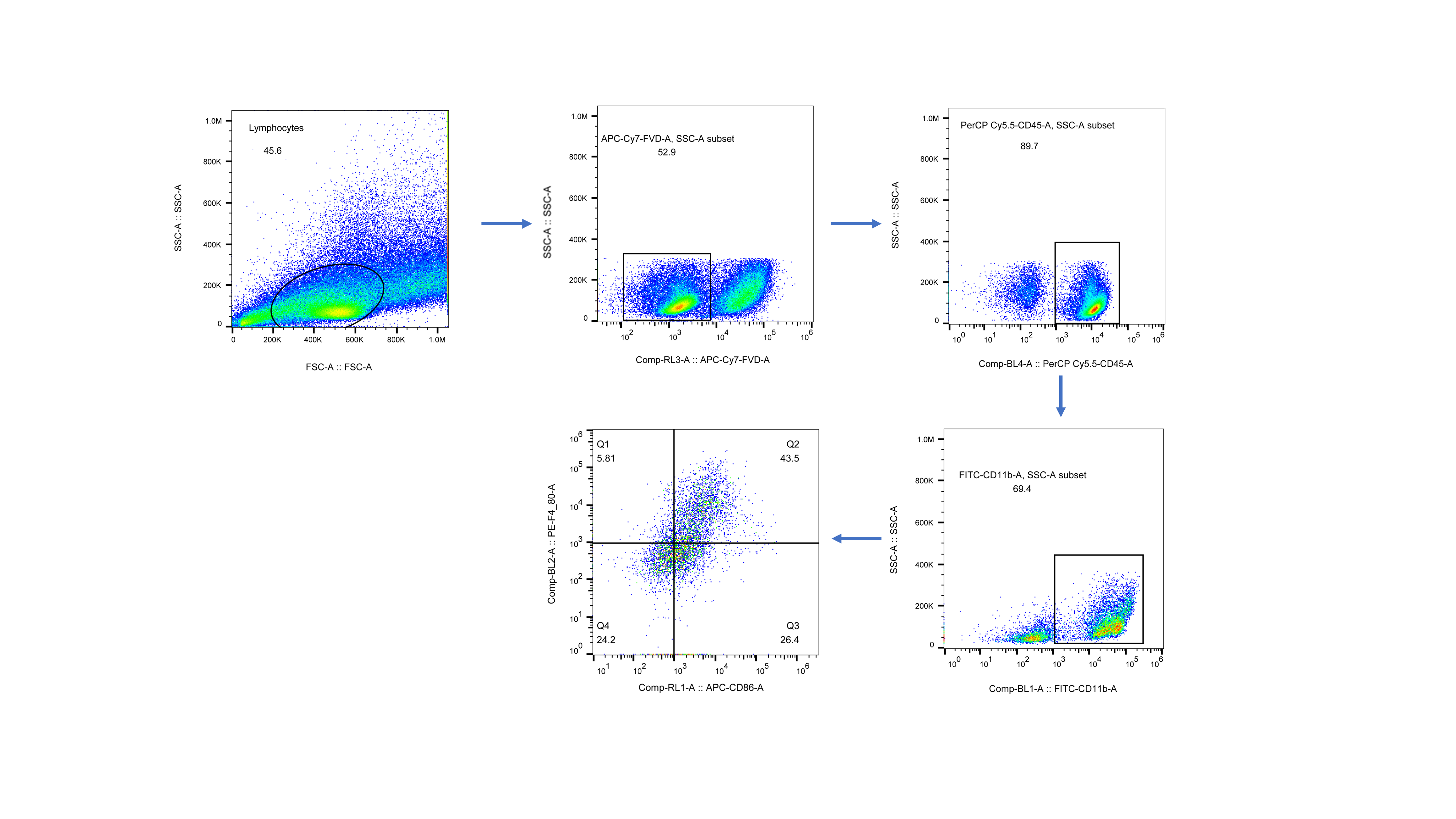


**Figure S27.** Gating strategy for flow cytometry of macrophages in tumors.


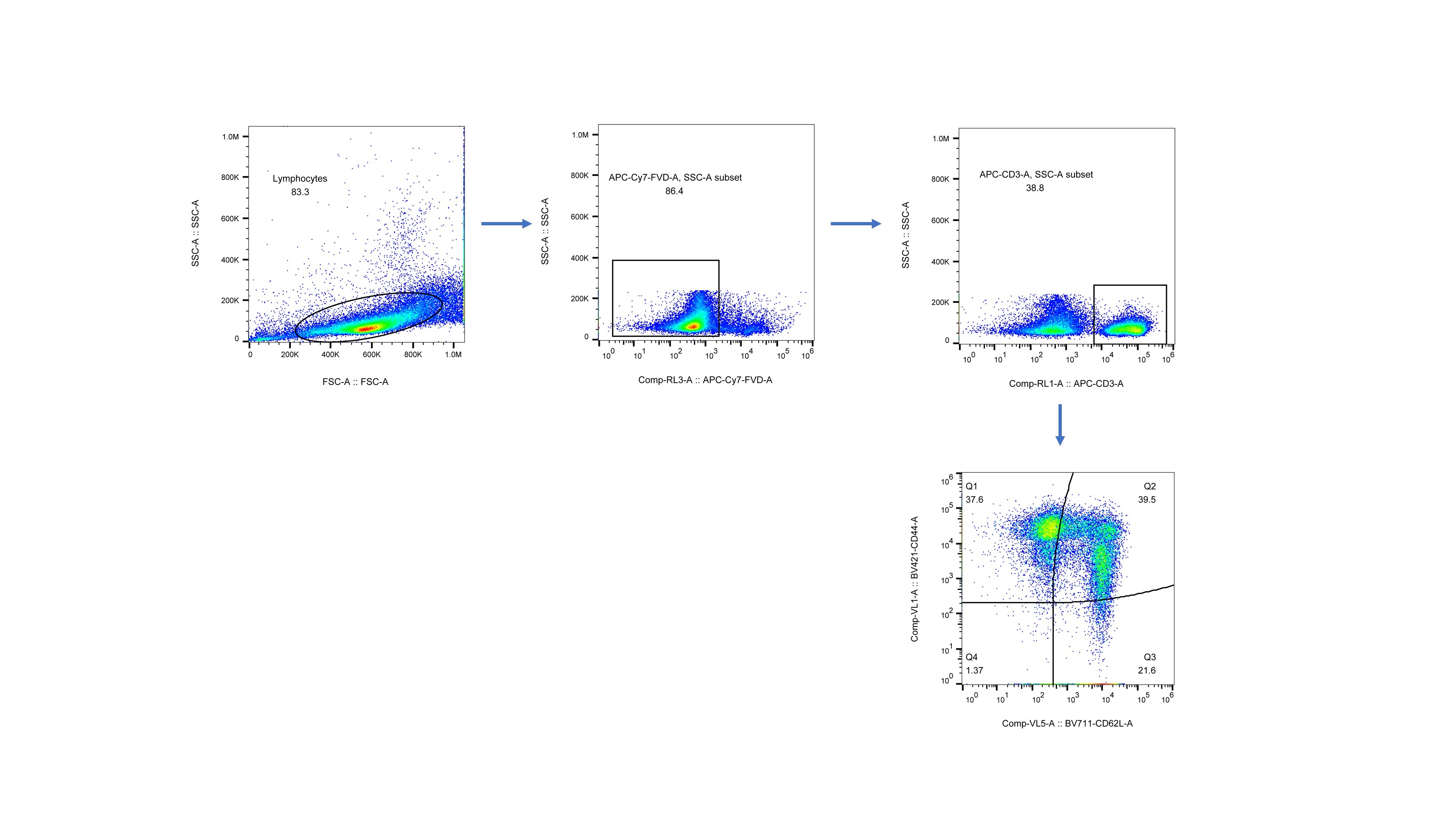


**Figure S28.** Gating strategy for flow cytometry of centralmemory T Cells in spleen.


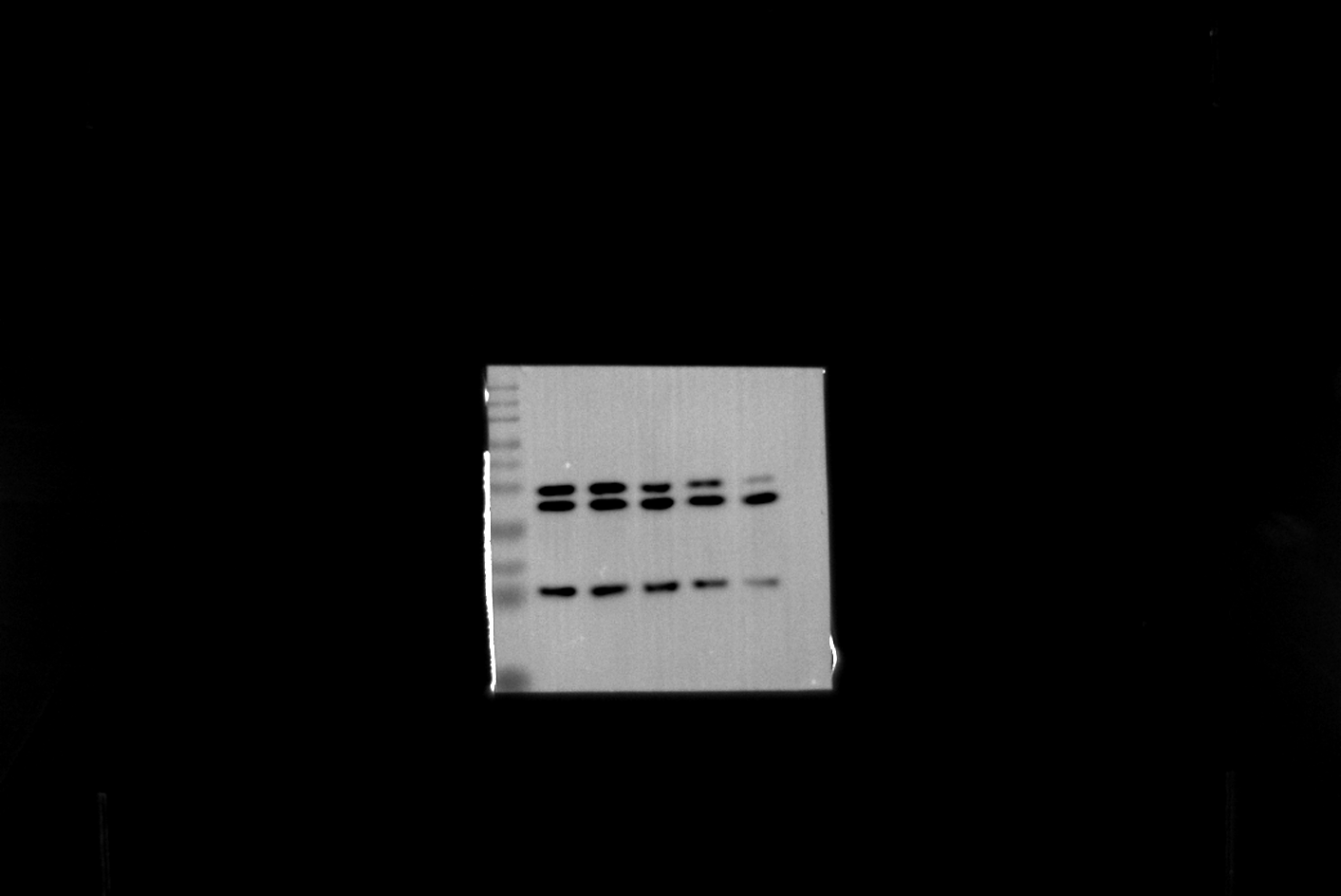


**Figure S29.** Ferroptosis-related proteins and loading control.


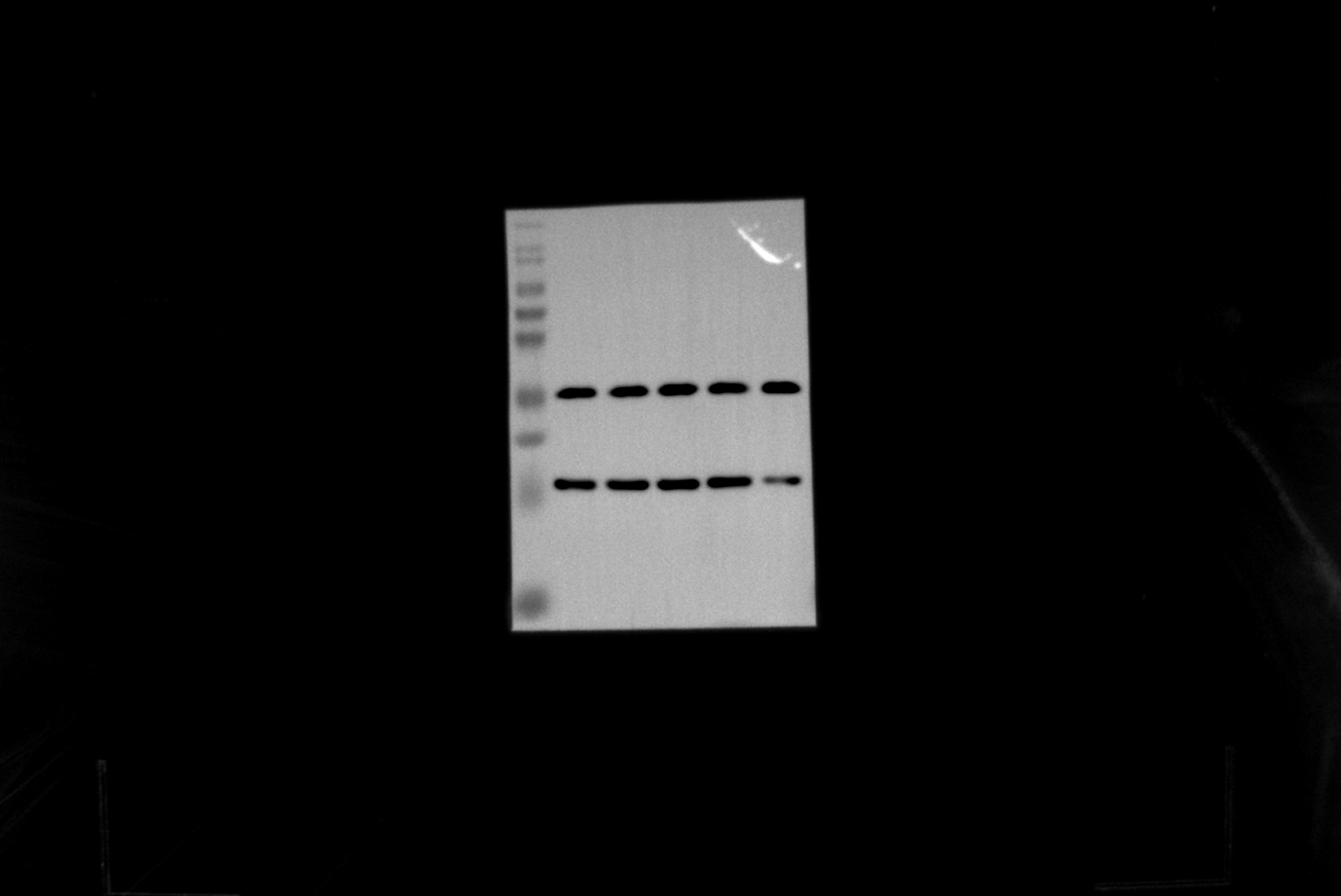


**Figure S30.** Protein expression of CD59 and corresponding internal control.


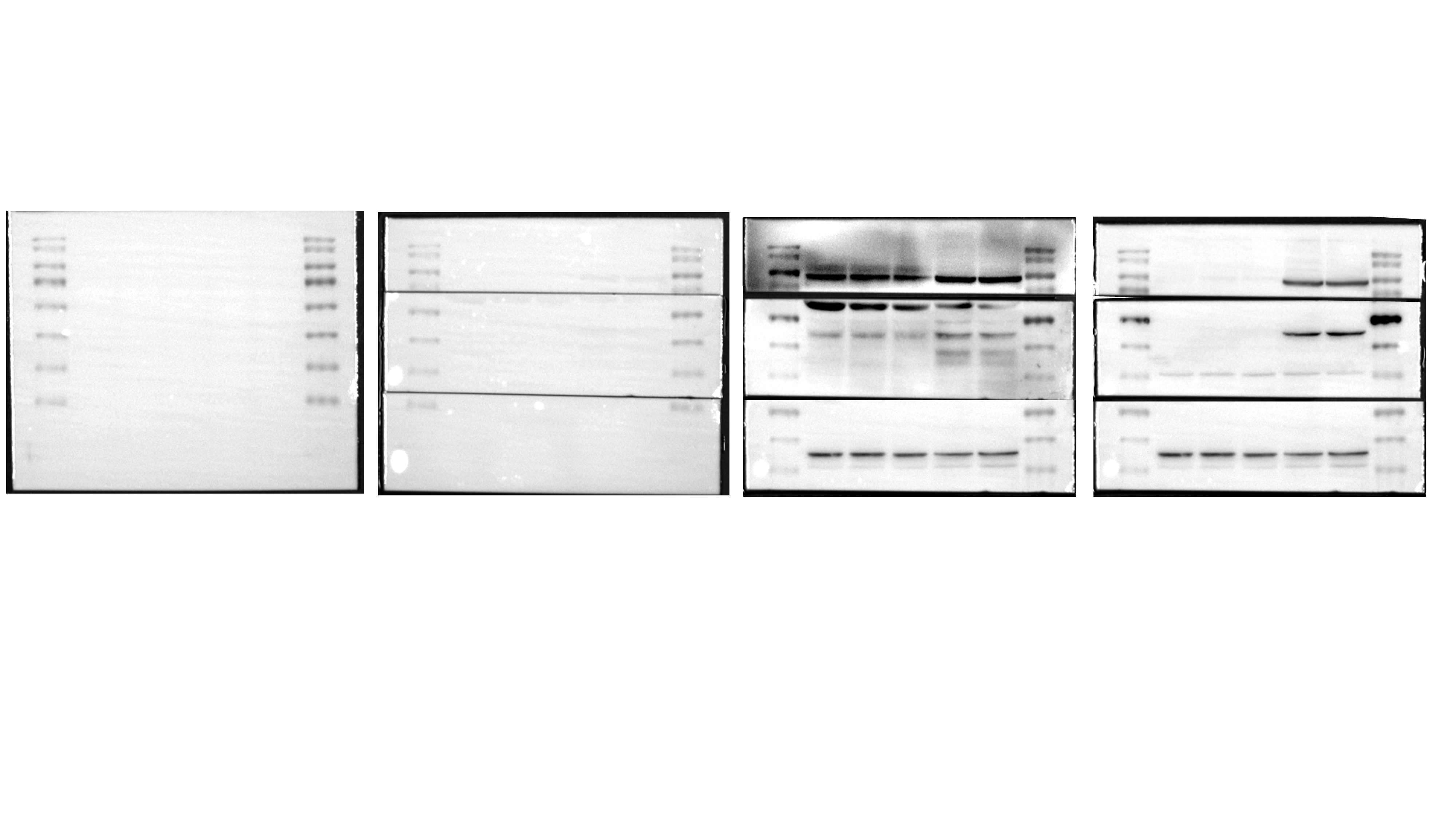


**Figure S31.** Full Western blot membranes showing STING-related proteins and loading controls.


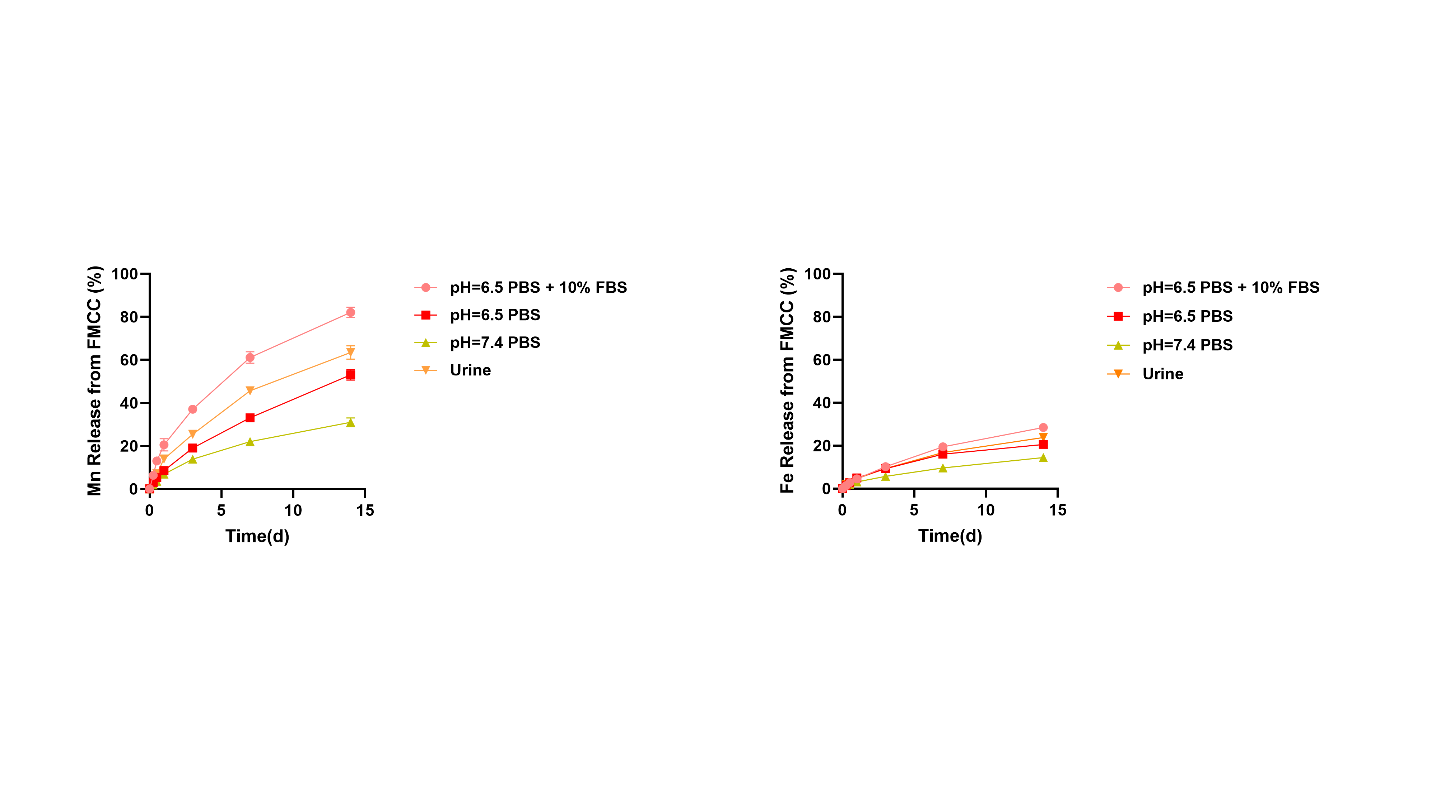


**A**

**A**

**B**

**Figure 32.** Ion release profiles of FMCC nanoparticles under different physiological conditions. (A) Cumulative Mn release from FMCC over 14 days in pH 6.5 PBS + 10% FBS, pH 6.5 PBS, pH 7.4 PBS, and urine. (B) Cumulative Fe release from FMCC under the same conditions. Data are presented as mean ± SD (n = 3).


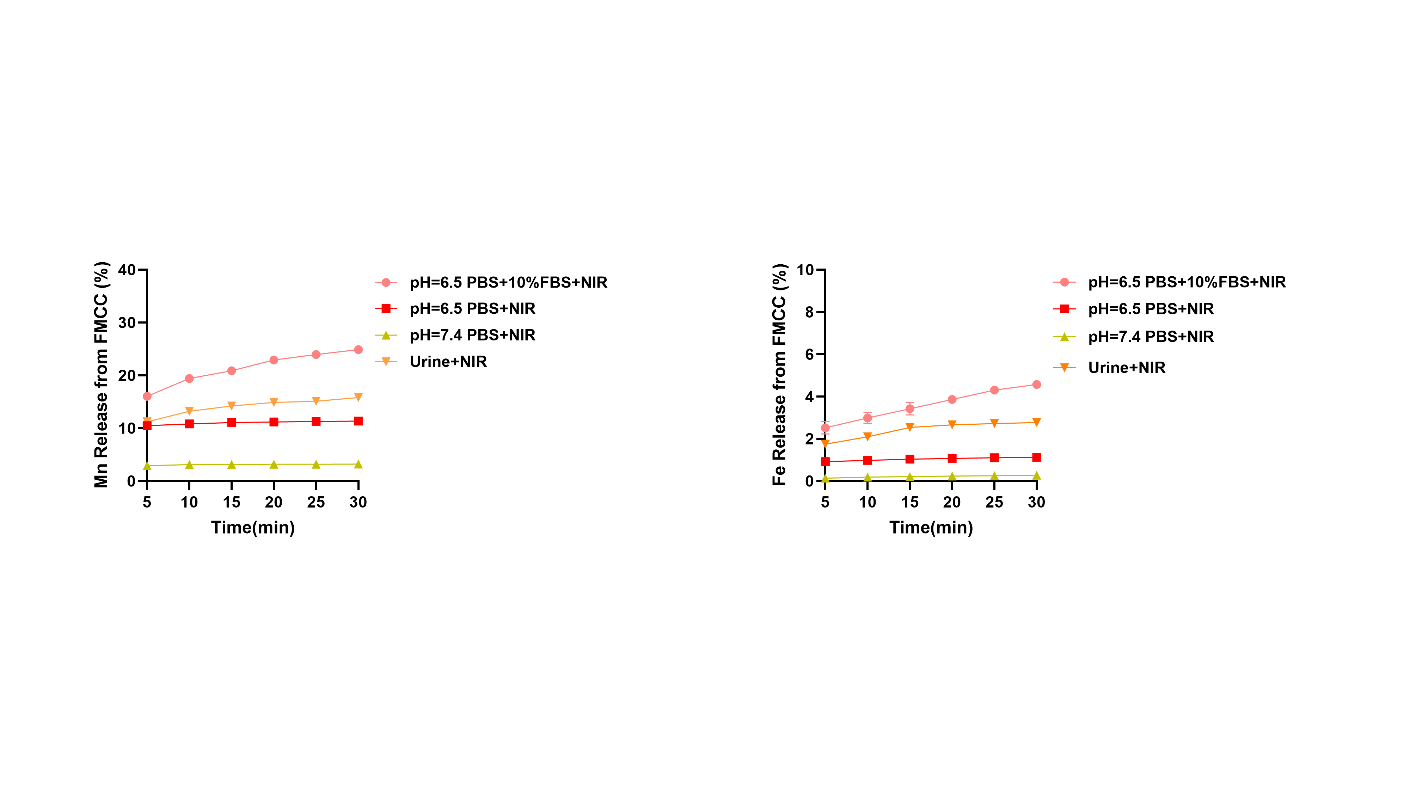


**A**

**B**

**Figure S33.** Time-dependent release profiles of FMCC nanoparticles under NIR irradiation (808 n m). (A) Mn release from FMCC in pH 6.5 PBS + 10% FBS, pH 6.5 PBS, pH 7.4 PBS, and urine. (B) Fe release from FMCC under the same conditions. (n = 3).

**
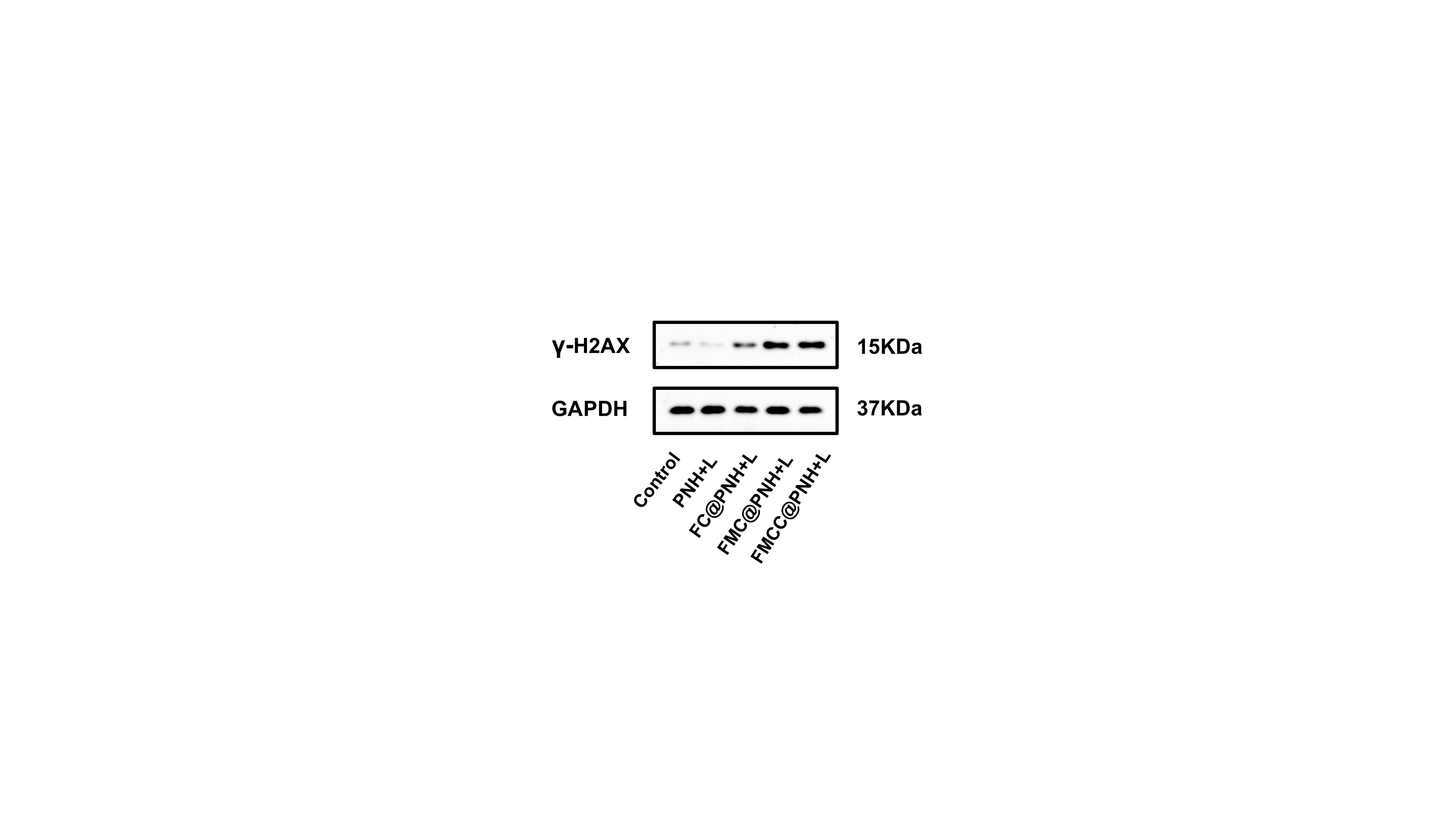
**

**Figure S34.** Expression levels of γ-H2AX in different groups, with GAPDH used as the internal loading control.

**
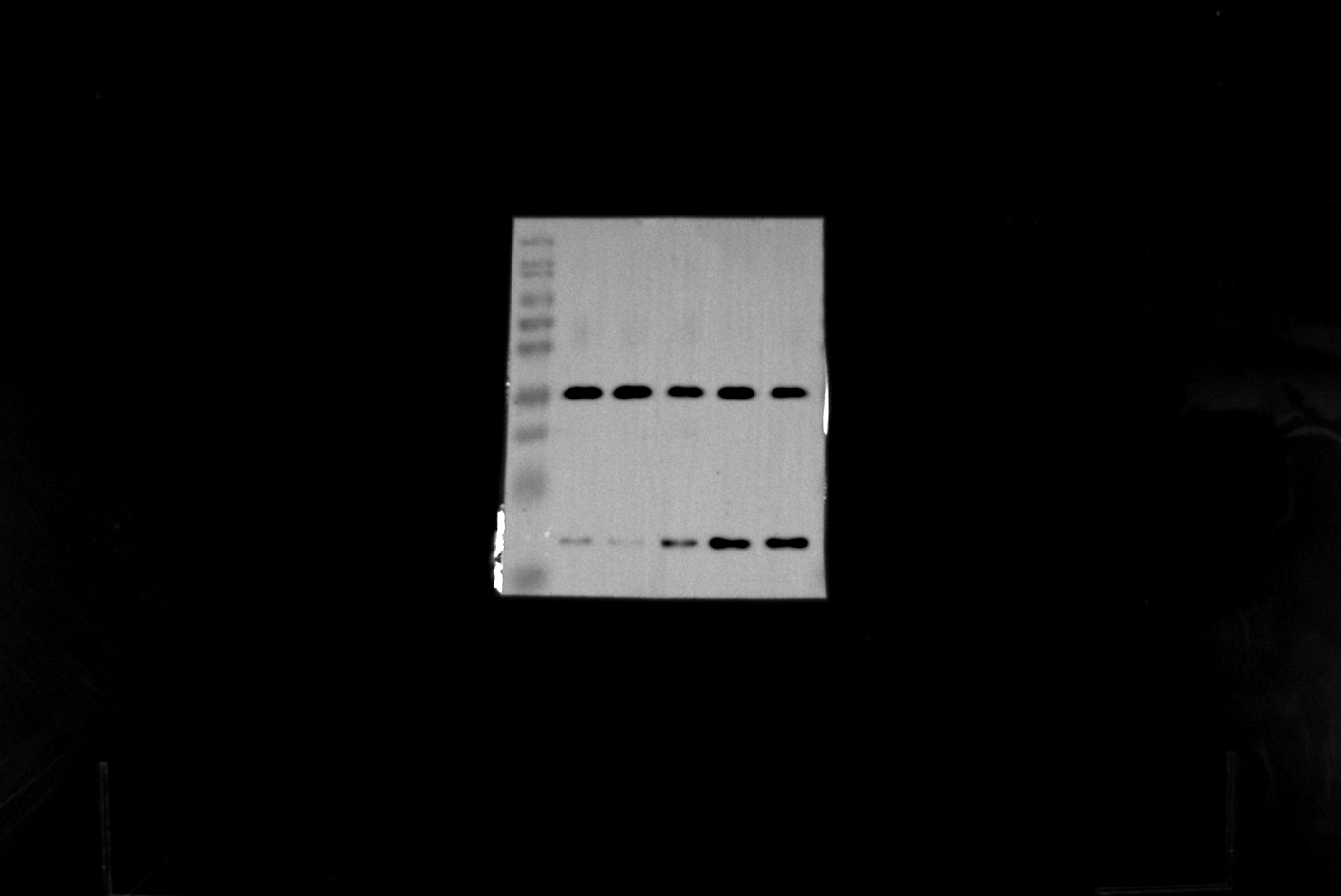
**

**Figure S35.** Uncropped Western blot membranes corresponding to the detection of γ-H2AX in different groups.

**Table S1.**PNH hydrogels with different components and their LCSTs

| Sample | PNIPAM | PNH1 | PNH2 | PNH3 | PNH4 |
| --- | --- | --- | --- | --- | --- |
| NIPAM/% | 100 | 90 | 85 | 80 | 75 |
| NHMAM/% | 0 | 10 | 15 | 20 | 25 |
| LCST/°C | 35 | 39 | 45 | 52 | 57 |

**Table S2.** Components used to synthesize PNH hydrogels

| Sample | NIPAM(mg) | NHMAM(mg) | BIS(mg) | TEMED(mg) | H_2_O(ml) |
| --- | --- | --- | --- | --- | --- |
| PNIPAM | 500 | 0 | 25 | 20 | 9 |
| PNH1 | 450 | 50 | 25 | 20 | 9 |
| PNH2 | 425 | 75 | 25 | 20 | 9 |
| PNH3 | 400 | 100 | 25 | 20 | 9 |
| PNH4 | 375 | 125 | 25 | 20 | 9 |

**Table S3.** The brand, clone, and catalog number of all antibodies used in flow cytometry

| Antibodies | Brand | Catalog number | Clone |
| --- | --- | --- | --- |
| PE anti-mouse CD8a Antibody | Biolegend | 100707 | 53-6.7 |
| APC anti-mouse CD3ε Antibody | Biolegend | 100311 | 145-2C11 |
| FITC anti-mouse CD4 Antibody | Biolegend | 100509 | RM4-5 |
| ruStain FcX™ PLUS (anti-mouse CD16/32) Antibody | Biolegend | 156603 | S17011E |
| PerCP/Cyanine5.5 anti-mouse CD45 Antibody | Biolegend | 103131 | 30-F11 |
| PE anti-mouse CD80 Antibody | Biolegend | 104707 | 16-10A1 |
| APC anti-mouse CD86 Antibody | Biolegend | 105011 | GL-1 |
| Brilliant Violet 421™ anti-mouse CD11c Antibod BV42 | Biolegend | 117329 | N418 |
| Fixable Viability Dye eFluor™ 780 AP | eBioscience™ | 65-0865 | / |
| TruStain FcX™ PLUS (anti-mouse CD16/32) Antibody | Biolegend | 156603 | S17011E |
| Brilliant Violet 421™ anti-mouse/human CD44 An BV421 | Biolegend | 103039 | IM7 |
| Brilliant Violet 711™ anti-mouse CD62L Antibod BV711 | Biolegend | 104445 | MEL-14 |
| FITC anti-mouse/human CD11b Antibody | Biolegend | 101205 | M1/70 |
| PE anti-mouse F4/80 Antibody | Biolegend | 123109 | BM8 |
| FITC Plus Anti-Mouse CD86 | proteintech | FITC-65068 | GL1 |
| APC Anti-Mouse CD80 | proteintech | 65076 | 16-10A1 |
| FITC Plus Anti-Mouse F4/80 Rabbit Recombinant Antibody | proteintech | 98236 | 241959G4 |
| PE Anti-Mouse CD86 | proteintech | PE-65068 | GL1 |

**Reference**

[1] Y. Li, S. Shan, R. Zhang, C. Sun, X. Hu, J. Fan, Y. Wang, R. Duan, M. Gao,"*Imaging and Downstaging Bladder Cancer with the <SUP>177</SUP>Lu-Labeled Bioorthogonal Nanoprobe"*, *Acs Nano* **2024**, *18* (26), 17209, <https://doi.org/10.1021/acsnano.4c04303>.
